# Trends in cardiovascular risk factor prevalence, treatment, and control among US adolescents aged 12 to 19 years, 2001 to March 2020

**DOI:** 10.1186/s12916-024-03453-5

**Published:** 2024-06-13

**Authors:** Qiang Qu, Qixin Guo, Jinjing Shi, Ziqi Chen, Jinyu Sun, Iokfai Cheang, Rongrong Gao, Yanli Zhou, Haifeng Zhang, Shengen Liao, Wenming Yao, Xinli Li

**Affiliations:** 1 Present Address: State Key Laboratory for Innovation and Transformation of Luobing Theory, Department of Cardiology, The First Affiliated Hospital of Nanjing Medical University, 300 Guangzhou Road, Nanjing, 210029 China; 2 Department of Cardiology, Gusu School, The Affiliated Suzhou Hospital of Nanjing Medical University, Suzhou Municipal Hospital, Nanjing Medical University, 26 Daoqian Street, Suzhou, 215002 China; 3 Department of Cardiology, Jiangsu Province Hospital, 300 Guangzhou Road, Nanjing, 210029 China

**Keywords:** Cardiovascular risk factor, Prevalence, Treatment, Control, Pediatrics, NHANES

## Abstract

**Background:**

Early-life cardiovascular risk factors (CVRFs) are known to be associated with target organ damage during adolescence and premature cardiovascular morbidity and mortality during adulthood. However, contemporary data describing whether the prevalence of CVRFs and treatment and control rates have changed are limited. This study aimed to examine the temporal trends in the prevalence, treatment, and control of CVRFs among US adolescents over the past 2 decades.

**Methods:**

This is a serial cross-sectional study using data from nine National Health and Nutrition Examination Survey cycles (January 2001—March 2020). US adolescents (aged 12 to 19 years) with information regarding CVRFs (including hypertension, elevated blood pressure [BP], diabetes, prediabetes, hyperlipidemia, obesity, overweight, cigarette use, inactive physical activity, and poor diet quality) were included. Age-adjusted trends in CVRF prevalence, treatment, and control were examined. Joinpoint regression analysis was performed to estimate changes in the prevalence, treatment, and control over time. The variation by sociodemographic characteristics were also described.

**Results:**

A total of 15,155 US adolescents aged 12 to 19 years (representing ≈ 32.4 million people) were included. From 2001 to March 2020, there was an increase in the prevalence of prediabetes (from 12.5% [95% confidence interval (CI), 10.2%-14.9%] to 37.6% [95% CI, 29.1%-46.2%]) and overweight/obesity (from 21.1% [95% CI, 19.3%-22.8%] to 24.8% [95% CI, 21.4%-28.2%]; from 16.0% [95% CI, 14.1%-17.9%] to 20.3% [95% CI, 17.9%-22.7%]; respectively), no improvement in the prevalence of elevated BP (from 10.4% [95% CI, 8.9%-11.8%] to 11.0% [95% CI, 8.7%-13.4%]), diabetes (from 0.7% [95% CI, 0.2%-1.2%] to 1.2% [95% CI, 0.3%-2.2%]), and poor diet quality (from 76.1% [95% CI, 74.0%-78.2%] to 71.7% [95% CI, 68.5%-74.9%]), and a decrease in the prevalence of hypertension (from 8.1% [95% CI, 6.9%-9.4%] to 5.5% [95% CI, 3.7%-7.3%]), hyperlipidemia (from 34.2% [95% CI, 30.9%-37.5%] to 22.8% [95% CI, 18.7%-26.8%]), cigarette use (from 18.0% [95% CI, 15.7%-20.3%] to 3.5% [95% CI, 2.0%-5.0%]), and inactive physical activity (from 83.0% [95% CI, 80.7%-85.3%] to 9.5% [95% CI, 4.2%-14.8%]). Sex and race/ethnicity affected the evolution of CVRF prevalence differently. Whilst treatment rates for hypertension and diabetes did not improve significantly (from 9.6% [95% CI, 3.5%-15.8%] to 6.0% [95% CI, 1.4%-10.6%]; from 51.0% [95% CI, 23.3%-78.7%] to 26.5% [95% CI, 0.0%-54.7%]; respectively), BP control was relatively stable (from 75.7% [95% CI, 56.8%-94.7%] to 73.5% [95% CI, 40.3%-100.0%]), while glycemic control improved to a certain extent, although it remained suboptimal (from 11.8% [95% CI, 0.0%-31.5%] to 62.7% [95% CI, 62.7%-62.7%]).

**Conclusions:**

From 2001 to March 2020, although prediabetes and overweight/obesity increased, hypertension, hyperlipidemia, cigarette use, and inactive physical activity decreased among US adolescents aged 12 to 19 years, whereas elevated BP, diabetes, and poor diet quality remained unchanged. There were disparities in CVRF prevalence and trends across sociodemographic subpopulations. While treatment and control rates for hypertension and diabetes plateaued, BP control were stable, and improved glycemic control was observed.

**Supplementary Information:**

The online version contains supplementary material available at 10.1186/s12916-024-03453-5.

## Background

Cardiovascular diseases (CVDs) have been the leading cause of deaths and disabilities for several decades [1]. Despite substantial declines in cardiovascular mortality since 1950, CVDs remain the most common source of mortality in the US and accounts for up to 928,741 deaths in 2020 [2]. CVDs are estimated to result in direct and indirect costs of over $407.3 billion between 2018 and 2019, constituting a considerable economic and social burden [3].

Recent meta-analyses show that a substantial proportion of boys (6%-39%) and girls (6%-86%) exhibit cardiovascular risk factors (CVRFs) [4]. Boys and girls with poor health status have 5.7- and 3.6-fold higher CVD risks, respectively [4]. Among adolescents, CVRFs are strongly correlated with early indicators of target organ damage [5–8]. Without appropriate and timely intervention, conventional CVRFs (including hypertension, elevated blood pressure [BP], diabetes, prediabetes, hyperlipidemia, obesity, overweight, cigarette use, inactive physical activity, and poor diet quality) during adolescence may persist [9, 10], and are associated with higher mortality and morbidity risks during adulthood [11–19]. Thus, mitigating CVRFs in adolescents could potentially result in a reduced number of individuals affected by CVDs later in life. The magnitude of CVRF burden in adolescents may have an increasingly profound impact on public health as the population ages. However, trends in CVRF prevalence, treatment, and control in US adolescents are as yet unclear. Moreover, most previous studies do not provide recent estimated rates, ignore information on several important subgroups (i.e., sociodemographic status), or have methodological limitations (e.g., unweighted or unadjusted estimates for design variables) [20–24], which are essential for predicting population-level complications and helping develop effective national public health policies [25, 26].

The primary purpose of this study was to determine how CVRF prevalence changed among US adolescents aged 12 to 19 years in the past 2 decades. A secondary purpose was to determine whether CVRF treatment and control rates were improved or deteriorative during this period, and whether these trends varied across sociodemographic subgroups. To achieve these goals, data from 9 cycles of the National Health and Nutrition Examination Survey (NHANES) were analyzed.

## Methods

### Data collection

The NHANES, administered by the National Center for Health Statistics (NCHS) of the Centers for Disease Control and Prevention (CDC), comprises a series of publicly available, cross-sectional, nationwide surveys of civilian, noninstitutionalized US population [27, 28]. To obtain nationally representative estimates, the NHANES selects participants aged 1 to 80 years using a complex, multistage, probability sampling design. Most questionnaire data are collected through in-home interviews, and physical examinations and laboratory testing data are collected through mobile examinations; the data are released every 2 years since 1999. Details regarding design, weighting, and methodology have been described elsewhere [29]. This study included 9 cycles from 2001–2002 to 2017-March 2020, focusing on participants aged 12–19 years. Among the adolescent participants, unweighted response rates ranged from 54.4% to 88.9% for in-home interviews and 50.7% to 86.4% for mobile examinations (Additional file 1: eTable 1). The NCHS Ethics Review Board approved the study protocol, and written informed consent was acquired from all individuals [30]. The Strengthening the Reporting of Observational Studies in Epidemiology (STROBE) reporting guideline was followed throughout the study (Additional file 1: eTable 2) [31].

During the in-home interviews, information regarding age, sex, race/ethnicity, educational level, income to poverty ratio (IPR), insurance status, and medical conditions (including medical history and medication use) was collected. Race/ethnicity categories included non-Hispanic White, non-Hispanic Black, Mexican American, and other races/ethnicities (including other Hispanic and other/multiple races or ethnicities). Medical history and medication use were self-reported. Medication use was determined by the responses to questions about taking prescription drugs. Participants who answered ‘yes’ were asked to show all drug containers; when not available, participants were asked to verbally list all drug names.

Participants were asked whether they had ever smoked cigarettes, how old they were when they first smoked, and whether they smoked during the past month to determine smoking status. Participants were also asked to report the frequency and duration of moderate- and, separately, vigorous-intensity physical activity (including work- and transportation-related and leisure-time domains) during a typical week. Weekly exercise time was calculated as the minutes of moderate-intensity physical activity plus twice the minutes of vigorous-intensity physical activity per week. Dietary information was obtained from 24-h recall interviews during the mobile examinations. The simple Healthy Eating Index-2015 (HEI-2015) scoring algorithm (per day), based on 13 dietary components (including total fruit, whole fruit, total vegetables, greens or beans, whole grains, dairy, total protein foods, seafood or plant protein, fatty acids, refined grains, sodium, added sugars, and saturated fat), was used to indicate overall diet quality [32]. Anthropometry parameters including weight, height, and BP were measured using standard protocols. Body mass index (BMI) was calculated by dividing weight by height squared. Systolic and diastolic BP were calculated as the mean of 3 (sometimes 4) BP determinations.

Non-fasting laboratory testing was used to measure serum levels of hemoglobin A_1c_ (HbA_1c_), total cholesterol (TC) and high-density lipoprotein cholesterol (HDL-C). The non-high-density lipoprotein cholesterol (non-HDL-C) was calculated by subtracting HDL-C from TC. Approximately half of the participants were sampled to attend a morning examination, during which fasting plasma glucose (FPG), low-density lipoprotein cholesterol (LDL-C), and triglycerides levels were measured after fasting for 8.5 to less than 24 h.

### Assessment of CVRF prevalence, treatment, and control

The prevalence of CVRFs was evaluated through the above parameters derived from in-home interviews and mobile examinations. According to the 2017 American Academy of Pediatrics and 2017 American College of Cardiology/American Heart Association guidelines [33, 34], age-, sex-, and height-specific BP percentiles and 130/80 mmHg were used to define high BP in adolescents aged < 18 and 18–19 years, respectively (Additional file 1: eTable 3). Hypertension was defined as stage 1 or 2 levels and/or current use of antihypertensive medications (Additional file 1: eMethod 1), whereas elevated BP was defined as an elevated level. Diabetes was defined as a HbA_1c_ of ≥ 6.5%, FPG of ≥ 126 mg/dL, self-report of previous diagnosis, and/or current use of antidiabetic medications, whereas prediabetes was defined as a HbA_1c_ of 5.7%-6.4% and/or FPG of 100–125 mg/dL [35]. Hyperlipidemia was defined as a TC of ≥ 200 mg/dL, HDL-C of < 40 mg/dL, non-HDL-C of ≥ 145 mg/dL, LDL-C of ≥ 130 mg/dL, triglycerides of ≥ 130 mg/dL, and/or current use of antihyperlipidemic medications [36, 37]. Obesity and overweight were defined based on BMI using the Lambda Mu Sigma method [38]. In accordance with CDC’s standard [39, 40], cigarette use was defined as smoking cigarettes within the previous 30 days. Based on the 2018 Physical Activities Guidelines for Americans [41], inactive physical activity was defined as a weekly exercise time of < 420 and < 150 min/wk in adolescents aged < 18 and 18–19 years, respectively. Diet quality was broadly classified according to HEI-2015, and a score of < 51 points was considered as poor diet quality [42]. Consistent definitions were applied for the CVRFs throughout the study.

CVRF treatment and control rates were also assessed in adolescents aged 12–19 years. Hypertension treatment was defined as current use of antihypertensive medications. Hypertension was considered controlled if (1) BP was reduced to < 90th percentile in adolescents aged < 13 years, (2) BP was reduced to < 90th percentile and < 130/80 mmHg in adolescents aged 13–17 years, or (3) BP was reduced to < 130/80 mmHg in adolescents aged 18–19 years [33, 34]. Diabetes treatment was defined as current use of antidiabetic medications. Diabetes was considered controlled if HbA_1c_ was reduced to < 7% [43, 44]. Hyperlipidemia treatment and control rates were not analyzed because the updated guidelines no longer recommended lipid-level targets for treatment and the number of adolescents receiving lipid-lowering medications was small [36, 37].

### Statistical analysis

To ensure that the estimates accurately represented the noninstitutionalized US population, weights for the interview sample, examination sample, fasting subsample, and dietary sample were appropriately used for all analyses. Baseline characteristics of adolescents aged 12–19 years were presented as means or proportions with 95% confidence intervals (CIs). A linear trend in the weighted means and proportions over time was tested using* F* test based on weighted linear regression or Wald test based on logistic regression, with time treated as a continuous variable. Estimates for the prevalence, treatment, and control of CVRF were age-adjusted to the 2000 Census population, using the age groups of 12 to 14, 15 to 17, and 18 to 19 years. We calculated relative % change per 4-year cycle and *P* for trend using a joinpoint regression model with heteroscedastic and uncorrected errors, as previously described [26, 45]. The default maximum number of joinpoints (0 joinpoints, corresponding to a straight line) was allowed to avoid possible overfitting. The optimal fitting model was chosen by performing 4499 permutation tests based on the Monte Carlo method, adjusting for multiple tests. Parameters were estimated using weighted least squares, with weights proportional to the inverse of the variance of ln-transformed age-standardized prevalence rate at each 4-year cycle. Furthermore, logistic regression analyses were conducted, adjusting for age, sex, and race/ethnicity, to identify factors associated with the prevalence, treatment, and control of CVRFs.

To assess clinical implications of the updated guidelines for high BP on the prevalence, treatment, and control of high BP among adolescents, we also performed a sensitivity analysis by defining hypertension and elevated BP following the 2003 National Institutes of Health’s National Heart, Lung, and Blood Institute (NIH/NHLBI) and 2004 NIH/NHLBI guidelines (Additional file 1: eTable 3) [46, 47]. The definition of hypertension treatment was identical to that used in the main analysis. Hypertension was considered controlled if (1) BP was reduced to < 95th percentile in adolescents aged < 18 years or (2) BP was reduced to < 140/90 mmHg in adolescents aged 18–19 years [46, 47].

All analyses were performed using R software 4.2.3 (R Foundation) and Joinpoint Regression Program 5.0.2 (National Cancer Institute). A two-sided *P*-value of less than 0.05 was considered statistically significant.

## Results

### Baseline characteristics

There were 97,657 individuals initially identified from 2001-March 2020 NHANES. After exclusions for age < 12 or ≥ 20 years (*n* = 81,980), unavailable information on all CVRF components (*n* = 369), or pregnancy at the time of examination (*n* = 153), 15,155 adolescents aged 12–19 years were finally included, representing approximately 32.4 million noninstitutionalized and nonpregnant US population (Additional file 1: eFigure 1).

Table 1 presents the descriptive characteristics of the individuals stratified by survey periods. The mean age was 15.4 years, and 51.3% were boys. The racial and ethnic distribution was as follows: 57.4% non-Hispanic White, 14.4% non-Hispanic Black, 13.3% Mexican American, and 14.9% from other races/ethnicities; over time, the proportions of Mexican Americans and other races/ethnicities increased significantly (*P* for trend = 0.02 and < 0.001, respectively), whereas the proportion of non-Hispanic Whites decreased (*P* for trend < 0.001). The proportions of individuals who were born outside the US varied from 7.3% to 10.1%, and those who lived in poverty from 28.5% to 32.3%. The proportions of individuals with health insurance increased from 85.5% in 2001–2004 to 91.8% in 2017-March 2020 (*P* for trend < 0.001).

**Table 1:** Baseline Characteristics of US Adolescents Aged 12 to 19 Years, 2001 to March 2020^a^

Characteristics	2001–2004 (*n* = 4591)^b^	2005–2008 (*n* = 3404)^b^	2009–2012 (*n* = 2563)^b^	2013–2016 (*n* = 2693)^b^	2017-March 2020 (*n* = 1904)^b^	*P* for trend^c^
Age, mean, y	15.4 (15.3–15.5)	15.5 (15.3–15.6)	15.4 (15.3–15.5)	15.4 (15.2–15.5)	15.4 (15.3–15.5)	.83
Age group, y						
12–14	39.7 (37.2–42.1)	37.0 (34.2–39.7)	38.7 (36.3–41.0)	38.9 (36.1–41.7)	39.3 (37.2–41.4)	.77
15–17	37.0 (34.4–39.6)	39.5 (37.2–41.9)	38.5 (36.0–41.0)	39.2 (37.4–40.9)	37.4 (34.9–39.9)	.84
18–19	23.3 (20.7–26.0)	23.5 (21.3–25.6)	22.8 (20.0–25.7)	22.0 (20.1–23.8)	23.3 (21.3–25.3)	.60
Sex						
Female	48.6 (46.9–50.3)	48.5 (46.4–50.7)	48.5 (46.1–50.9)	48.8 (46.8–50.9)	49.1 (44.8–53.4)	.80
Male	51.4 (49.7–53.1)	51.5 (49.3–53.6)	51.5 (49.1–53.9)	51.2 (49.1–53.2)	50.9 (46.6–55.2)	.80
Race/ethnicity^d^						
Non-Hispanic White	63.1 (57.7–68.6)	61.9 (56.9–66.9)	56.7 (50.8–62.6)	53.3 (46.1–60.6)	50.8 (44.7–56.9)	< .001
Non-Hispanic Black	14.2 (11.1–17.3)	15.1 (11.6–18.6)	14.8 (11.0–18.6)	14.3 (10.5–18.2)	13.4 (9.3–17.5)	.71
Mexican American	10.9 (8.0–13.7)	11.5 (9.0–13.9)	13.8 (10.2–17.3)	15.1 (10.7–19.5)	15.8 (11.4–20.2)	.02
Other	11.8 (8.6–15.0)	11.6 (8.7–14.5)	14.7 (12.1–17.3)	17.3 (14.9–19.7)	20.0 (17.0–22.9)	< .001
Birth country	(*n* = 4591)	(*n* = 3403)	(*n* = 2560)	(*n* = 2692)	(*n* = 1904)	
US born	91.0 (89.2–92.8)	91.7 (89.8–93.5)	89.9 (87.9–91.9)	92.7 (91.2–94.2)	92.1 (89.8–94.3)	.31
Non-US born	9.0 (7.2–10.8)	8.3 (6.5–10.2)	10.1 (8.1–12.1)	7.3 (5.8–8.8)	7.9 (5.7–10.2)	.31
Income to poverty ratio, %	(*n* = 4318)	(*n* = 3186)	(*n* = 2316)	(*n* = 2450)	(*n* = 1680)	
< 130	31.1 (27.9–34.3)	28.5 (24.9–32.0)	32.3 (26.9–37.6)	31.0 (26.3–35.8)	29.2 (25.8–32.7)	.92
130–349	36.4 (34.1–38.6)	35.8 (32.5–39.2)	36.4 (31.9–40.9)	39.2 (35.1–43.2)	37.4 (34.0–40.7)	.25
≥ 350	32.5 (29.1–35.9)	35.7 (31.1–40.4)	31.3 (26.0–36.6)	29.8 (24.9–34.7)	33.4 (29.3–37.5)	.43
Insurance status	(*n* = 4514)	(*n* = 3377)	(*n* = 2548)	(*n* = 2683)	(*n* = 1894)	
Uninsured	14.5 (12.1–16.9)	14.5 (12.4–16.7)	12.2 (9.6–14.8)	10.2 (8.5–11.9)	8.2 (6.3–10.2)	< .001
Insured	85.5 (83.1–87.9)	85.5 (83.3–87.6)	87.8 (85.2–90.4)	89.8 (88.1–91.5)	91.8 (89.8–93.7)	< .001
Body mass index, mean, kg/m^2e^	23.3 (23.0–23.7)	23.4 (23.1–23.8)	23.8 (23.4–24.2)	24.1 (23.7–24.6)	24.4 (23.9–24.8)	< .001
Weight status^f^	(*n* = 4464)	(*n* = 3331)	(*n* = 2507)	(*n* = 2627)	(*n* = 1832)	
Normal	63.0 (60.3–65.8)	61.9 (59.4–64.4)	60.6 (57.9–63.3)	57.6 (54.7–60.5)	55.1 (51.2–59.0)	< .001
Overweight	21.0 (19.3–22.8)	21.8 (20.2–23.3)	22.1 (20.2–24.1)	23.7 (21.8–25.5)	24.8 (21.5–28.2)	.02
Obesity	15.9 (14.0–17.8)	16.3 (13.8–18.8)	17.3 (15.1–19.5)	18.7 (16.0–21.4)	20.1 (17.7–22.5)	.004
Blood pressure^g^						
Systolic	(*n* = 4371)	(*n* = 3211)	(*n* = 2438)	(*n* = 2554)	(*n* = 1653)	
Normal	85.1 (83.4–86.8)	83.6 (80.4–86.7)	85.6 (83.6–87.5)	87.1 (85.7–88.6)	85.3 (82.1–88.4)	.26
Elevated	11.2 (10.1–12.3)	11.6 (9.4–13.7)	10.7 (8.8–12.6)	10.2 (8.9–11.6)	12.2 (9.8–14.6)	.93
Stage 1	3.4 (2.5–4.2)	4.3 (3.0–5.6)	3.1 (2.2–4.0)	2.2 (1.5–2.9)	2.5 (1.2–3.8)	.02
Stage 2	0.3 (0.1–0.6)	0.5 (0.3–0.8)	0.6 (0.2–1.1)	0.4 (0.2–0.7)	0.1 (0.0–0.1)	.10
Diastolic	(*n* = 4349)	(*n* = 3202)	(*n* = 2423)	(*n* = 2539)	(*n* = 1653)	
Normal	95.2 (94.0–96.3)	96.9 (96.0–97.7)	97.9 (96.9–98.8)	98.6 (98.1–99.1)	97.0 (96.0–97.9)	< .001
Elevated	0.5 (0.2–0.8)	0.2 (0.0–0.4)	0.4 (0.0–0.8)	0.0 (0.0–0.1)	0.1 (0.0–0.2)	.01
Stage 1	3.9 (3.0–4.8)	2.7 (1.9–3.5)	1.5 (0.8–2.2)	1.2 (0.8–1.7)	2.6 (1.7–3.5)	.002
Stage 2	0.5 (0.2–0.7)	0.3 (0.0–0.5)	0.3 (0.0–0.6)	0.1 (0.0–0.2)	0.4 (0.0–1.0)	.56
Hemoglobin A_1c_, %	(*n* = 4209)	(*n* = 3015)	(*n* = 2303)	(*n* = 2375)	(*n* = 1656)	
< 5.7	97.0 (96.4–97.6)	95.9 (94.8–97.0)	93.2 (92.0–94.5)	94.0 (92.8–95.2)	93.2 (91.2–95.2)	< .001
5.7–6.4	2.4 (1.7–3.0)	3.6 (2.7–4.6)	6.3 (5.1–7.4)	5.7 (4.5–6.9)	6.2 (4.4–8.1)	< .001
≥ 6.5	0.6 (0.3–0.9)	0.5 (0.2–0.8)	0.5 (0.1–0.9)	0.3 (0.0–0.7)	0.6 (0.0–1.2)	.75
FPG, mg/dL^h^	(*n* = 2037)	(*n* = 1425)	(*n* = 1161)	(*n* = 1118)	(*n* = 740)	
< 100	88.2 (85.8–90.5)	76.2 (72.6–79.9)	83.8 (80.7–86.9)	75.0 (70.6–79.4)	65.4 (57.6–73.2)	< .001
100–125	11.3 (8.9–13.8)	23.0 (19.3–26.7)	16.0 (12.9–19.1)	24.2 (19.7–28.7)	34.2 (26.4–42.0)	< .001
≥ 126	0.5 (0.0–0.9)	0.8 (0.2–1.3)	0.2 (0.0–0.4)	0.8 (0.0–1.6)	0.4 (0.0–0.8)	.89
TC	(*n* = 4130)	(*n* = 2989)	(*n* = 2264)	(*n* = 2331)	(*n* = 1593)	
Ideal (< 170 mg/dL)	62.1 (59.6–64.5)	64.5 (61.9–67.1)	69.1 (66.6–71.6)	69.3 (67.1–71.4)	71.8 (68.3–75.3)	< .001
Intermediate (170–199 mg/dL)	25.9 (23.5–28.2)	25.3 (23.2–27.5)	22.2 (20.1–24.4)	21.6 (19.8–23.3)	22.1 (18.8–25.4)	.01
Poor (≥ 200 mg/dL)	12.1 (10.1–14.1)	10.2 (8.7–11.6)	8.6 (6.7–10.5)	9.2 (7.8–10.6)	6.1 (4.8–7.5)	< .001
HDL-C	(*n* = 4133)	(*n* = 3001)	(*n* = 2276)	(*n* = 2339)	(*n* = 1606)	
Ideal (≥ 45 mg/dL)	66.0 (64.1–68.0)	68.7 (66.5–70.9)	71.4 (69.0–73.9)	71.4 (68.6–74.2)	69.4 (65.4–73.4)	.03
Intermediate (40–44 mg/dL)	16.3 (15.2–17.4)	16.4 (14.6–18.2)	13.1 (11.4–14.9)	13.0 (11.3–14.8)	16.5 (13.7–19.3)	.24
Poor (< 40 mg/dL)	17.6 (16.0–19.3)	14.9 (13.5–16.4)	15.4 (13.6–17.2)	15.6 (13.0–18.2)	14.1 (11.8–16.4)	.06
Non-HDL-C^i^	(*n* = 4125)	(*n* = 2989)	(*n* = 2263)	(*n* = 2330)	(*n* = 1591)	
Ideal (< 120 mg/dL)	62.7 (60.8–64.7)	66.5 (63.5–69.6)	71.9 (69.2–74.6)	72.4 (70.1–74.7)	75.3 (71.7–79.0)	< .001
Intermediate (120–144 mg/dL)	23.2 (21.3–25.1)	21.1 (18.5–23.7)	17.8 (15.8–19.7)	17.6 (15.7–19.4)	16.8 (13.6–19.9)	< .001
Poor (≥ 145 mg/dL)	14.1 (12.3–15.9)	12.4 (10.6–14.1)	10.3 (8.0–12.7)	10.0 (8.5–11.6)	7.9 (6.1–9.7)	< .001
LDL-C^h^	(*n* = 1904)	(*n* = 1402)	(*n* = 1143)	(*n* = 1061)	(*n* = 715)	
Ideal (< 110 mg/dL)	75.9 (73.0–78.8)	80.0 (77.0–83.0)	80.7 (78.6–82.8)	83.7 (80.6–86.8)	83.7 (79.1–88.2)	.001
Intermediate (110–129 mg/dL)	16.2 (13.8–18.6)	13.3 (10.7–15.8)	12.0 (10.2–13.9)	10.7 (8.0–13.5)	11.5 (7.4–15.7)	.02
Poor (≥ 130 mg/dL)	7.9 (6.0–9.8)	6.7 (5.0–8.5)	7.2 (5.3–9.2)	5.6 (3.7–7.4)	4.8 (2.9–6.7)	.02
Triglycerides^h^	(*n* = 2003)	(*n* = 1406)	(*n* = 1146)	(*n* = 1062)	(*n* = 716)	
Ideal (< 90 mg/dL)	62.0 (57.9–66.0)	64.7 (61.3–68.2)	71.8 (68.3–75.2)	78.6 (74.6–82.5)	77.9 (72.1–83.6)	< .001
Intermediate (90–129 mg/dL)	20.8 (17.6–23.9)	21.1 (18.1–24.0)	17.3 (14.1–20.5)	11.7 (8.6–14.7)	15.4 (10.4–20.4)	.002
Poor (≥ 130 mg/dL)	17.3 (14.2–20.3)	14.2 (10.8–17.6)	10.9 (9.1–12.7)	9.8 (7.0–12.6)	6.7 (4.8–8.6)	< .001
Weekly exercise time, mean, min/wk^j^	185.6 (159.3–212.0)	836.3 (763.4–909.2)	993.0 (906.7–1079.4)	1044.4 (974.3–1114.6)	1617.5 (1439.2–1795.8)	< .001
HEI-2015, mean, points^k^	43.5 (42.9–44.2)	43.8 (43.0–44.6)	45.9 (45.0–46.8)	46.0 (45.2–46.8)	43.7 (42.6–44.8)	.03
Diet quality^l^	(*n* = 4357)	(*n* = 3228)	(*n* = 2417)	(*n* = 2492)	(*n* = 1740)	
Ideal	0.0 (0.0–0.1)	0.2 (0.0–0.4)	0.1 (0.0–0.3)	0.8 (0.3–1.3)	0.7 (0.1–1.3)	.03
Intermediate	23.9 (21.8–25.9)	26.4 (23.3–29.4)	32.0 (28.4–35.7)	31.7 (28.8–34.6)	27.6 (24.5–30.8)	.002
Poor	76.1 (74.0–78.1)	73.5 (70.4–76.5)	67.8 (64.1–71.5)	67.5 (64.5–70.5)	71.7 (68.6–74.9)	< .001

SI conversions: to convert glucose to mmol/L, multiply by 0.0555; TC, HDL-C, non-HDL-C, and LDL-C to mmol/L, multiply by 0.0259; triglycerides to mmol/L, multiply by 0.0113

*Abbreviations: CI* Confidence interval, *FPG* Fasting plasma glucose, *HDL-C* High-density lipoprotein cholesterol, *HEI-2015* Healthy Eating Index-2015, *LDL-C* Low-density lipoprotein cholesterol, *NHANES* National Health and Nutrition Examination Survey, *non-HDL-C* Non-high-density lipoprotein cholesterol, *TC* Total cholesterol

^a^ Nationally representative estimates of US adolescents aged 12–19 years from the 2001-March 2020 NHANES. The sample size for each 4-year interval was unweighted, whereas all other numbers were weighted means or percentages with 95% CIs

^b^ Unweighted sample size

^c^*F* test based on weighted linear regression or Wald test based on logistic regression

^d^ Race/ethnicity was based on self-report. The non-Hispanic Asian category was not available before 2011 due to the survey design, and thus estimates could not be presented separately. All other racial/ethnic groups were grouped as ‘Other’

^e^ Body mass index was missing for 394 (2.6%) participants among 15,155 included from the examination sample

^f^ Weight status (normal, overweight, and obesity) was based on Ref [38]

^g^ Blood pressure (normal, elevated, stage 1, and stage 2) were based on Ref [33, 34]

^h^ FPG, LDL-C, and triglycerides were based on fasting laboratory testing

^i^ Non-HDL-C was calculated as the difference between serum TC and HDL-C

^j^ Weekly exercise time was calculated as the minutes of moderate-intensity physical activity plus twice the minutes of vigorous-intensity physical activity per week. During 2001–2006, physical activity levels were underestimated due to a lack of data on home/yard tasks for adolescents aged 12–15 years and a lack of time data on muscle-strengthening activities across the age spectrum; data on weekly exercise time for participants aged 12–17 years were not available during 2017-March 2020, and thus estimates only represented those aged 18–19 years throughout this period. Weekly exercise time was missing for 5050 (33.3%) participants among 15,155 included from the examination sample

^k^ HEI-2015 was missing for 292 (2.0%) participants among 14,526 included from the dietary day one sample

^l^ Diet quality (ideal, intermediate, and poor) was based on Ref [42]

Compared with earlier years, individuals in the recent survey cycles were more likely to have normal diastolic BP, improved lipid profiles (including TC, HDL-C, non-HDL-C, LDL-C, and triglycerides), more exercise time, and an increased HEI-2015 score, but were prone to a higher BMI, increased HbA_1c_ level, and impaired fasting glucose. Age-adjusted trends in mean BP, HbA_1c_, FPG, TC, HDL-C, non-HDL-C, LDL-C, triglycerides levels, BMI, weekly exercise time, and HEI-2015 for all individuals and pivotal subgroups (age and race/ethnicity) are displayed in Additional file 1: eFigures 2–4 and eTable 4.

### CVRF prevalence rates

The prevalence rates of CVRFs among adolescents aged 12–19 years are summarized for age-adjusted analysis in Table 2 and for demographics-adjusted analysis in Table 3, and the secular trends are summarized in Additional file 1: eTable 4.

**Table 2:** Age-Adjusted Prevalence of Cardiovascular Risk Factors by Subgroups Among US Adolescents Aged 12 to 19 Years, 2001 to March 2020^a^

Characteristics	Adolescents with cardiovascular risk factors, % (95% CIs)^b^									
	Hypertension^c^	Elevated BP^c^	Diabetes^d^	Prediabetes^d^	Hyperlipidemia^e^	Obesity^f^	Overweight^f^	Cigarette use^g^	Inactive physical activity^h^	Poor diet quality^i^
Cases/No.^j^	901/14247	1518/14247	84/6461	1504/6461	1708/6192	2815/14761	3358/14761	1553/13865	5579/10105	10,262/14234
Age group, y										
12–14	3.4 (2.7–4.1)	7.3 (6.2–8.3)	0.9 (0.3–1.4)	28.0 (25.1–30.9)	24.3 (21.9–26.7)	16.1 (14.7–17.6)	23.7 (22.2–25.3)	2.2 (1.7–2.8)	59.8 (57.3–62.3)	70.0 (67.7–72.3)
15–17	6.7 (5.8–7.6)	11.4 (10.3–12.5)	0.8 (0.4–1.2)	21.7 (19.1–24.4)	25.4 (23.1–27.7)	17.8 (16.4–19.2)	21.5 (20.0–22.9)	13.1 (11.9–14.4)	52.9 (50.4–55.4)	72.1 (70.3–73.9)
18–19	9.4 (8.0–10.8)	14.1 (12.1–16.1)	1.4 (0.7–2.1)	22.3 (19.1–25.4)	35.1 (31.5–38.8)	19.8 (17.7–21.9)	22.7 (20.7–24.7)	24.4 (22.3–26.5)	24.7 (22.5–27.0)	71.9 (69.1–74.7)
Sex										
Female	4.0 (3.3–4.7)	5.5 (4.7–6.2)	0.9 (0.4–1.3)	16.8 (14.6–19.0)	24.0 (21.5–26.5)	18.2 (16.8–19.6)	21.8 (20.5–23.1)	10.6 (9.7–11.5)	54.8 (52.5–57.0)	69.5 (67.7–71.3)
Male	8.2 (7.3–9.1)	15.4 (14.0–16.7)	1.1 (0.6–1.6)	31.3 (28.5–34.1)	30.7 (28.2–33.1)	17.3 (16.0–18.5)	23.3 (22.0–24.7)	13.0 (11.8–14.3)	42.7 (40.6–44.8)	73.1 (71.3–74.8)
Race/ethnicity^k^										
Non-Hispanic White	6.2 (5.2–7.1)	10.3 (9.1–11.5)	0.8 (0.3–1.2)	21.9 (18.5–25.3)	28.8 (26.2–31.4)	15.5 (13.9–17.1)	22.0 (20.6–23.5)	14.3 (12.9–15.7)	46.7 (44.1–49.3)	72.9 (70.7–75.2)
Non-Hispanic Black	8.6 (7.5–9.8)	13.6 (12.5–14.7)	1.4 (0.7–2.2)	25.1 (22.0–28.2)	21.4 (19.0–23.8)	22.6 (21.0–24.3)	22.1 (20.8–23.5)	7.0 (6.1–7.9)	51.6 (48.4–54.7)	75.2 (73.1–77.3)
Mexican American	4.9 (4.0–5.8)	10.3 (8.7–11.9)	1.9 (0.8–2.9)	30.6 (26.4–34.7)	28.2 (25.3–31.1)	22.9 (21.0–24.9)	26.8 (25.3–28.3)	9.4 (8.1–10.6)	52.3 (49.7–54.9)	64.9 (62.4–67.4)
Other	4.9 (3.8–6.0)	8.6 (7.2–10.1)	0.5 (0.1–1.0)	26.0 (22.6–29.5)	27.2 (23.6–30.7)	16.6 (14.7–18.5)	21.7 (19.6–23.8)	9.4 (7.7–11.1)	47.8 (44.7–51.0)	66.7 (63.7–69.7)
Birth country										
US born	6.4 (5.7–7.1)	10.7 (9.8–11.5)	1.0 (0.7–1.4)	23.8 (21.5–26.0)	27.7 (26.0–29.4)	18.3 (17.1–19.4)	23.0 (21.9–24.0)	12.2 (11.3–13.1)	47.8 (46.1–49.5)	72.3 (70.9–73.8)
Non-US born	3.9 (2.9–4.9)	9.5 (7.0–12.0)	0.4 (0.0–0.8)	29.0 (24.0–33.9)	26.1 (21.9–30.2)	12.0 (9.8–14.2)	19.0 (16.9–21.2)	9.2 (7.3–11.1)	55.3 (51.4–59.3)	59.9 (55.7–64.1)
Income to poverty ratio, %										
< 130	6.7 (5.9–7.5)	10.3 (9.1–11.4)	1.3 (0.6–1.9)	26.2 (23.7–28.7)	29.7 (27.2–32.2)	21.5 (19.9–23.0)	24.1 (22.5–25.7)	14.9 (13.4–16.4)	49.7 (47.5–51.9)	72.9 (70.7–75.0)
130–349	6.4 (5.4–7.5)	11.3 (9.9–12.6)	0.9 (0.4–1.4)	24.7 (21.7–27.8)	28.2 (25.4–31.1)	19.1 (17.4–20.8)	23.4 (21.8–25.0)	11.8 (10.3–13.3)	48.7 (46.2–51.3)	72.8 (71.1–74.6)
≥ 350	5.0 (4.0–6.1)	9.9 (8.3–11.4)	0.8 (0.1–1.5)	20.5 (16.2–24.8)	24.9 (21.1–28.7)	12.4 (10.9–14.0)	20.5 (18.6–22.4)	9.9 (8.4–11.4)	47.2 (43.7–50.6)	69.6 (66.5–72.7)
Insurance status										
Uninsured	5.9 (4.5–7.3)	11.1 (9.0–13.2)	1.0 (0.0–2.5)	26.3 (22.0–30.7)	31.4 (26.9–35.9)	20.3 (17.5–23.1)	23.5 (21.2–25.8)	16.4 (14.2–18.5)	52.6 (48.4–56.9)	67.4 (63.9–70.9)
Insured	6.1 (5.5–6.8)	10.3 (9.5–11.2)	1.0 (0.7–1.4)	23.6 (21.4–25.8)	26.9 (25.1–28.8)	17.3 (16.2–18.4)	22.5 (21.4–23.6)	11.0 (10.2–11.8)	47.7 (45.9–49.6)	71.7 (70.2–73.2)

*Abbreviations: BP* Blood pressure, *CI* Confidence interval

^a^ Nationally representative estimates of US adolescents aged 12–19 years from the 2001-March 2020 National Health and Nutrition Examination Survey

^b^ All estimates were age-standardized to the 2000 Census population using the age groups of 12 to 14, 15 to 17, and 18 to 19 years

^c^ Hypertension was defined as stage 1 or 2 levels and/or current use of antihypertensive medications, whereas elevated BP was defined as an elevated level (see Ref. [33, 34])

^d^ Diabetes was defined as a hemoglobin A_1c_ of ≥ 6.5%, fasting plasma glucose of ≥ 126 mg/dL, self-report of previous diagnosis, and/or current use of antidiabetic medications, whereas prediabetes was defined as a hemoglobin A_1c_ of 5.7%-6.4%

^e^ Hyperlipidemia was defined as a total cholesterol of ≥ 200 mg/dL, high-density lipoprotein cholesterol of < 40 mg/dL, non-high-density lipoprotein cholesterol of ≥ 145 mg/dL, low-density lipoprotein cholesterol of ≥ 130 mg/dL, triglycerides of ≥ 130 mg/dL, and/or current use of antihyperlipidemic medications

^f^ Obesity and overweight were defined based on body mass index using the Lambda Mu Sigma method (see Ref [38])

^g^ Cigarette use was defined as smoking cigarettes within the previous 30 days

^h^ Inactive physical activity was defined as a weekly exercise time of < 420 and < 150 min/wk in adolescents aged < 18 and 18–19 years, respectively. Weekly exercise time was calculated as the minutes of moderate-intensity physical activity plus twice the minutes of vigorous-intensity physical activity per week. During 2001–2006, physical activity levels were underestimated due to a lack of data on home/yard tasks for adolescents aged 12–15 years and a lack of time data on muscle-strengthening activities across the age spectrum; data on weekly exercise time for participants aged 12–17 years were not available during 2017-March 2020, and thus estimates only represented those aged 18–19 years throughout this period

^i^ Poor diet quality was defined as a Healthy Eating Index-2015 score of < 51 points

^j^ Unweighted number of cases and sample size

^k^ Race/ethnicity was based on self-report. The non-Hispanic Asian category was not available before 2011 due to the survey design, and thus estimates could not be presented separately. All other racial/ethnic groups were grouped as ‘Other’

**Table 3:** Adjusted ORs for Cardiovascular Risk Factors by Subgroups Among US Adolescents Aged 12 to 19 Years, 2001 to March 2020^a^

Characteristics	Adolescents with cardiovascular risk factors, adjusted ORs (95% CIs)									
	Hypertension^b^	Elevated BP^b^	Diabetes^c^	Prediabetes^c^	Hyperlipidemia^d^	Obesity^e^	Overweight^e^	Cigarette use^f^	Inactive physical activity^g^	Poor diet quality^h^
Cases/No.^i^	901/14247	1518/14247	84/6461	1504/6461	1708/6192	2815/14761	3358/14761	1553/13865	5579/10105	10,262/14234
Age group, y										
12–14	1 [Reference]	1 [Reference]	1 [Reference]	1 [Reference]	1 [Reference]	1 [Reference]	1 [Reference]	1 [Reference]	1 [Reference]	1 [Reference]
15–17	2.03 (1.61, 2.56)	1.67 (1.39, 2.00)	0.96 (0.41, 2.25)	0.73 (0.61, 0.86)	1.06 (0.88, 1.28)	1.14 (1.00, 1.31)	0.88 (0.78, 1.00)	6.56 (5.04, 8.54)	0.76 (0.67, 0.86)	1.10 (0.96, 1.27)
18–19	2.90 (2.24, 3.75)	2.08 (1.66, 2.59)	1.64 (0.69, 3.89)	0.71 (0.58, 0.88)	1.67 (1.38, 2.02)	1.29 (1.11, 1.51)	0.94 (0.81, 1.09)	14.20 (10.65, 18.94)	0.22 (0.19, 0.26)	1.09 (0.92, 1.29)
Sex										
Female	1 [Reference]	1 [Reference]	1 [Reference]	1 [Reference]	1 [Reference]	1 [Reference]	1 [Reference]	1 [Reference]	1 [Reference]	1 [Reference]
Male	2.12 (1.70, 2.63)	3.18 (2.72, 3.73)	1.27 (0.64, 2.53)	2.28 (1.94, 2.69)	1.40 (1.16, 1.70)	0.94 (0.84, 1.06)	1.08 (0.97, 1.20)	1.26 (1.08, 1.47)	0.59 (0.52, 0.67)	1.20 (1.07, 1.34)
Race/ethnicity^j^										
Non-Hispanic White	1 [Reference]	1 [Reference]	1 [Reference]	1 [Reference]	1 [Reference]	1 [Reference]	1 [Reference]	1 [Reference]	1 [Reference]	1 [Reference]
Non-Hispanic Black	1.46 (1.16, 1.84)	1.40 (1.22, 1.62)	1.90 (0.88, 4.12)	1.21 (0.90, 1.62)	0.67 (0.55, 0.82)	1.60 (1.36, 1.88)	1.01 (0.91, 1.13)	0.42 (0.35, 0.51)	1.23 (1.01, 1.49)	1.13 (0.96, 1.33)
Mexican American	0.77 (0.61, 0.99)	0.99 (0.81, 1.22)	2.54 (1.12, 5.74)	1.58 (1.20, 2.09)	0.97 (0.81, 1.17)	1.63 (1.40, 1.90)	1.29 (1.16, 1.44)	0.59 (0.49, 0.73)	1.28 (1.08, 1.51)	0.69 (0.59, 0.80)
Other	0.78 (0.58, 1.05)	0.83 (0.67, 1.04)	0.70 (0.24, 2.03)	1.25 (0.94, 1.64)	0.93 (0.74, 1.17)	1.10 (0.91, 1.32)	0.98 (0.84, 1.15)	0.60 (0.46, 0.78)	1.04 (0.88, 1.23)	0.75 (0.61, 0.92)
Birth country										
US born	1 [Reference]	1 [Reference]	1 [Reference]	1 [Reference]	1 [Reference]	1 [Reference]	1 [Reference]	1 [Reference]	1 [Reference]	1 [Reference]
Non-US born	0.68 (0.50, 0.92)	0.92 (0.65, 1.28)	0.37 (0.13, 1.06)	1.15 (0.86, 1.53)	0.85 (0.67, 1.08)	0.51 (0.41, 0.64)	0.72 (0.62, 0.84)	0.87 (0.66, 1.14)	1.36 (1.12, 1.64)	0.66 (0.53, 0.81)
Income to poverty ratio, %										
< 130	1 [Reference]	1 [Reference]	1 [Reference]	1 [Reference]	1 [Reference]	1 [Reference]	1 [Reference]	1 [Reference]	1 [Reference]	1 [Reference]
130–349	0.92 (0.75, 1.14)	1.12 (0.94, 1.33)	0.76 (0.38, 1.53)	0.95 (0.78, 1.16)	0.88 (0.73, 1.05)	0.91 (0.80, 1.03)	0.97 (0.85, 1.11)	0.65 (0.52, 0.80)	1.02 (0.90, 1.15)	0.96 (0.83, 1.10)
≥ 350	0.71 (0.56, 0.91)	0.93 (0.75, 1.15)	0.74 (0.27, 2.04)	0.76 (0.58, 1.00)	0.68 (0.53, 0.87)	0.57 (0.49, 0.67)	0.83 (0.72, 0.96)	0.44 (0.34, 0.55)	1.01 (0.83, 1.23)	0.77 (0.63, 0.93)
Insurance status										
Uninsured	1 [Reference]	1 [Reference]	1 [Reference]	1 [Reference]	1 [Reference]	1 [Reference]	1 [Reference]	1 [Reference]	1 [Reference]	1 [Reference]
Insured	0.93 (0.70, 1.23)	0.90 (0.71, 1.14)	1.48 (0.42, 5.22)	0.85 (0.67, 1.07)	0.81 (0.63, 1.04)	0.90 (0.75, 1.08)	1.00 (0.87, 1.16)	0.51 (0.42, 0.61)	0.84 (0.68, 1.03)	1.10 (0.93, 1.31)

*Abbreviations: BP* Blood pressure, *CI* Confidence interval, *OR* Odds ratio

^a^ Nationally representative estimates of US adolescents aged 12–19 years from the 2001-March 2020 National Health and Nutrition Examination Survey. Adjusted ORs with 95% CIs were adjusted for age, sex, and race/ethnicity groups

^b^ Hypertension was defined as stage 1 or 2 levels and/or current use of antihypertensive medications, whereas elevated BP was defined as an elevated level (see Ref [33, 34])

^c^ Diabetes was defined as a hemoglobin A_1c_ of ≥ 6.5%, fasting plasma glucose of ≥ 126 mg/dL, self-report of previous diagnosis, and/or current use of antidiabetic medications, whereas prediabetes was defined as a hemoglobin A_1c_ of 5.7%-6.4%

^d^ Hyperlipidemia was defined as a total cholesterol of ≥ 200 mg/dL, high-density lipoprotein cholesterol of < 40 mg/dL, non-high-density lipoprotein cholesterol of ≥ 145 mg/dL, low-density lipoprotein cholesterol of ≥ 130 mg/dL, triglycerides of ≥ 130 mg/dL, and/or current use of antihyperlipidemic medications

^e^ Obesity and overweight were defined based on body mass index using the Lambda Mu Sigma method (see Ref [38])

^f^ Cigarette use was defined as smoking cigarettes within the previous 30 days

^g^ Inactive physical activity was defined as a weekly exercise time of < 420 and < 150 min/wk in adolescents aged < 18 and 18–19 years, respectively. Weekly exercise time was calculated as the minutes of moderate-intensity physical activity plus twice the minutes of vigorous-intensity physical activity per week. During 2001–2006, physical activity levels were underestimated due to a lack of data on home/yard tasks for adolescents aged 12–15 years and a lack of time data on muscle-strengthening activities across the age spectrum; data on weekly exercise time for participants aged 12–17 years were not available during 2017-March 2020, and thus estimates only represented those aged 18–19 years throughout this period

^h^ Poor diet quality was defined as a Healthy Eating Index-2015 score of < 51 points

^i^ Unweighted number of cases and sample size

^j^ Race/ethnicity was based on self-report. The non-Hispanic Asian category was not available before 2011 due to the survey design, and thus estimates could not be presented separately. All other racial/ethnic groups were grouped as ‘Other’

**Fig. 1:**
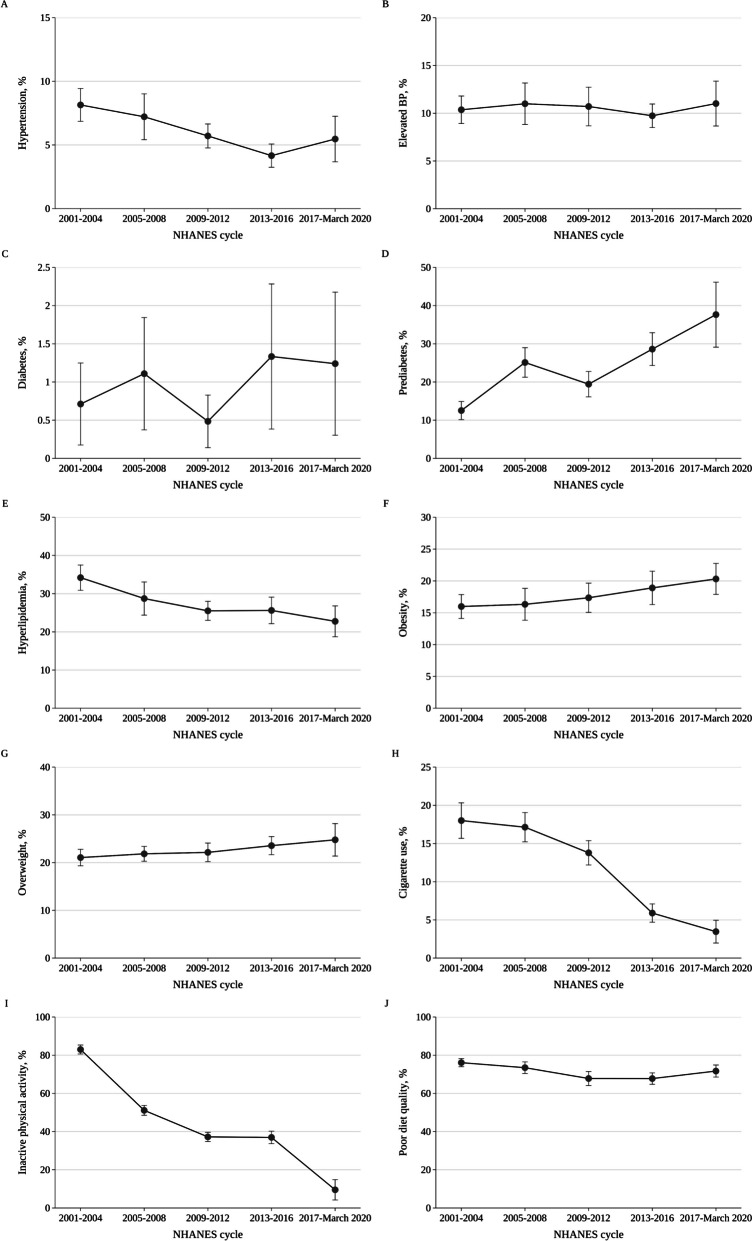
Age-Adjusted Trends in the Prevalence of Hypertension, Elevated BP, Diabetes, Prediabetes, Hyperlipidemia, Obesity, Overweight, Cigarette Use, Inactive Physical Activity, and Poor Diet Quality Among US Adolescents Aged 12 to 19 Years, 2001 to March 2020^a−i^. *Abbreviations*: *BP* Blood pressure, *CI *Confidence interval.^a^ Nationally representative estimates of US adolescents aged 12–19 years from the 2001-March 2020 National Health and Nutrition Examination Survey. Whiskers indicate 95% CIs. *P* for trend was calculated by the Joinpoint Regression Program: *P* = .04 for hypertension in panel **A**; *P* = .73 for elevated BP in panel **B**; *P* = .46 for diabetes in panel **C**; *P* = .08 for prediabetes in panel **D**; *P* = .01 for hyperlipidemia in panel **E**; *P* = .002 for obesity in panel **F**; *P* = .004 for overweight in panel **G**; *P* = .07 for cigarette use in panel **H**; *P* = .01 for inactive physical activity in panel **I**; and *P* = .13 for poor diet quality in panel **J**. Specific estimates are presented in Additional file 1: eTable 4. ^b^ All estimates were age-standardized to the 2000 Census population using the age groups of 12 to 14, 15 to 17, and 18 to 19 years. ^c^ Hypertension was defined as stage 1 or 2 levels and/or current use of antihypertensive medications, whereas elevated BP was defined as an elevated level (see Ref [33, 34]). ^d^ Diabetes was defined as a hemoglobin A_1c_ of ≥ 6.5%, fasting plasma glucose of ≥ 126 mg/dL, self-report of previous diagnosis, and/or current use of antidiabetic medications, whereas prediabetes was defined as a hemoglobin A_1c_ of 5.7%-6.4%.^e^ Hyperlipidemia was defined as a total cholesterol of ≥ 200 mg/dL, high-density lipoprotein cholesterol of < 40 mg/dL, non-high-density lipoprotein cholesterol of ≥ 145 mg/dL, low-density lipoprotein cholesterol of ≥ 130 mg/dL, triglycerides of ≥ 130 mg/dL, and/or current use of antihyperlipidemic medications. ^f^ Obesity and overweight were defined based on body mass index using the Lambda Mu Sigma method (see Ref. [38]). ^g^ Cigarette use was defined as smoking cigarettes within the previous 30 days. ^h^ Inactive physical activity was defined as a weekly exercise time of < 420 and < 150 min/wk in adolescents aged < 18 and 18–19 years, respectively. Weekly exercise time was calculated as the minutes of moderate-intensity physical activity plus twice the minutes of vigorous-intensity physical activity per week. During 2001–2006, physical activity levels were underestimated due to a lack of data on home/yard tasks for adolescents aged 12–15 years and a lack of time data on muscle-strengthening activities across the age spectrum; data on weekly exercise time for participants aged 12–17 years were not available during 2017-March 2020, and thus estimates only represented those aged 18–19 years throughout this period. ^i^ Poor diet quality was defined as a Healthy Eating Index-2015 score of < 51 points

The age-adjusted prevalence of hypertension among adolescents aged 12–19 years significantly decreased from 8.1% (95% CI, 6.9%-9.4%) in 2001–2004 to 5.5% (95% CI, 3.7%-7.3%) in 2017-March 2020 (Fig. 1A), with a -15.3% relative decrease (95% CI, -26.8% to -1.9%) per 4-year cycle (*P* for trend = 0.04). The age-adjusted prevalence of elevated BP and diabetes did not change over this period (*P* for trend = 0.73 and 0.27, respectively) (Figs. 1B-C). The age-adjusted prevalence of prediabetes was numerically higher in 2017-March 2020 than in 2001–2004 (37.6% [95% CI, 29.1%-46.2%] versus 12.5% [95% CI, 10.2%-14.9%], Fig. 1D), although the difference did not reach significance (*P* for trend = 0.08). The age-adjusted prevalence of hyperlipidemia was lower in 2017-March 2020 (22.8% [95% CI, 18.7%-26.8%]) than in 2001–2004 (34.2% [95% CI, 30.9%-37.5%]) (Fig. 1E), with a -9.8% relative decrease (95% CI, -15.2% to -4.0%) per 4-year cycle (*P* for trend = 0.01).

**Fig. 2:**
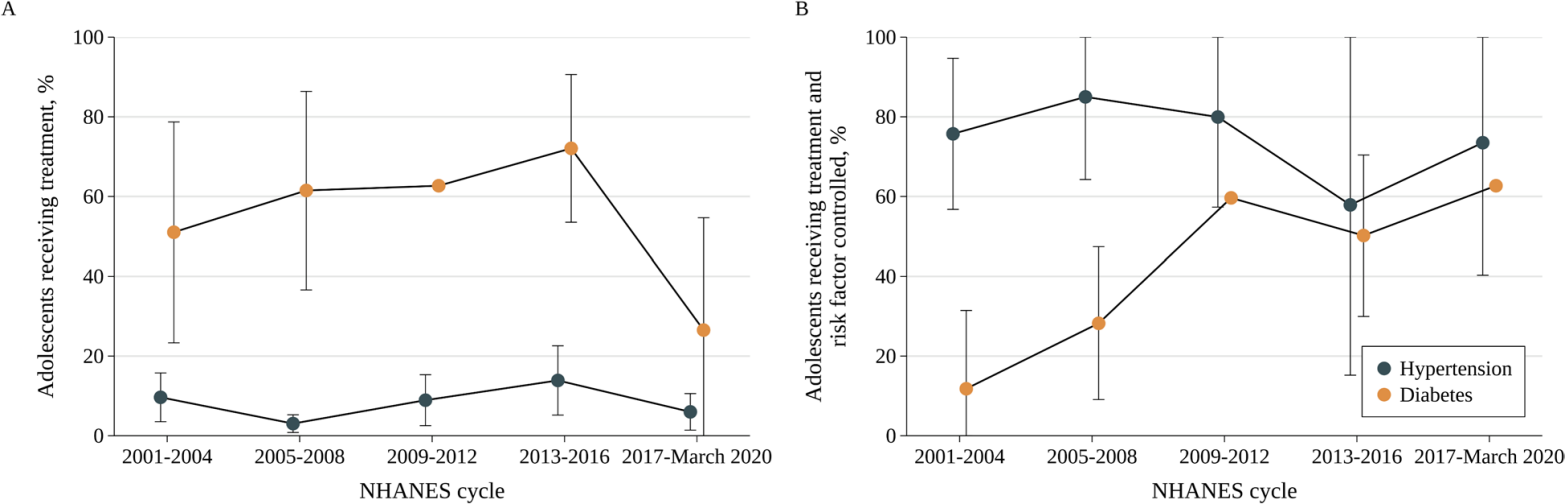
Age-Adjusted Trends in the Rates of Hypertension and Diabetes Treatment and Control Among US Adolescents Aged 12 to 19 Years, 2001 to March 2020^a−d^. *Abbreviations*: *BP* Blood pressure, *CI* Confidence interval. ^a^ Nationally representative estimates of US adolescents aged 12–19 years from the 2001-March 2020 National Health and Nutrition Examination Survey. Whiskers indicate 95% CIs. *P* for trend was calculated by the Joinpoint Regression Program: *P* = .79 for hypertension treatment and *P* = .60 for diabetes treatment in panel **A**; *P* = .66 for BP control and *P* value was not applicable for glycemic control in panel **B**. Specific estimates are presented in Additional file 1: eTable 4. ^b^ All estimates were age-standardized to the 2000 Census population using the age groups of 12 to 14, 15 to 17, and 18 to 19 years. ^c^ Hypertension treatment was defined as current use of antihypertensive medications and was evaluated among adolescents with hypertension (n = 901). Diabetes treatment was defined as current use of antidiabetic medications and was evaluated among adolescents with diabetes (*n* = 84). ^d^ Control was evaluated among adolescents receiving treatment (*n* = 68 for hypertension and *n* = 40 for diabetes). Hypertension was considered controlled if (1) BP was reduced to < 90th percentile in adolescents aged < 13 years, (2) BP was reduced to < 90th percentile and < 130/80 mmHg in adolescents aged 13–17 years, or (3) BP was reduced to < 130/80 mmHg in adolescents aged 18–19 years. Diabetes was considered controlled if hemoglobin A_1c_ was reduced to < 7%

The age-adjusted prevalence of obesity significantly increased from 16.0% (95% CI, 14.1%-17.9%) in 2001–2004 to 20.3% (95% CI, 17.9%-22.7%) in 2017-March 2020 (Fig. 1F), with a 6.4% relative increase (95% CI, 4.2%-8.6%) per 4-year cycle (*P* for trend = 0.002); as did overweight (from 21.1% [95% CI, 19.3%-22.8%] in 2001–2004 to 24.8% [95% CI, 21.4%-28.2%] in 2017-March 2020) (Fig. 1G), with a 3.9% relative increase (95% CI, 2.3%-5.5%) per 4-year cycle (*P* for trend = 0.004). The age-adjusted prevalence of cigarette use was numerically lower in 2017-March 2020 than in 2001–2004 (3.5% [95% CI, 2.0%-5.0%] versus 18.0% [95% CI, 15.7%-20.3%], Fig. 1H), although the difference was not statistically significant (*P* for trend = 0.07). The age-adjusted prevalence of inactive physical activity was lower in 2017-March 2020 (9.5% [95% CI, 4.2%-14.8%]) than in 2001–2004 (83.0% [95% CI, 80.7%-85.3%]) (Fig. 1I), with a -29.0% relative decrease (95% CI, -41.0% to -14.7%) per 4-year cycle (*P* for trend = 0.01). There was no significant change in the age-adjusted prevalence of poor diet quality (*P* for trend = 0.13) (Fig. 1J).

The adjusted prevalence of hypertension or elevated BP was higher among older individuals, boys, and non-Hispanic Blacks; individuals who were Mexican American, who were born outside the US, or who were from high-income families were less likely to have hypertension. Mexican Americans were more likely to have both diabetes and prediabetes; the adjusted prevalence of prediabetes was also higher among boys, while being lower among older individuals. Hyperlipidemia was more likely among older individuals and boys, and was less likely among non-Hispanic Blacks and affluent individuals. Obesity and overweight were more common among Mexican Americans, while being less common among non-US-born or affluent individuals; older individuals and non-Hispanic Blacks were also more likely to have obesity. The adjusted prevalence of cigarette use correlated positively with being older and male, while it correlated negatively with being non-Hispanic Black, Mexican American, or the other racial/ethnic group, coming from middle- or high-income families, and having health insurance. Inactive physical activity was more likely among non-Hispanic Blacks or Mexican Americans and non-US-born individuals, and was less likely among older individuals and boys. The adjusted prevalence of poor diet quality was higher among boys, while it was lower among individuals who were Mexican American or the other racial/ethnic group, who were born outside the US, or who were from high-income families.

Age-adjusted trends in CVRF prevalence rates were generally similar between boys and girls, whereas there were some disparities across races/ethnicities (Additional file 1: eFigures 5–6). Non-Hispanic Whites experienced a significant decrease in hyperlipidemia (2001–2004: 36.6% [95% CI, 31.9%-41.4%]; 2017-March 2020: 21.3% [95% CI, 13.7%-28.8%]; *P* for trend = 0.02), whereas an increase in diabetes was observed among non-Hispanic Blacks (2001–2004: 0.6% [95% CI, 0.0%-1.3%]; 2017-March 2020: 3.6% [95% CI, 0.8%-6.4%]; *P* for trend = 0.005). Mexican Americans experienced a significant decrease in hyperlipidemia (2001–2004: 32.4% [95% CI, 29.1%-35.6%]; 2017-March 2020: 25.7% [95% CI, 17.9%-33.5%]; *P* for trend < 0.001), but also an increase in obesity (2001–2004: 16.7% [95% CI, 14.3%-19.0%]; 2017-March 2020: 29.4% [95% CI, 22.6%-36.1%]; *P* for trend = 0.009) and overweight (2001–2004: 24.0% [95% CI, 21.6%-26.5%]; 2017-March 2020: 28.8% [95% CI, 24.5%-33.2%]; *P* for trend = 0.04), whereas a decrease in hypertension was observed in the other racial/ethnic group (2001–2004: 6.3% [95% CI, 2.9%-9.7%]; 2017-March 2020: 3.6% [95% CI, 1.7%-5.4%]; *P* for trend = 0.03). Trends in the prevalence of elevated BP, prediabetes, cigarette use, inactive physical activity, and poor diet quality were generally comparable across racial/ethnic groups over the study period.

### CVRF treatment and control rates

Among adolescents aged 12–19 years with hypertension, the use of any antihypertensive medication did not change significantly, from 9.6% (95% CI, 3.5%-15.8%) in 2001–2004 to 6.0% (95% CI, 1.4%-10.6%) in 2017-March 2020 (*P* for trend = 0.79) (Fig. 2A and Additional file 1: eTable 4). Among those receiving pharmacologic therapy, there was also no significant change in age-adjusted hypertension control rates, from 75.7% (95% CI, 56.8%-94.7%) in 2001–2004 to 73.5% (95% CI, 40.3%-100.0%) in 2017-March 2020 (*P* for trend = 0.66). Hypertension treatment was generally more likely among older individuals, and was less likely among boys (Additional file 1: eTables 5–6). There was no significant difference in BP control across different sociodemographic subpopulations after adjusting for other factors.

Among adolescents with diabetes, the use of any antidiabetic medication did not change significantly, from 51.0% (95% CI, 23.3%-78.7%) in 2001–2004 to 26.5% (95% CI, 0.0%-54.7%) in 2017-March 2020 (*P* for trend = 0.60) (Fig. 2B and Additional File 1: eTable 4). Among those receiving pharmacologic therapy, higher age-adjusted diabetes control rates were observed in 2017-March 2020 (62.7% [95% CI, 62.7%-62.7%]) than in 2001–2004 (11.8% [95% CI, 0.0%-31.5%]), although the difference did not attain significance (*P* value was not applicable). No significant difference was seen in diabetes treatment across different sociodemographic subpopulations after adjusting for other factors (Additional file 1: eTables 5–6). Compared with younger individuals, older individuals were more likely to achieve individualized HbA_1c_ targets.

Treatment and control rates for hypertension and diabetes by age and race/ethnicity were not evaluated due to limited sample size.

### Sensitivity analysis

When using the 2003 NIH/NHLBI and 2004 NIH/NHLBI guidelines, the age-adjusted prevalence of hypertension decreased (2001–2004: 4.8% [95% CI, 3.8%-5.7%]; 2017-March 2020: 3.0% [95% CI, 1.8%-4.1%]; *P* for trend = 0.04), whereas the age-adjusted prevalence of elevated BP increased (2001–2004: 14.8% [95% CI, 13.1%-16.6%]; 2017-March 2020: 14.6% [95% CI, 11.7%-17.5%]; *P* for trend = 0.27), although trends over time were similar (Additional file 1: eFigure 7 and eTable 4). The use of any antihypertensive medication was substantially higher among adolescents with hypertension based on older guidelines (2001–2004: 19.3% [95% CI, 10.3%-28.4%]; 2017-March 2020: 28.3% [95% CI, 21.4%-35.1%]; *P* for trend = 0.17), as was hypertension control rates (2001–2004: 90.5% [95% CI, 75.9%-100.0%]; 2017-March 2020: 82.8% [95% CI, 57.1%-100.0%]; *P* value was not applicable).

## Discussion

Between 2001 and March 2020, prediabetes and overweight/obesity increased among US adolescents aged 12–19 years, while hypertension, hyperlipidemia, cigarette use, and inactive physical activity decreased, and elevated BP, diabetes, and poor diet quality did not change markedly. Boys experienced a higher prevalence of hypertension, elevated BP, prediabetes, hyperlipidemia, cigarette use, and poor diet quality, and a lower prevalence of inactive physical activity than girls. Furthermore, among racial/ethnic groups, non-Hispanic Whites had a significant decrease in hyperlipidemia, while an increase in diabetes was seen among non-Hispanic Blacks. Mexican Americans had a significant decrease in hyperlipidemia, but also an increase in overweight/obesity, while a decrease in hypertension was seen in the other racial/ethnic group. Trends in the prevalence of elevated BP, prediabetes, cigarette use, inactive physical activity, and poor diet quality were generally similar across racial/ethnic groups. Whilst treatment rates for hypertension and diabetes did not show improvement over time, BP control remained relatively stable, and there were some improvements in glycemic control, although it remained suboptimal.

The increase in overweight/obesity prevalence (to 24.8% and 20.3%, respectively) among adolescents was consistent with that previously observed [48–51]. This rise in overweight/obesity rates may have a negative impact on public health and healthcare budgets because obesity is strongly correlated with other CVRFs such as hypertension, diabetes, and hyperlipidemia, which could result in premature CVDs and cardiovascular death during adulthood [13, 16, 52–54]. Several factors may contribute to the increase in overweight/obesity prevalence, including larger food portion sizes, consumption of energy-dense, nutrient-poor foods, changing modes of transportation, and sedentary behaviors [55–58]. The prevalence of prediabetes increased from 12.5% to 37.6% during this period, although the difference did not reach significance, whereas there was no noticeable change over time in the prevalence of diabetes. Additionally, the prevalence of poor diet quality decreased from 76.1% to 67.8% from 2001–2004 to 2009–2012 but then increased to 71.7% in 2017-March 2020. Multicomponent behavior-changing interventions (e.g., increased physical activity [regardless of light or moderate-to-vigorous intensity], reduced sedentary behavior, adequate sleep, and high-quality diets) [59–63], in combination with improved parent support behaviors [64], could help ease the burden of overweight/obesity for this population.

Despite the increased overweight/obesity prevalence, there was a gradual decrease in the prevalence of hypertension, from 8.1% to 5.5%, with similar results seen in a sensitivity analysis using the older guidelines (from 4.8% to 3.0%). This decrease was consistent with that seen previous reports [65, 66]. Actually, obesity appears to be more relevant to wide pulse pressure rather than mean arterial pressure [67]. The contrary trends may be partly explained by reduced lead exposure acting on small resistance arteries, improved screening programs, and earlier pharmacologic or lifestyle interventions such as increased physical activity, smoking cessation, and sodium restriction [68–72]. The prevalence of inactive physical activity significantly decreased from 83.0% to 9.5%, as did the prevalence of cigarette use (from 18.0% to 3.5%, although the difference was not statistically significant). Hyperlipidemia also showed a relative decrease of -33.3% over the past two decades, possibly due to reduced levels of trans-fatty acids in the food supply and a decline in smoking prevalence [73, 74]. These findings suggest that interventions targeting hypertension, hyperlipidemia, cigarette use, and physical activity have been more effective in comparison to interventions targeting overweight/obesity, prediabetes/diabetes, and diet quality. In light of the strong association between CVRFs and target organ damage during adolescence, as well as premature cardiovascular morbidity and mortality during adulthood, our data support the need for strengthening public health planning and individualized clinical interventions [5, 11–19].

Considerable disparities exist regarding the prevalence rates and trends of CVRFs across different sex and racial/ethnic groups. On the one hand, boys were consistently at higher risk for hypertension, elevated BP, prediabetes, hyperlipidemia, cigarette use, and poor diet quality; however, on the other hand, boys were at lower risk for inactive physical activity. Men presented a similar risk for the aforementioned CVRFs compared to women as boys were to girls, and were more likely to experience severe adverse events (e.g., myocardial infarction, stroke, heart failure, and cardiovascular death) [75–77]. The exact mechanisms underlying such inequalities are not fully understood, but are likely influenced by differences in inherent physiology, anti-inflammatory immune profiles, sex steroid hormone levels, and lifestyles (e.g., sodium intake) [78, 79]. Thus, it is necessary to adopt more effective strategies that consider sex differences in the management of CVRFs.

Unlike other races/ethnicities, non-Hispanic Blacks experienced a significant increase in diabetes, which may partly be attributed to early-life exposures, education, availability of services, and other factors potentially related to structural racism [80]. Planning future efforts to address and mitigate these inequalities across racial/ethnic groups should be prioritized in the management of CVRFs among US adolescents. Besides, a clinically relevant increase in overweight/obesity prevalence was observed in Mexican Americans during the study period, which was generally consistent with previous reports [48, 49]. While disparities in overweight/obesity between Mexican Americans and non-Hispanic Whites used to be specific to US-born Mexican Americans, the disparities have expanded to non-US-born Mexican Americans over recent years [81]. Possible reasons include the recent and rapid nutrition transition, changing selection migration dynamics, and longer time to live in the US [82–84]. However, in contrast to the increase in obesity, Mexican Americans, together with non-Hispanic Whites, had a significant decrease in hyperlipidemia. Further studies are needed to explore the contributing factors for the fluctuation.

Throughout the survey period, hypertension treatment rate remained low (< 15%), albeit this may be partly explained by earlier lifestyle modifications. In a retrospective study including 15,422 children (aged 3–17 years) with BP equal to or greater than 95th percentile, 14,841 (96.2%) children sought lifestyle counseling, whereas 831 (5.4%) children received antihypertensive medications, and 848 (5.5%) children received BP-related referrals [85]. Approximately 75% of adolescents receiving antihypertensive medications achieved BP targets at both the start and end of study period. When using the older guidelines, the proportions of adolescents who received antihypertensive medications or who achieved BP targets were substantially higher (varying between 8.0%-28.3% and 79.9%-100.0%, respectively). Meanwhile, diabetes treatment rates ranged from 26.5% to 72.1%, with no significant difference found during the study period. There was an upward trend in glycemic control rates (from 11.8% to 62.7%), although the difference was not statistically significant. A consistent screening, treatment, and monitoring program for adolescents is curial to ensure that they are receiving the best care available.

## Limitations

This study has several limitations. First, misclassification of elevated BP/hypertension, prediabetes/diabetes, and hyperlipidemia may have existed due to the use of self-reported diagnoses and dependence on single-occasion physical examination or laboratory testing, possibly leading to an overestimation of CVRF prevalence among adolescents. Second, recommendations for the definition of hypertension and target BP levels have changed over the entire study period, resulting in a higher prevalence of hypertension and lower treatment and control rates. Third, we assessed risk factor treatment and control relying only on medication use, without considering lifestyle modifications such as salt-reduced diets and aerobic exercise, which are usually taken prior to pharmacologic therapy. Fourth, during 2001–2006, physical activity levels were underestimated due to a lack of data on home/yard tasks for adolescents aged 12–15 years and a lack of time data on muscle-strengthening activities across the age spectrum. Additionally, because data on weekly exercise time for participants aged 12–17 years were not available during 2017-March 2020, estimates only represented those aged 18–19 years throughout this period, potentially underestimating the prevalence of inactive physical inactivity. Fifth, the response rates for the NHANES have declined over time. Sixth, although the combination of two continuous NHANES cycles improved the reliability of prevalence estimates, the study may not have had sufficient statistical power to detect small changes in population subgroups with limited sample sizes. Finally, a proportion of nonpregnant participants (*n* = 368) were excluded because of insufficient clinical information. As the sample differed slightly from the included population and between survey years in terms of baseline characteristics, this study may not have been completely free of selection bias (Additional file 1: eTables 7–8). However, the proportion of missing data was low (≈ 2.4%); therefore, exclusion of the sample were not expected to significantly affect the results.

## Conclusions

Over the past 2 decades, despite an increase in prediabetes and overweight/obesity, hypertension, hyperlipidemia, cigarette use, and inactive physical activity decreased among US adolescents aged 12 to 19 years, while elevated BP, diabetes, and poor diet quality remained unchanged. There were disparities in the prevalence of and trends in CVRFs across sociodemographic subpopulations. While treatment rates for hypertension and diabetes did not improve over time, BP control remained relatively stable, and there were numerical improvements in glycemic control.

### Supplementary Information

**Additional file 1: eMethod 1**. Therapeutic Drug Classes Used to Define Any Use of Antihypertensive and Antidiabetic Medications. **eFigure 1**. Inclusion Diagram for US Adolescents Aged 12 to 19 Years, 2001 to March 2020. **eFigure 2**. Age-Adjusted Trends in Mean BP, Hemoglobin A_1c_, FPG, TC, HDL-C, Non-HDL-C, LDL-C, Triglycerides Levels, Body Mass Index, Weekly Exercise Time, and HEI-2015 for US Adolescents Aged 12 to 19 Years, 2001 to March 2020. **eFigure 3**. Age-Adjusted Trends in Mean BP, Hemoglobin A_1c_, FPG, TC, HDL-C, Non-HDL-C, LDL-C, Triglycerides Levels, Body Mass Index, Weekly Exercise Time, and HEI-2015 for US Adolescents Aged 12 to 19 Years by Sex, 2001 to March 2020. **eFigure 4**. Age-Adjusted Trends in Mean BP, Hemoglobin A_1c_, FPG, TC, HDL-C, Non-HDL-C, LDL-C, Triglycerides Levels, Body Mass Index, Weekly Exercise Time, and HEI-2015 for US Adolescents Aged 12 to 19 Years by Race/Ethnicity, 2001 to March 2020. **eFigure 5**. Age-Adjusted Trends in the Prevalence of Hypertension, Elevated BP, Diabetes, Prediabetes, Hyperlipidemia, Obesity, Overweight, Cigarette Use, Inactive Physical Activity, and Poor Diet Quality Among US Adolescents Aged 12 to 19 Years by Sex, 2001 to March 2020. **eFigure 6**. Age-Adjusted Trends in the Prevalence of Hypertension, Elevated BP, Diabetes, Prediabetes, Hyperlipidemia, Obesity, Overweight, Cigarette Use, Inactive Physical Activity, and Poor Diet Quality Among US Adolescents Aged 12 to 19 Years by Race/Ethnicity, 2001 to March 2020. Years by Race/Ethnicity, 2001 to March 2020. **eFigure 7**. Age-Adjusted Trends in the Prevalence of High BP and Hypertension Treatment and Control Rates Among US Adolescents Aged 12 to 19 Years According to the 2003 NIH/NHLBI and 2004 NIH/NHLBI Guidelines, 2001 to March 2020. **eTable 1**. Unweighted Response Rates for the NHANES In-Home Interviews and Mobile Examinations Among US Adolescents Aged 12 to 19 Years by Age and Sex Groups, 2001 to March 2020. **eTable 2**. Strengthening the Reporting of Observational Studies in Epidemiology (STROBE) Reporting Guideline for Reporting Cross-sectional Studies Checklist. **eTable 3**. Classification of BP by the 2003 NIH/NHLBI, 2004 NIH/NHLBI, 2017 AAP, and 2017 ACC/AHA Guidelines. **eTable 4**. Trends in Age-Adjusted Means or % (95% CIs) of Cardiovascular Parameters, Cardiovascular Risk Factors, and Hypertension and Diabetes Treatment and Control Among US Adolescents Aged 12 to 19 Years by Age and Racial/Ethnic Groups, 2001 to March 2020. **eTable 5**. Age-Adjusted Rates of Hypertension and Diabetes Treatment and Control by Subgroups Among US Adolescents Aged 12 to 19 Years, 2001 to March 2020. **eTable 6**. Adjusted ORs for Hypertension and Diabetes Treatment and Control by Subgroups Among US Adolescents Aged 12 to 19 Years, 2001 to March 2020. **eTable 7**. Comparison of Baseline Characteristics Between the Included and Excluded Study Population. **eTable 8**. Baseline Characteristics of the Excluded Study Population, 2001 to March 2020.

## Data Availability

All raw data included in this study are publicly available at https://wwwn.cdc.gov/nchs/nhanes/.

## Abbreviations

BMI: Body mass index
BP: Blood pressure
CDC: Centers for Disease Control and Prevention
CI: Confidence interval
CVD: Cardiovascular disease
CVRF: Cardiovascular risk factor
FPG: Fasting plasma glucose
HbA_1c_: Hemoglobin A_1c_
HDL-C: High-density lipoprotein cholesterol
HEI-2015: Healthy Eating Index-2015
IPR: Income to poverty ratio
LDL-C: Low-density lipoprotein cholesterol
NCHS: National Center for Health Statistics
NHANES: National Health and Nutrition Examination Survey
NIH/NHLBI: National Institutes of Health’s National Heart, Lung, and Blood Institute
non-HDL-C: Non-high-density lipoprotein cholesterol
STROBE: Strengthening the Reporting of Observational Studies in Epidemiology
TC: Total cholesterol

## References

[1] Roth GA, Mensah GA, Johnson CO, Addolorato G, Ammirati E, Baddour LM (2020). Global burden of cardiovascular diseases and risk factors, 1990–2019: update from the GBD 2019 study. J Am Coll Cardiol.

[2] Tsao CW, Aday AW, Almarzooq ZI, Anderson CAM, Arora P, Avery CL (2023). Heart disease and stroke statistics-2023 update: a report from the American Heart Association. Circulation.

[3] Agency for Healthcare Research and Quality. Medical Expenditure Panel Survey (MEPS): household component summary tables: medical conditions, United States. https://meps.ahrq.gov/mepsweb/. Accessed 10 Jul 2023.

[4] Ruiz JR, Cavero-Redondo I, Ortega FB, Welk GJ, Andersen LB, Martinez-Vizcaino V (2016). Cardiorespiratory fitness cut points to avoid cardiovascular disease risk in children and adolescents; what level of fitness should raise a red flag? A systematic review and meta-analysis. Br J Sports Med.

[5] Price JJ, Urbina EM, Carlin K, Becker R, Daniels SR, Falkner BE (2022). Cardiovascular risk factors and target organ damage in adolescents: the SHIP AHOY study. Pediatrics.

[6] Agbaje AO (2023). Elevated blood pressure and worsening cardiac damage during adolescence. J Pediatr.

[7] Meng Y, Sharman JE, Koskinen JS, Juonala M, Viikari JSA, Buscot MJ (2024). Blood pressure at different life stages over the early life course and intima-media thickness. JAMA Pediatr.

[8] Agbaje AO (2024). Increasing lipids with risk of worsening cardiac damage in 1595 adolescents: a 7-year longitudinal and mediation study. Atherosclerosis..

[9] Agbaje AO, Saner C, Zhang J, Henderson M, Tuomainen TP. DEXA-based fat mass with the risk of worsening insulin resistance in adolescents: a 9-year temporal and mediation study. J Clin Endocrinol Metab. 2024. 10.1210/clinem/dgae004. Online ahead of print. 10.1210/clinem/dgae004 PMC11319001 38173399

[10] Stanesby O, Armstrong MK, Otahal P, Goode JP, Fraser BJ, Negishi K (2024). Tracking of serum lipid levels from childhood to adulthood: systematic review and meta-analysis. Atherosclerosis.

[11] Pool LR, Aguayo L, Brzezinski M, Perak AM, Davis MM, Greenland P (2021). Childhood risk factors and adulthood cardiovascular disease: a systematic review. J Pediatr.

[12] Jacobs DR, Woo JG, Sinaiko AR, Daniels SR, Ikonen J, Juonala M (2022). Childhood cardiovascular risk factors and adult cardiovascular events. N Engl J Med.

[13] Yang L, Magnussen CG, Yang L, Bovet P, Xi B (2020). Elevated blood pressure in childhood or adolescence and cardiovascular outcomes in adulthood: a systematic review. Hypertension.

[14] Rawshani A, Sattar N, Franzén S, Rawshani A, Hattersley AT, Svensson AM (2018). Excess mortality and cardiovascular disease in young adults with type 1 diabetes in relation to age at onset: a nationwide, register-based cohort study. Lancet.

[15] Mainieri F, La Bella S, Chiarelli F (2023). Hyperlipidemia and cardiovascular risk in children and adolescents. Biomedicines.

[16] Twig G, Yaniv G, Levine H, Leiba A, Goldberger N, Derazne E (2016). Body-mass index in 2.3 million adolescents and cardiovascular death in adulthood. N Engl J Med.

[17] Thomson B, Emberson J, Lacey B, Peto R, Woodward M, Lewington S (2020). Childhood smoking, adult cessation, and cardiovascular mortality: prospective study of 390 000 US adults. J Am Heart Assoc.

[18] Scott JM, Li N, Liu Q, Yasui Y, Leisenring W, Nathan PC (2018). Association of exercise with mortality in adult survivors of childhood cancer. JAMA Oncol.

[19] Ness AR, Maynard M, Frankel S, Smith GD, Frobisher C, Leary SD (2005). Diet in childhood and adult cardiovascular and all cause mortality: the Boyd Orr cohort. Heart.

[20] McCrindle BW, Manlhiot C, Millar K, Gibson D, Stearne K, Kilty H (2010). Population trends toward increasing cardiovascular risk factors in Canadian adolescents. J Pediatr.

[21] Shay CM, Ning H, Daniels SR, Rooks CR, Gidding SS, Lloyd-Jones DM (2013). Status of cardiovascular health in US adolescents: prevalence estimates from the National Health and Nutrition Examination Surveys (NHANES) 2005–2010. Circulation.

[22] Ning H, Labarthe DR, Shay CM, Daniels SR, Hou L, Van Horn L, Lloyd-Jones DM (2015). Status of cardiovascular health in US children up to 11 years of age: the National Health and Nutrition Examination Surveys 2003–2010. Circ Cardiovasc Qual Outcomes.

[23] Henriksson P, Henriksson H, Gracia-Marco L, Labayen I, Ortega FB, Huybrechts I (2017). Prevalence of ideal cardiovascular health in European adolescents: the HELENA study. Int J Cardiol.

[24] Vazeou A, Tittel SR, Kordonouri O, Birkebaek NH, Iotova V, Piccini B (2022). Increased prevalence of cardiovascular risk factors in children and adolescents with type 1 diabetes and hypertension: the SWEET international database. Diabetes Obes Metab.

[25] Fang M, Wang D, Coresh J, Selvin E (2021). Trends in diabetes treatment and control in U.S. adults, 1999–2018. N Engl J Med.

[26] Wang L, Li X, Wang Z, Bancks MP, Carnethon MR, Greenland P (2021). Trends in prevalence of diabetes and control of risk factors in diabetes among US adults, 1999–2018. JAMA.

[27] National Center for Health Statistics, Centers for Disease Control and Prevention. National health and nutrition examination survey. https://www.cdc.gov/nchs/nhanes/index.htm. Accessed 10 Jul 2023.

[28] Zipf G, Chiappa M, Porter KS, Ostchega Y, Lewis BG, Dostal J. National health and nutrition examination survey: plan and operations, 1999–2010. Vital Health Stat 1. 2013;(56):1–37. 25078429

[29] National Center for Health Statistics, Centers for Disease Control and Prevention. NHANES survey methods and analytic guidelines. https://wwwn.cdc.gov/nchs/nhanes/analyticguidelines.aspx. Accessed 10 Jul 2023.

[30] National Center for Health Statistics, Centers for Disease Control and Prevention. NCHS research ethics review board (ERB) approval. https://www.cdc.gov/nchs/nhanes/irba98.htm. Accessed 10 Jul 2023.

[31] von Elm E, Altman DG, Egger M, Pocock SJ, Gøtzsche PC, Vandenbroucke JP (2007). The Strengthening the Reporting of Observational Studies in Epidemiology (STROBE) statement: guidelines for reporting observational studies. Lancet.

[32] Krebs-Smith SM, Pannucci TE, Subar AF, Kirkpatrick SI, Lerman JL, Tooze JA (2018). Update of the Healthy Eating Index: HEI-2015. J Acad Nutr Diet.

[33] Flynn JT, Kaelber DC, Baker-Smith CM, Blowey D, Carroll AE, Daniels SR (2017). Clinical practice guideline for screening and management of high blood pressure in children and adolescents. Pediatrics.

[34] Whelton PK, Carey RM, Aronow WS, Casey DE, Collins KJ, Dennison Himmelfarb C (2018). 2017 ACC/AHA/AAPA/ABC/ACPM/AGS/APhA/ASH/ASPC/NMA/PCNA guideline for the prevention, detection, evaluation, and management of high blood pressure in adults: a report of the American College of Cardiology/American Heart Association Task Force on Clinical Practice Guidelines. J Am Coll Cardiol.

[35] ElSayed NA, Aleppo G, Aroda VR, Bannuru RR, Brown FM, Bruemmer D (2023). 2. Classification and diagnosis of diabetes: standards of care in diabetes-2023. Diabetes Care.

[36] National Heart, Lung and Blood Institute (2011). Expert panel on integrated guidelines for cardiovascular health and risk reduction in children and adolescents: summary report. Pediatrics.

[37] Grundy SM, Stone NJ, Bailey AL, Beam C, Birtcher KK, Blumenthal RS (2019). 2018 AHA/ACC/AACVPR/AAPA/ABC/ACPM/ADA/AGS/APhA/ASPC/NLA/PCNA guideline on the management of blood cholesterol: a report of the American College of Cardiology/American Heart Association Task Force on Clinical Practice Guidelines. Circulation.

[38] Cole TJ, Bellizzi MC, Flegal KM, Dietz WH (2000). Establishing a standard definition for child overweight and obesity worldwide: international survey. BMJ.

[39] Gentzke AS, Creamer M, Cullen KA, Ambrose BK, Willis G, Jamal A, King BA (2019). Vital signs: tobacco product use among middle and high school students - United States, 2011–2018. MMWR Morb Mortal Wkly Rep.

[40] Creamer MR, Everett Jones S, Gentzke AS, Jamal A, King BA (2020). Tobacco product use among high school students - youth risk behavior survey, United States, 2019. MMWR Suppl.

[41] Piercy KL, Troiano RP, Ballard RM, Carlson SA, Fulton JE, Galuska DA (2018). The physical activity guidelines for Americans. JAMA.

[42] Willey J, Wakefield M, Silver HJ (2020). Exploring the diets of adults with obesity and type II diabetes from nine diverse countries: dietary intakes, patterns, and quality. Nutrients.

[43] DiMeglio LA, Acerini CL, Codner E, Craig ME, Hofer SE, Pillay K, Maahs DM (2018). ISPAD clinical practice consensus guidelines 2018: glycemic control targets and glucose monitoring for children, adolescents, and young adults with diabetes. Pediatr Diabetes.

[44] ElSayed NA, Aleppo G, Aroda VR, Bannuru RR, Brown FM, Bruemmer D (2023). 14. Children and adolescents: standards of care in diabetes-2023. Diabetes Care.

[45] Xu B, Radojčić MR, Anderson DB, Shi B, Yao L, Chen Y (2024). Trends in prevalence of fractures among adults in the United States, 1999–2020: a population-based study. Int J Surg.

[46] Chobanian AV, Bakris GL, Black HR, Cushman WC, Green LA, Izzo JL (2003). Seventh report of the joint national committee on prevention, detection, evaluation, and treatment of high blood pressure. Hypertension.

[47] National High Blood Pressure Education Program Working Group on High Blood Pressure in Children and Adolescents (2004). The fourth report on the diagnosis, evaluation, and treatment of high blood pressure in children and adolescents. Pediatrics.

[48] Stierman B, Ogden CL, Yanovski JA, Martin CB, Sarafrazi N, Hales CM (2021). Changes in adiposity among children and adolescents in the United States, 1999–2006 to 2011–2018. Am J Clin Nutr.

[49] Ogden CL, Fryar CD, Martin CB, Freedman DS, Carroll MD, Gu Q, Hales CM (2020). Trends in ibesity prevalence by race and Hispanic origin-1999-2000 to 2017–2018. JAMA.

[50] Hales CM, Fryar CD, Carroll MD, Freedman DS, Ogden CL (2018). Trends in obesity and severe obesity prevalence in US youth and adults by sex and age, 2007–2008 to 2015–2016. JAMA.

[51] Ogden CL, Carroll MD, Curtin LR, McDowell MA, Tabak CJ, Flegal KM (2006). Prevalence of overweight and obesity in the United States, 1999–2004. JAMA.

[52] Skinner AC, Perrin EM, Moss LA, Skelton JA (2015). Cardiometabolic risks and severity of obesity in children and young adults. N Engl J Med.

[53] Pastore I, Bolla AM, Montefusco L, Lunati ME, Rossi A, Assi E (2020). The impact of diabetes mellitus on cardiovascular risk onset in children and adolescents. Int J Mol Sci.

[54] Agbaje AO, Lloyd-Jones DM, Magnussen CG, Tuomainen TP (2023). Cumulative dyslipidemia with arterial stiffness and carotid IMT progression in asymptomatic adolescents: a simulated intervention longitudinal study using temporal inverse allocation model. Atherosclerosis.

[55] Nicklas TA, Baranowski T, Cullen KW, Berenson G (2001). Eating patterns, dietary quality and obesity. J Am Coll Nutr.

[56] James J, Kerr D (2005). Prevention of childhood obesity by reducing soft drinks. Int J Obes (Lond).

[57] Mendoza JA, Watson K, Nguyen N, Cerin E, Baranowski T, Nicklas TA (2011). Active commuting to school and association with physical activity and adiposity among US youth. J Phys Act Health.

[58] Yang L, Cao C, Kantor ED, Nguyen LH, Zheng X, Park Y (2019). Trends in sedentary behavior among the US population, 2001–2016. JAMA.

[59] Agbaje AO, Perng W, Tuomainen TP (2023). Effects of accelerometer-based sedentary time and physical activity on DEXA-measured fat mass in 6059 children. Nat Commun.

[60] Brown V, Sheppard L, Salmon J, Arundell L, Cerin E, Ridgers ND (2024). Cost-effectiveness of reducing children's sedentary time and increasing physical activity at school: the Transform-Us! intervention. Int J Behav Nutr Phys Act.

[61] Miguel-Berges ML, Mouratidou T, Santaliestra-Pasias A, Androutsos O, Iotova V, Galcheva S (2023). Longitudinal associations between diet quality, sedentary behaviours and physical activity and risk of overweight and obesity in preschool children: the ToyBox-study. Pediatr Obes.

[62] Sørensen LMN, Aamodt G, Brantsæter AL, Meltzer HM, Papadopoulou E (2022). Diet quality of Norwegian children at 3 and 7 years: changes, predictors and longitudinal association with weight. Int J Obes (Lond).

[63] Mead E, Brown T, Rees K, Azevedo LB, Whittaker V, Jones D (2017). Diet, physical activity and behavioural interventions for the treatment of overweight or obese children from the age of 6 to 11 years. Cochrane Database Syst Rev.

[64] Pyper E, Harrington D, Manson H (2016). The impact of different types of parental support behaviours on child physical activity, healthy eating, and screen time: a cross-sectional study. BMC Public Health.

[65] Jackson SL, Zhang Z, Wiltz JL, Loustalot F, Ritchey MD, Goodman AB, Yang Q (2018). Hypertension among youths - United States, 2001–2016. MMWR Morb Mortal Wkly Rep.

[66] Hardy ST, Sakhuja S, Jaeger BC, Urbina EM, Suglia SF, Feig DI, Muntner P (2021). Trends in blood pressure and hypertension among US children and adolescents, 1999–2018. JAMA Netw Open.

[67] Zachariah JP, Graham DA, de Ferranti SD, Vasan RS, Newburger JW, Mitchell GF (2014). Temporal trends in pulse pressure and mean arterial pressure during the rise of pediatric obesity in US children. J Am Heart Assoc.

[68] Zachariah JP, Wang Y, Penny DJ, Baranowski T (2018). Relation between lead exposure and trends in blood pressure in children. Am J Cardiol.

[69] George MG, Tong X, Wigington C, Gillespie C, Hong Y (2014). Hypertension screening in children and adolescents–national ambulatory medical care survey, national hospital ambulatory medical care survey, and medical expenditure panel survey, United States, 2007–2010. MMWR Suppl.

[70] Hassan MA, Zhou W, Ye M, He H, Gao Z (2024). The effectiveness of physical activity interventions on blood pressure in children and adolescents: a systematic review and network meta-analysis. J Sport Health Sci.

[71] Simonetti GD, Schwertz R, Klett M, Hoffmann GF, Schaefer F, Wühl E (2011). Determinants of blood pressure in preschool children: the role of parental smoking. Circulation.

[72] Overwyk KJ, Zhao L, Zhang Z, Wiltz JL, Dunford EK, Cogswell ME (2019). Trends in blood pressure and usual dietary sodium intake among children and adolescents, National Health and Nutrition Examination Survey 2003 to 2016. Hypertension.

[73] US Food and Drug Administration (2018). Final determination regarding partially hydrogenated oils. Fed Regist.

[74] Brownell KD, Pomeranz JL (2014). The trans-fat ban–food regulation and long-term health. N Engl J Med.

[75] Aggarwal R, Yeh RW, Joynt Maddox KE, Wadhera RK (2023). Cardiovascular risk factor prevalence, treatment, and control in US adults aged 20 to 44 years, 2009 to March 2020. JAMA.

[76] Dai J, Dai W, Li WQ (2023). Trends in physical activity and sedentary time among U.S. adults with diabetes: 2007–2020. Diabetes Metab Syndr..

[77] Walli-Attaei M, Rosengren A, Rangarajan S, Breet Y, Abdul-Razak S, Sharief WA (2022). Metabolic, behavioural, and psychosocial risk factors and cardiovascular disease in women compared with men in 21 high-income, middle-income, and low-income countries: an analysis of the PURE study. Lancet.

[78] Peters SAE, Muntner P, Woodward M (2019). Sex differences in the prevalence of, and trends in, cardiovascular risk factors, treatment, and control in the United States, 2001 to 2016. Circulation.

[79] Gillis EE, Sullivan JC (2016). Sex differences in hypertension: recent advances. Hypertension.

[80] Hermes Z, Joynt Maddox KE, Yeh RW, Zhao Y, Shen C, Wadhera RK (2022). Neighborhood socioeconomic disadvantage and mortality among Medicare beneficiaries hospitalized for acute myocardial infarction, heart failure, and pneumonia. J Gen Intern Med.

[81] Maldonado LE, Albrecht SS (2018). Does the immigrant advantage in overweight/obesity persist over time in Mexican American youth? NHANES 1988–1994 to 2005–2014. Obesity (Silver Spring).

[82] Duffey KJ, Popkin BM (2007). Shifts in patterns and consumption of beverages between 1965 and 2002. Obesity (Silver Spring).

[83] Van Hook J, Baker E, Altman CE, Frisco ML (2012). Canaries in a coalmine: immigration and overweight among Mexican-origin children in the US and Mexico. Soc Sci Med.

[84] Oza-Frank R, Cunningham SA (2010). The weight of US residence among immigrants: a systematic review. Obes Rev.

[85] Carroll AJ, Tedla YG, Padilla R, Jain A, Segovia E, Moin A (2023). Adherence to the 2017 clinical practice guidelines for pediatric hypertension in safety-net clinics. JAMA Netw Open.

