# Correction: Trends in cardiovascular risk factor prevalence, treatment, and control among US adolescents aged 12 to 19 years, 2001 to March 2020

**DOI:** 10.1186/s12916-024-03521-w

**Published:** 2024-07-11

**Authors:** Qiang Qu, Qixin Guo, Jinjing Shi, Ziqi Chen, Jinyu Sun, Iokfai Cheang, Rongrong Gao, Yanli Zhou, Haifeng Zhang, Shengen Liao, Wenming Yao, Xinli Li

**Affiliations:** 1 State Key Laboratory for Innovation and Transformation of Luobing Theory, Department of Cardiology, The First Affiliated Hospital of Nanjing Medical University, 300 Guangzhou Road, Nanjing, 210029 China; 2 Department of Cardiology, Gusu School, The Affiliated Suzhou Hospital of Nanjing Medical University, Suzhou Municipal Hospital, Nanjing Medical University, 26 Daoqian Street, Suzhou, 215002 China; 3 Department of Cardiology, Jiangsu Province Hospital, 300 Guangzhou Road, Nanjing, 210029 China

**Correction** **:** **BMC Med 22, 245 (2024)**

**https://doi.org/10.1186/s12916-024-03453-5**

The original article [1] contains the following errors which the authors would like to clarify:

1) In Additional file 1: eFigure 1, the number ‘9306' was inadvertently inverted and should be removed.

2) The original article contained a typo in the ‘Abstract' section. In the sentence, ‘The variation by sociodemographic characteristics were also described.'; the word ‘variation’ should instead state ‘variations’.

3) The original article also contained an error in the following sentence in the ‘CVRF treatment and control rates’ sub-section of the Results section:

‘Among adolescents with diabetes, the use of any antidiabetic medication did not change significantly, from 51.0% (95% CI, 23.3%-78.7%) in 2001–2004 to 26.5% (95% CI, 0.0%-54.7%) in 2017-March 2020 (P for trend = 0.60) (Fig. 2B and Additional File 1: eTable 4).'

The word ‘File' should instead state ‘file'.

4) In the third footnote to Additional file 1: eTable 4, ‘The definition of hypertension was identical to that used in the main analysis.’ should be replaced as ‘The definition of hypertension treatment was identical to that used in the main analysis.’
