# Supplementary material for: Trends in cardiovascular risk factor prevalence, treatment, and control among US adolescents aged 12 to 19 years, 2001 to March 2020
Source: BMC Med. 2024 Jun 13;22:245. doi: 10.1186/s12916-024-03453-5 (PMC11170826; doi:10.1186/s12916-024-03453-5)
Supplement: Supplementary file 1 — Additional file 1: eMethod 1. Therapeutic Drug Classes Used to Define Any Use of Antihypertensive and Antidiabetic Medications. eFigure 1. Inclusion Diagram for US Adolescents Aged 12 to 19 Years, 2001 to March 2020. eFigure 2. Age-Adjusted Trends in Mean BP, Hemoglobin A1c, FPG, TC, HDL-C, Non-HDL-C, LDL-C, Triglycerides Levels, Body Mass Index, Weekly Exercise Time, and HEI-2015 for US Adolescents Aged 12 to 19 Years, 2001 to March 2020. eFigure 3. Age-Adjusted Trends in Mean BP, Hemoglobin A1c, FPG, TC, HDL-C, Non-HDL-C, LDL-C, Triglycerides Levels, Body Mass Index, Weekly Exercise Time, and HEI-2015 for US Adolescents Aged 12 to 19 Years by Sex, 2001 to March 2020. eFigure 4. Age-Adjusted Trends in Mean BP, Hemoglobin A1c, FPG, TC, HDL-C, Non-HDL-C, LDL-C, Triglycerides Levels, Body Mass Index, Weekly Exercise Time, and HEI-2015 for US Adolescents Aged 12 to 19 Years by Race/Ethnicity, 2001 to March 2020. eFigure 5. Age-Adjusted Trends in the Prevalence of Hypertension, Elevated BP, Diabetes, Prediabetes, Hyperlipidemia, Obesity, Overweight, Cigarette Use, Inactive Physical Activity, and Poor Diet Quality Among US Adolescents Aged 12 to 19 Years by Sex, 2001 to March 2020. eFigure 6. Age-Adjusted Trends in the Prevalence of Hypertension, Elevated BP, Diabetes, Prediabetes, Hyperlipidemia, Obesity, Overweight, Cigarette Use, Inactive Physical Activity, and Poor Diet Quality Among US Adolescents Aged 12 to 19 Years by Race/Ethnicity, 2001 to March 2020. Years by Race/Ethnicity, 2001 to March 2020. eFigure 7. Age-Adjusted Trends in the Prevalence of High BP and Hypertension Treatment and Control Rates Among US Adolescents Aged 12 to 19 Years According to the 2003 NIH/NHLBI and 2004 NIH/NHLBI Guidelines, 2001 to March 2020. eTable 1. Unweighted Response Rates for the NHANES In-Home Interviews and Mobile Examinations Among US Adolescents Aged 12 to 19 Years by Age and Sex Groups, 2001 to March 2020. eTable 2. Strengthening the Reporting of Observational Studies in Ep [file 12916_2024_3453_MOESM1_ESM.pdf]

Additional file 1

Trends in Cardiovascular Risk Factor Prevalence, Treatment, and Control among US Youths Aged 12 to 19 Years, 2001 to March 2020

Qiang Qu, MD, Qixin Guo, MD, Jinjing Shi, MD, Ziqi Chen, MD, Jinyu Sun, MD, Iokfai Cheang, MD, Rongrong Gao, MD, PhD, Yanli Zhou, MD, PhD, Haifeng Zhang, MD, PhD, Shengen Liao, MD, PhD, Wenming Yao, MD, PhD, Xinli Li, MD, PhD

eMethod 1. Therapeutic Drug Classes Used to Define Any Use of Antihypertensive and Antidiabetic Medications 1

eFigure 1. Inclusion Diagram for US Adolescents Aged 12 to 19 Years, 2001 to March 2020 2

eFigure 2. Age-Adjusted Trends in Mean BP, Hemoglobin A_{1c}, FPG, TC, HDL-C, Non-HDL-C, LDL-C, Triglycerides Levels, Body Mass Index, Weekly Exercise Time, and HEI-2015 for US Adolescents Aged 12 to 19 Years, 2001 to March 2020 3

eFigure 3. Age-Adjusted Trends in Mean BP, Hemoglobin A_{1c}, FPG, TC, HDL-C, Non-HDL-C, LDL-C, Triglycerides Levels, Body Mass Index, Weekly Exercise Time, and HEI-2015 for US Adolescents Aged 12 to 19 Years by Sex, 2001 to March 2020 5

eFigure 4. Age-Adjusted Trends in Mean BP, Hemoglobin A_{1c}, FPG, TC, HDL-C, Non-HDL-C, LDL-C, Triglycerides Levels, Body Mass Index, Weekly Exercise Time, and HEI-2015 for US Adolescents Aged 12 to 19 Years by Race/Ethnicity, 2001 to March 2020 7

eFigure 5. Age-Adjusted Trends in the Prevalence of Hypertension, Elevated BP, Diabetes, Prediabetes, Hyperlipidemia, Obesity, Overweight, Cigarette Use, Inactive Physical Activity, and Poor Diet Quality Among US Adolescents Aged 12 to 19 Years by Sex, 2001 to March 2020 9

eFigure 6. Age-Adjusted Trends in the Prevalence of Hypertension, Elevated BP, Diabetes, Prediabetes, Hyperlipidemia, Obesity, Overweight, Cigarette Use, Inactive Physical Activity, and Poor Diet Quality Among US Adolescents Aged 12 to 19 Years by Race/Ethnicity, 2001 to March 2020 11

eFigure 7. Age-Adjusted Trends in the Prevalence of High BP and Hypertension Treatment and Control Rates Among US Adolescents Aged 12 to 19 Years According to the 2003 NIH/NHLBI and 2004 NIH/NHLBI Guidelines, 2001 to March 2020 13

eTable 1. Unweighted Response Rates for the NHANES In-Home Interviews and Mobile Examinations Among US Adolescents Aged 12 to 19 Years by Age and Sex Groups, 2001 to March 2020 14

eTable 2. Strengthening the Reporting of Observational Studies in Epidemiology (STROBE) Reporting Guideline for Reporting Cross-sectional Studies Checklist 16

eTable 3. Classification of BP by the 2003 NIH/NHLBI, 2004 NIH/NHLBI, 2017 AAP, and 2017 ACC/AHA Guidelines 18

eTable 4. Trends in Age-Adjusted Means or % (95% CIs) of Cardiovascular Parameters, Cardiovascular Risk Factors, and Hypertension and Diabetes Treatment and Control Among US Adolescents Aged 12 to 19 Years by Age and Racial/Ethnic Groups, 2001 to March 2020 19

eTable 5. Age-Adjusted Rates of Hypertension and Diabetes Treatment and Control by Subgroups Among US Adolescents Aged 12 to 19 Years, 2001 to March 2020 27

eTable 6. Adjusted ORs for Hypertension and Diabetes Treatment and Control by Subgroups Among US Adolescents Aged 12 to 19 Years, 2001 to March 2020 29

eTable 7. Comparison of Baseline Characteristics Between the Included and Excluded Study Population 31

eTable 8. Baseline Characteristics of the Excluded Study Population, 2001 to March 2020 32

eMethod 1. Therapeutic Drug Classes Used to Define Any Use of Antihypertensive and Antidiabetic Medications

Information on prescription medications taken by participants were obtained in the National Health and Nutrition Examination Survey using questionnaire interviews. Participants were asked whether they had taken any prescription medication within the previous 30 days. Those who answered ‘yes’ were requested to present the containers of all the medications used. When no container was available, participants were asked to provide the names of medications verbally. These medications were divided into therapeutic drug classes via Multum’s Lexicon classification system.

We defined the use of antihypertensive medications as the use of any of the following therapeutic drug classes:

- angiotensin converting enzyme inhibitors
- angiotensin receptor blockers
- adrenergic blocking agents
- alpha-blockers
- beta-blockers
- calcium channel blocking agents
- vasodilators
- diuretics
- renin inhibitors
- aldosterone receptor antagonists
- other antihypertensive agents

We defined the use of antidiabetic medications as the use of any of the following therapeutic drug classes:

- biguanides
- sulfonylureas
- alpha-glucosidase inhibitors
- meglitinides
- thiazolidinediones
- dipeptidyl peptidase-4 inhibitors
- glucose like peptide-1 receptor agonists
- sodium-glucose co-transporter-2 inhibitors
- insulin
- amylin analogs
- other antidiabetic agents

eFigure 1. Inclusion Diagram for US Adolescents Aged 12 to 19 Years, 2001 to March 2020[a-b]

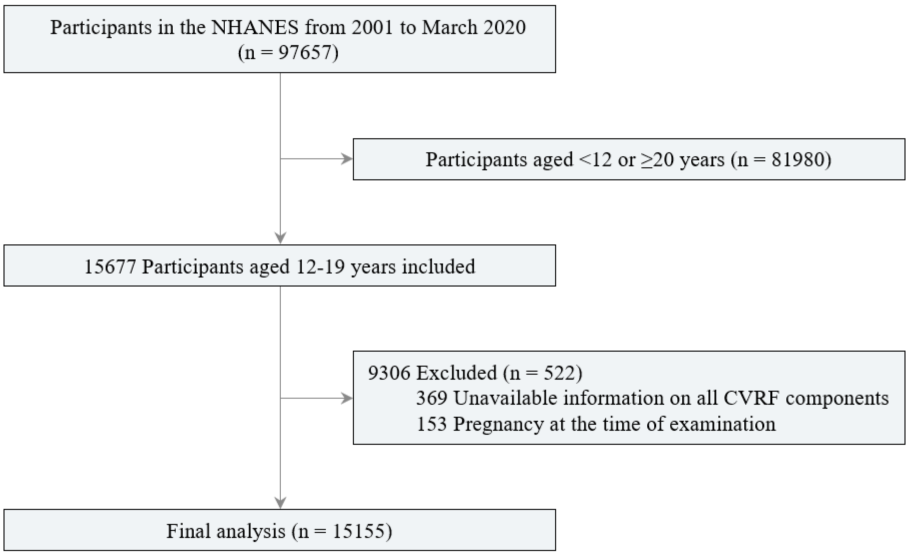

Abbreviations: CVRF, cardiovascular risk factor; NHANES, National Health and Nutrition Examination Survey.

[a] Nationally representative estimates of US adolescents aged 12-19 years from the 2001-March 2020 NHANES.

[b] CVRF components included hypertension, elevated blood pressure, diabetes, prediabetes, hyperlipidemia, obesity, overweight, cigarette use, inactive physical activity, and poor diet quality.

eFigure 2. Age-Adjusted Trends in Mean BP, Hemoglobin A_{1c}, FPG, TC, HDL-C, Non-HDL-C, LDL-C, Triglycerides Levels, Body Mass Index, Weekly Exercise Time, and HEI-2015 for US Adolescents Aged 12 to 19 Years, 2001 to March 2020[a-e]

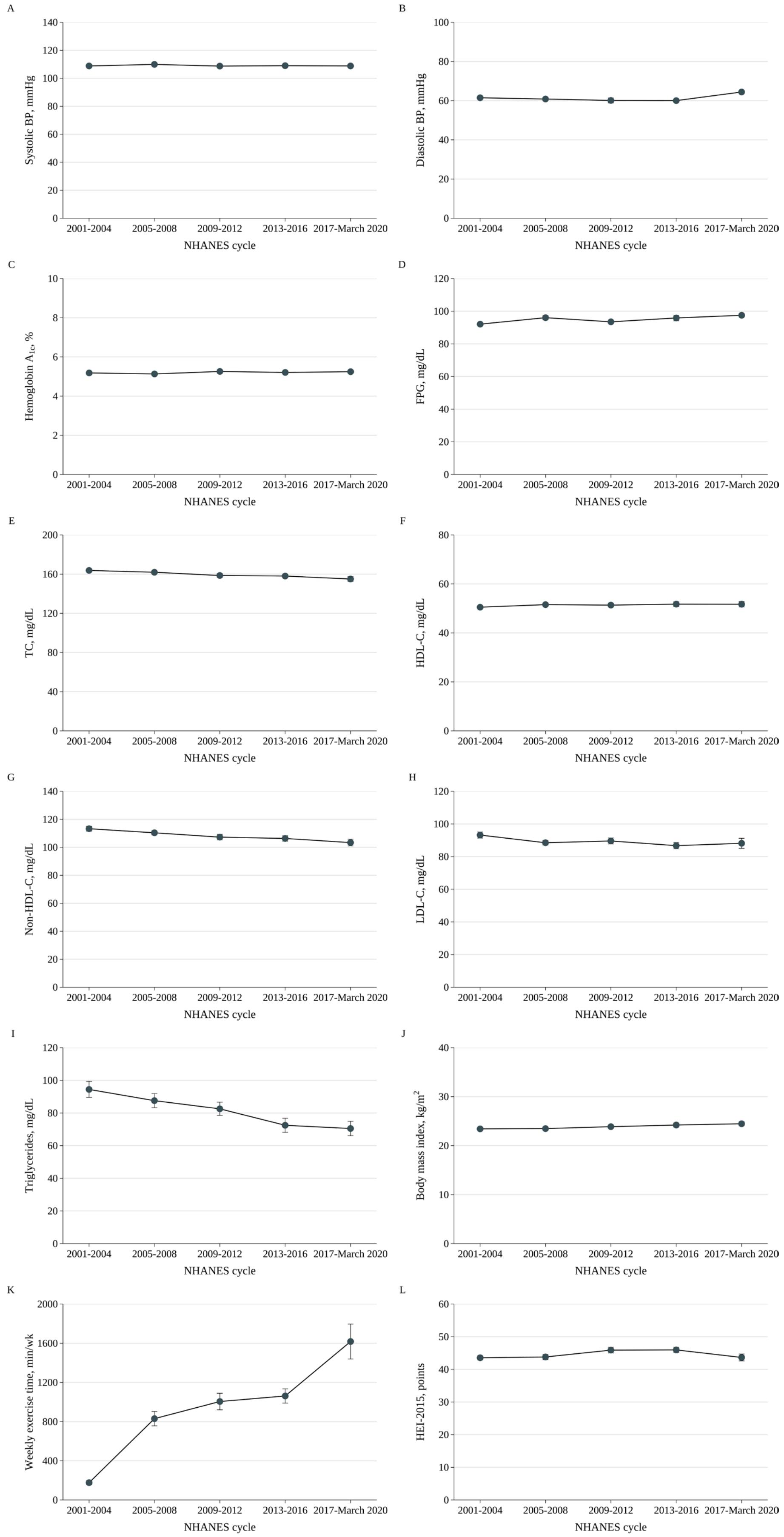

Abbreviations: BP, blood pressure; CI, confidence interval; FPG, fasting plasma glucose; HDL-C, high-density lipoprotein cholesterol; HEI-2015, Healthy Eating Index-2015; LDL-C, low-density lipoprotein cholesterol; non-HDL-C, non-high-density lipoprotein cholesterol; TC, total cholesterol.

SI conversions: to convert glucose to mmol/L, multiply by 0.0555; TC, HDL-C, non-HDL-C, and LDL-C to mmol/L, multiply by 0.0259; triglycerides to mmol/L, multiply by 0.0113.

[a] Nationally representative estimates of US adolescents aged 12-19 years from the 2001-March 2020 National Health and Nutrition Examination Survey. Whiskers indicate 95% CIs. *P* for trend was calculated by the Joinpoint Regression Program: *P* = .81 for systolic BP in panel A; *P* = .28 for diastolic BP in panel B; *P* = .27 for hemoglobin A_{1c} in panel C; *P* = .07 for FPG in panel D; *P* = .003 for TC in panel E; *P* = .08 for HDL-C in panel F; *P* = .001 for non-HDL-C in panel G; *P* = .15 for LDL-C in panel H; *P* = .002 for triglycerides in panel I; *P* = .003 for body mass index in panel J; *P* = .08 for weekly exercise time in panel K; and *P* = .34 for HEI-2015 in panel L. Specific estimates are presented in eTable 4.

[b] All estimates were age-standardized to the 2000 Census population using the age groups of 12 to 14, 15 to 17, and 18 to 19 years.

[c] Non-HDL-C was calculated as the difference between serum TC and HDL-C.

[d] FPG, LDL-C, and triglycerides were based on fasting laboratory testing.

[e] Weekly exercise time was calculated as the minutes of moderate-intensity physical activity plus twice the minutes of vigorous-intensity physical activity per week. During 2001-2006, physical activity levels were underestimated due to a lack of data on home/yard tasks for adolescents aged 12-15 years and a lack of time data on muscle-strengthening activities across the age spectrum; data on weekly exercise time for participants aged 12-17 years were not available during 2017-March 2020, and thus estimates only represented those aged 18-19 years throughout this period.

eFigure 3. Age-Adjusted Trends in Mean BP, Hemoglobin A_{1c}, FPG, TC, HDL-C, Non-HDL-C, LDL-C, Triglycerides Levels, Body Mass Index, Weekly Exercise Time, and HEI-2015 for US Adolescents Aged 12 to 19 Years by Sex, 2001 to March 2020[a-e]

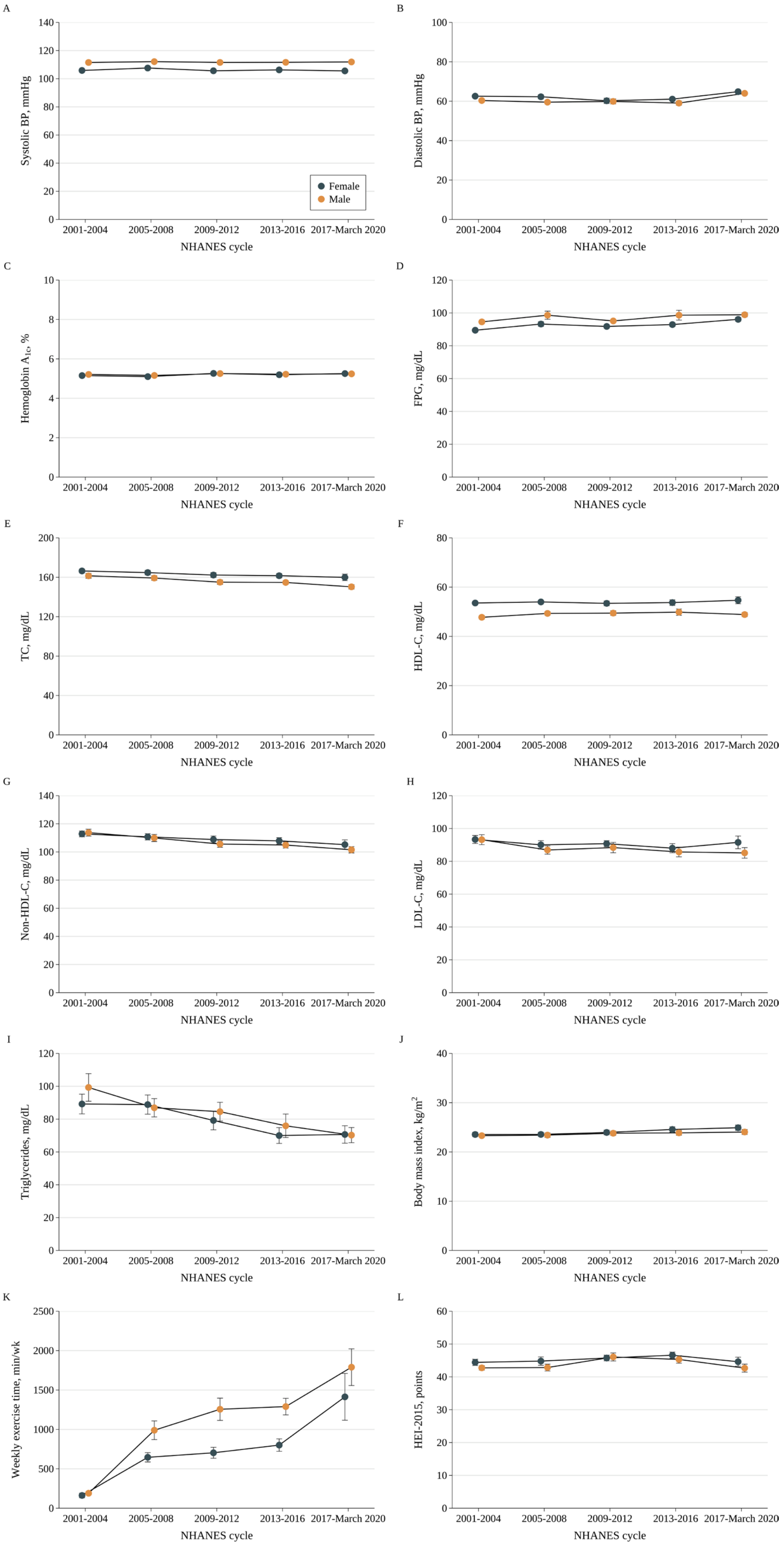

Abbreviations: BP, blood pressure; CI, confidence interval; FPG, fasting plasma glucose; HDL-C, high-density lipoprotein cholesterol; HEI-2015, Healthy Eating Index-2015; LDL-C, low-density lipoprotein cholesterol; non-HDL-C, non-high-density lipoprotein cholesterol; TC, total cholesterol.

SI conversions: to convert glucose to mmol/L, multiply by 0.0555; TC, HDL-C, non-HDL-C, and LDL-C to mmol/L, multiply by 0.0259; triglycerides to mmol/L, multiply by 0.0113.

[a] Nationally representative estimates of US adolescents aged 12-19 years from the 2001-March 2020 National Health and Nutrition Examination Survey. Estimates are shown by sex; whiskers indicate 95% CIs. Sex was based on self-report. Specific estimates of *P* for trend calculated by the Joinpoint Regression Program are presented in eTable 4.

[b] All estimates were age-standardized to the 2000 Census population using the age groups of 12 to 14, 15 to 17, and 18 to 19 years.

[c] Non-HDL-C was calculated as the difference between serum TC and HDL-C.

[d] FPG, LDL-C, and triglycerides were based on fasting laboratory testing.

[e] Weekly exercise time was calculated as the minutes of moderate-intensity physical activity plus twice the minutes of vigorous-intensity physical activity per week. During 2001-2006, physical activity levels were underestimated due to a lack of data on home/yard tasks for adolescents aged 12-15 years and a lack of time data on muscle-strengthening activities across the age spectrum; data on weekly exercise time for participants aged 12-17 years were not available during 2017-March 2020, and thus estimates only represented those aged 18-19 years throughout this period.

eFigure 4. Age-Adjusted Trends in Mean BP, Hemoglobin A_{1c}, FPG, TC, HDL-C, Non-HDL-C, LDL-C, Triglycerides Levels, Body Mass Index, Weekly Exercise Time, and HEI-2015 for US Adolescents Aged 12 to 19 Years by Race/Ethnicity, 2001 to March 2020[a-e]

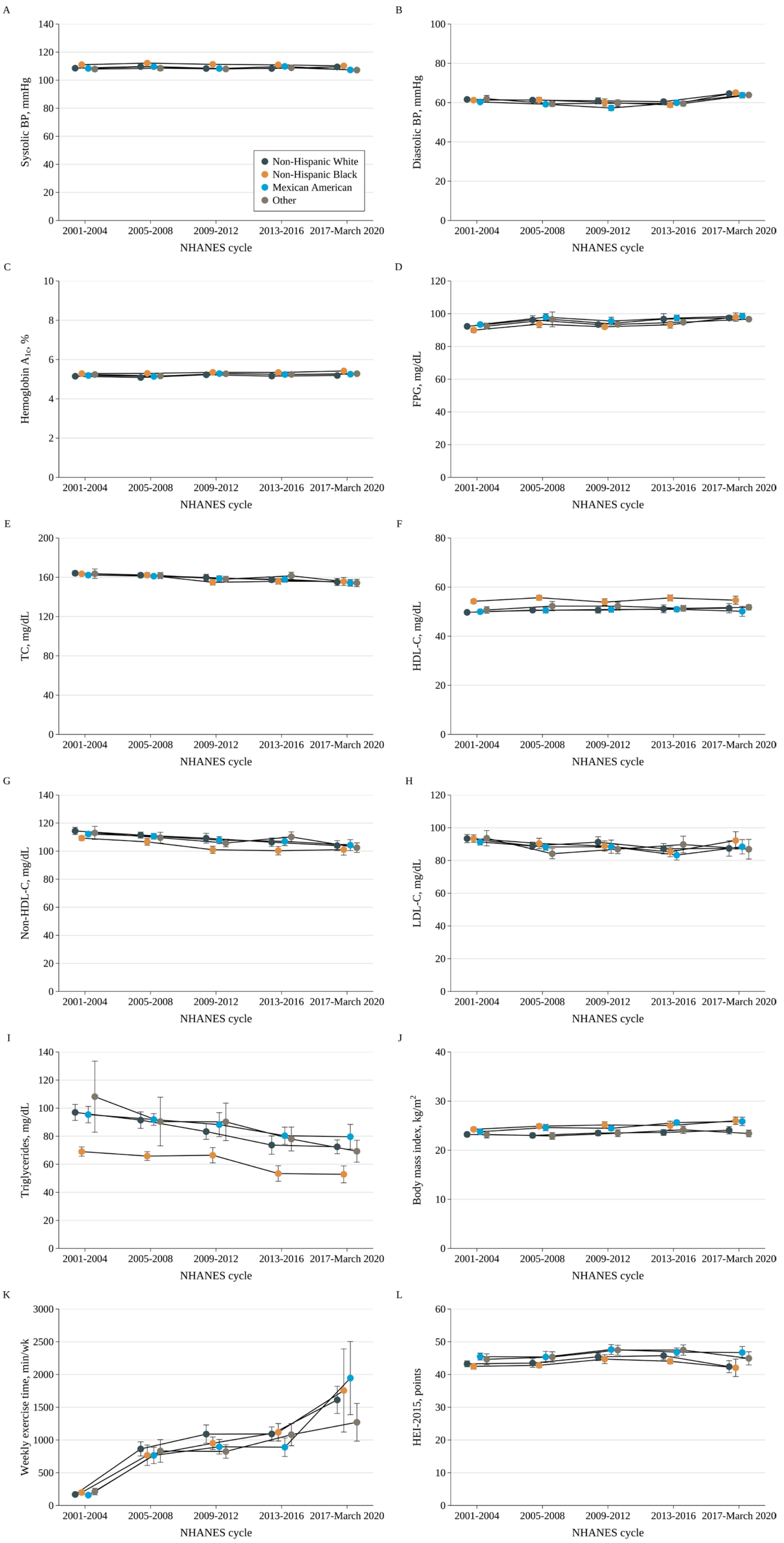

Abbreviations: BP, blood pressure; CI, confidence interval; FPG, fasting plasma glucose; HDL-C, high-density lipoprotein cholesterol; HEI-2015, Healthy Eating Index-2015; LDL-C, low-density lipoprotein cholesterol; non-HDL-C, non-high-density lipoprotein cholesterol; TC, total cholesterol.

SI conversions: to convert glucose to mmol/L, multiply by 0.0555; TC, HDL-C, non-HDL-C, and LDL-C to mmol/L, multiply by 0.0259; triglycerides to mmol/L, multiply by 0.0113.

[a] Nationally representative estimates of US adolescents aged 12-19 years from the 2001-March 2020 National Health and Nutrition Examination Survey. Estimates are shown by race/ethnicity; whiskers indicate 95% CIs. Race/ethnicity was based on self-report. Specific estimates of *P* for trend calculated by the Joinpoint Regression Program are presented in eTable 4.

[b] All estimates were age-standardized to the 2000 Census population using the age groups of 12 to 14, 15 to 17, and 18 to 19 years.

[c] Non-HDL-C was calculated as the difference between serum TC and HDL-C.

[d] FPG, LDL-C, and triglycerides were based on fasting laboratory testing.

[e] Weekly exercise time was calculated as the minutes of moderate-intensity physical activity plus twice the minutes of vigorous-intensity physical activity per week. During 2001-2006, physical activity levels were underestimated due to a lack of data on home/yard tasks for adolescents aged 12-15 years and a lack of time data on muscle-strengthening activities across the age spectrum; data on weekly exercise time for participants aged 12-17 years were not available during 2017-March 2020, and thus estimates only represented those aged 18-19 years throughout this period.

eFigure 5. Age-Adjusted Trends in the Prevalence of Hypertension, Elevated BP, Diabetes, Prediabetes, Hyperlipidemia, Obesity, Overweight, Cigarette Use, Inactive Physical Activity, and Poor Diet Quality Among US Adolescents Aged 12 to 19 Years by Sex, 2001 to March 2020[a-i]

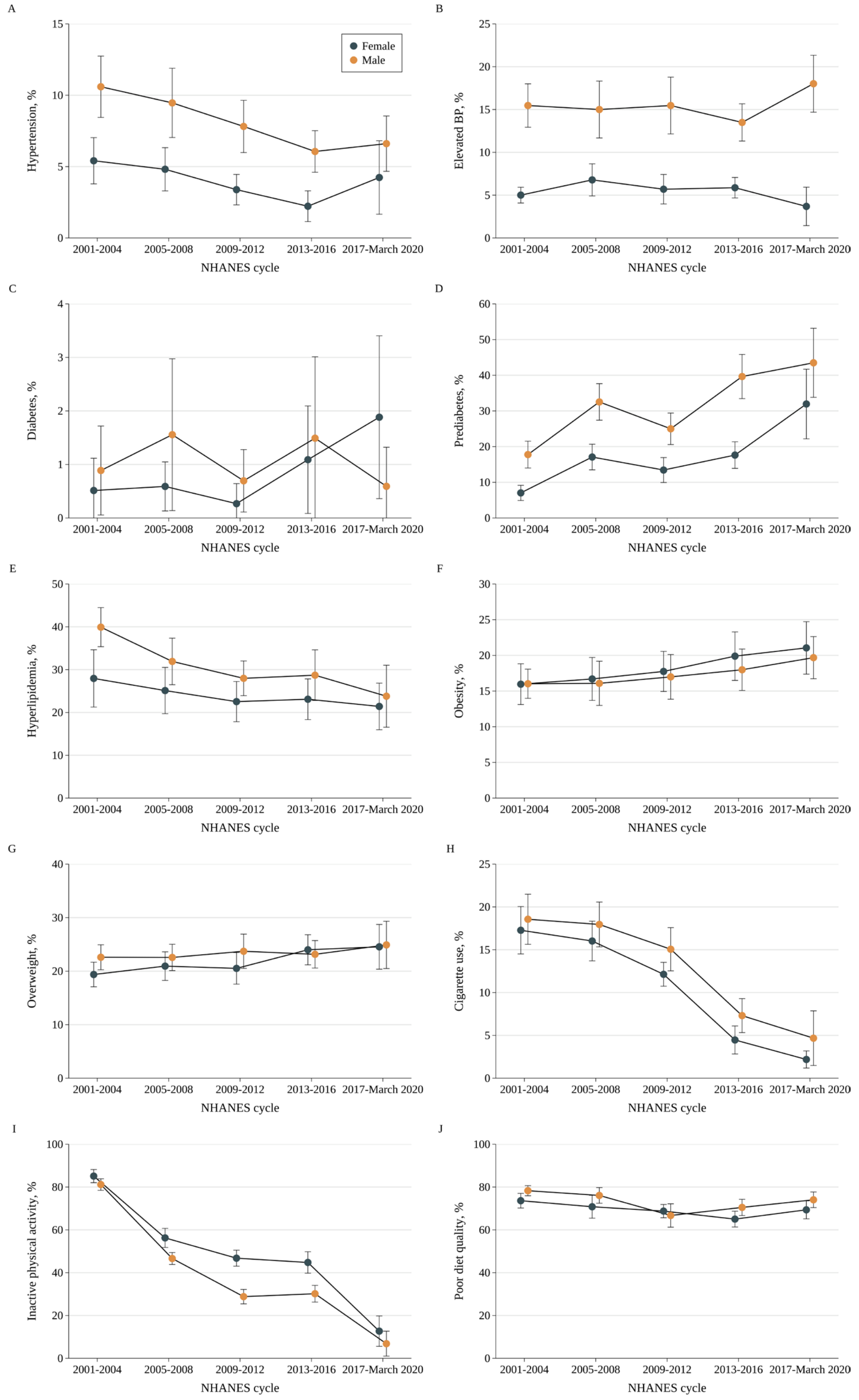

Abbreviations: BP, blood pressure; CI, confidence interval.

[a] Nationally representative estimates of US adolescents aged 12-19 years from the 2001-March 2020 National Health and Nutrition Examination Survey. Estimates are shown by sex; whiskers indicate 95% CIs. Sex was based on self-report. Specific estimates of *P* for trend calculated by the Joinpoint Regression Program are presented in eTable 4.

[b] All estimates were age-standardized to the 2000 Census population using the age groups of 12 to 14, 15 to 17, and 18 to 19 years.

[c] Hypertension was defined as stage 1 or 2 levels and/or current use of antihypertensive medications, whereas elevated BP was defined as an elevated level (see Ref. 33 and 34).

[d] Diabetes was defined as a hemoglobin A_{1c} of ≥6.5%, fasting plasma glucose of ≥126 mg/dL, self-report of previous diagnosis, and/or current use of antidiabetic medications, whereas prediabetes was defined as a hemoglobin A_{1c} of 5.7%-6.4%.

[e] Hyperlipidemia was defined as a total cholesterol of ≥200 mg/dL, high-density lipoprotein cholesterol of <40 mg/dL, non-high-density lipoprotein cholesterol of ≥145 mg/dL, low-density lipoprotein cholesterol of ≥130 mg/dL, triglycerides of ≥130 mg/dL, and/or current use of antihyperlipidemic medications.

[f] Obesity and overweight were defined based on body mass index using the Lambda Mu Sigma method (see Ref. 38).

[g] Cigarette use was defined as smoking cigarettes within the previous 30 days.

[h] Inactive physical activity was defined as a weekly exercise time of <420 and <150 minutes/wk in adolescents aged <18 and 18-19 years, respectively. Weekly exercise time was calculated as the minutes of moderate-intensity physical activity plus twice the minutes of vigorous-intensity physical activity per week. During 2001-2006, physical activity levels were underestimated due to a lack of data on home/yard tasks for adolescents aged 12-15 years and a lack of time data on muscle-strengthening activities across the age spectrum; data on weekly exercise time for participants aged 12-17 years were not available during 2017-March 2020, and thus estimates only represented those aged 18-19 years throughout this period.

[i] Poor diet quality was defined as a Healthy Eating Index-2015 score of <51 points.

eFigure 6. Age-Adjusted Trends in the Prevalence of Hypertension, Elevated BP, Diabetes, Prediabetes, Hyperlipidemia, Obesity, Overweight, Cigarette Use, Inactive Physical Activity, and Poor Diet Quality Among US Adolescents Aged 12 to 19 Years by Race/Ethnicity, 2001 to March 2020[a-i]

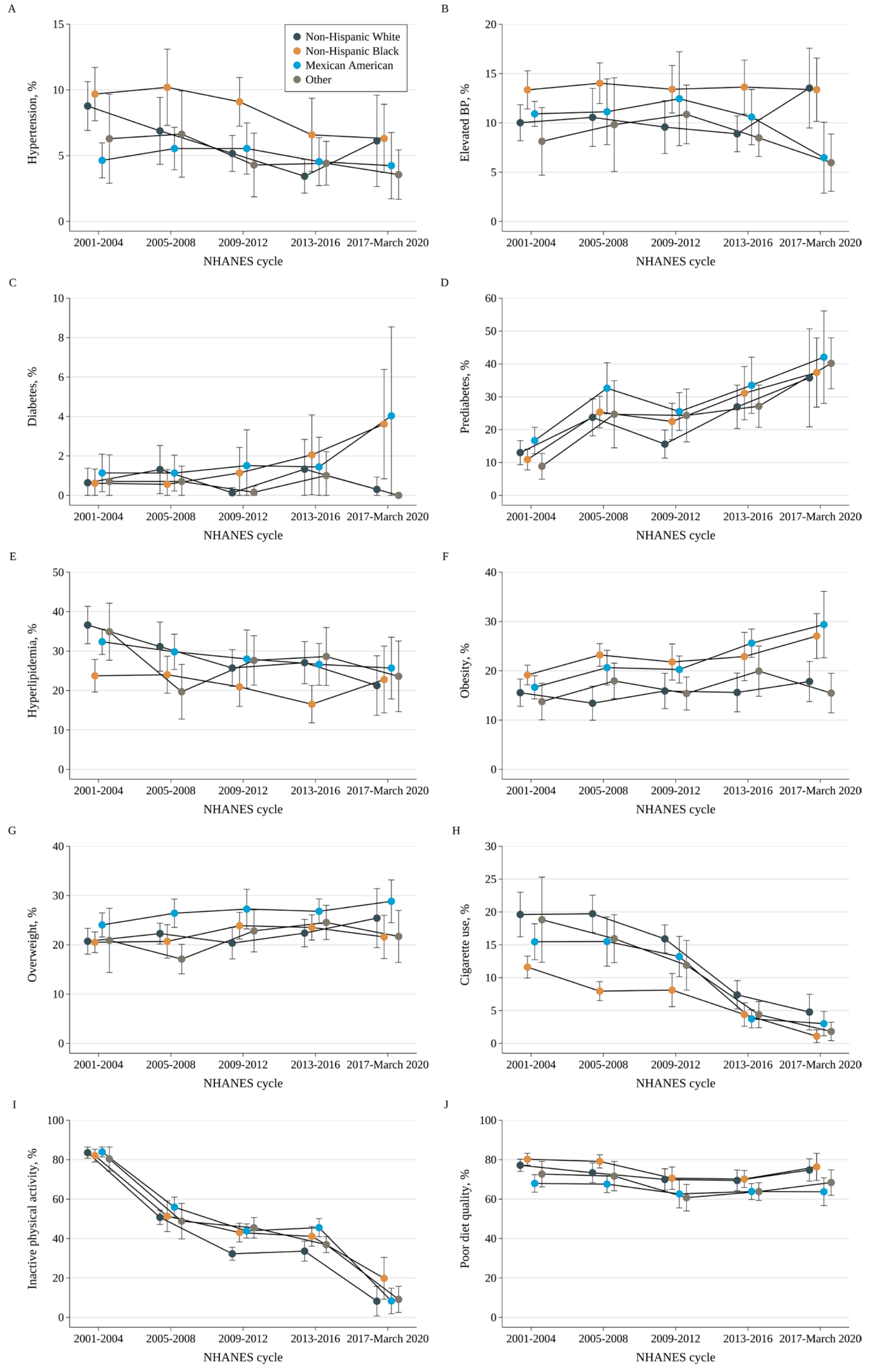

Abbreviations: BP, blood pressure; CI, confidence interval.

[a] Nationally representative estimates of US adolescents aged 12-19 years from the 2001-March 2020 National Health and Nutrition Examination Survey. Estimates are shown by race/ethnicity; whiskers indicate 95% CIs. Race/ethnicity was based on self-report. Specific estimates of *P* for trend calculated by the Joinpoint Regression Program are presented in eTable 4.

[b] All estimates were age-standardized to the 2000 Census population using the age groups of 12 to 14, 15 to 17, and 18 to 19 years.

[c] Hypertension was defined as stage 1 or 2 levels and/or current use of antihypertensive medications, whereas elevated BP was defined as an elevated level (see Ref. 33 and 34).

[d] Diabetes was defined as a hemoglobin A_{1c} of ≥6.5%, fasting plasma glucose of ≥126 mg/dL, self-report of previous diagnosis, and/or current use

of antidiabetic medications, whereas prediabetes was defined as a hemoglobin A_{1c} of 5.7%-6.4%.

[e] Hyperlipidemia was defined as a total cholesterol of ≥200 mg/dL, high-density lipoprotein cholesterol of <40 mg/dL, non-high-density lipoprotein cholesterol of ≥145 mg/dL, low-density lipoprotein cholesterol of ≥130 mg/dL, triglycerides of ≥130 mg/dL, and/or current use of antihyperlipidemic medications.

[f] Obesity and overweight were defined based on body mass index using the Lambda Mu Sigma method (see Ref. 38).

[g] Cigarette use was defined as smoking cigarettes within the previous 30 days.

[h] Inactive physical activity was defined as a weekly exercise time of <420 and <150 minutes/wk in adolescents aged <18 and 18-19 years, respectively. Weekly exercise time was calculated as the minutes of moderate-intensity physical activity plus twice the minutes of vigorous-intensity physical activity per week. During 2001-2006, physical activity levels were underestimated due to a lack of data on home/yard tasks for adolescents aged 12-15 years and a lack of time data on muscle-strengthening activities across the age spectrum; data on weekly exercise time for participants aged 12-17 years were not available during 2017-March 2020, and thus estimates only represented those aged 18-19 years throughout this period.

[i] Poor diet quality was defined as a Healthy Eating Index-2015 score of <51 points.

eFigure 7. Age-Adjusted Trends in the Prevalence of High BP and Hypertension Treatment and Control Rates Among US Adolescents Aged 12 to 19 Years According to the 2003 NIH/NHLBI and 2004 NIH/NHLBI Guidelines, 2001 to March 2020[a-e]

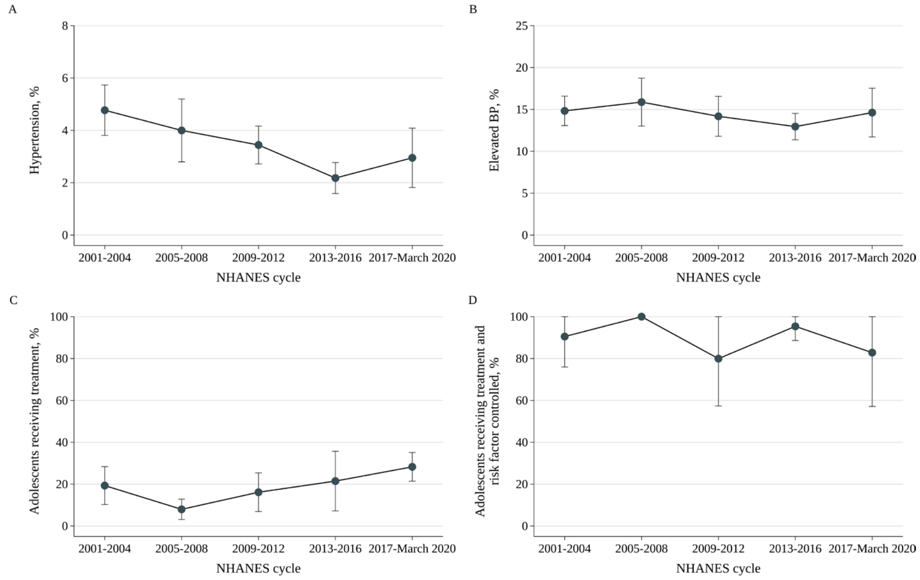

Abbreviations: BP, blood pressure; CI, confidence interval; NIH/NHLBI, National Institutes of Health's National Heart, Lung, and Blood Institute.

[a] Nationally representative estimates of US adolescents aged 12-19 years from the 2001-March 2020 National Health and Nutrition Examination Survey. Whiskers indicate 95% CIs. *P* for trend was calculated by the Joinpoint Regression Program: *P* = .04 for hypertension in panel A; *P* = .27 for elevated BP in panel B; *P* = .17 for hypertension treatment in panel C; and *P* value was not applicable for BP control in panel D. Specific estimates are presented in eTable 4.

[b] All estimates were age-standardized to the 2000 Census population using the age groups of 12 to 14, 15 to 17, and 18 to 19 years.

[c] Hypertension was defined as stage 1 or 2 levels and/or current use of antihypertensive medications, whereas elevated BP was defined as an elevated level (see Ref. 46 and 47).

[d] Hypertension treatment was defined as current use of antihypertensive medications and was evaluated among adolescents with hypertension (n = 528).

[e] Hypertension control was evaluated among adolescents receiving treatment (n = 68). Hypertension was considered controlled if (1) BP was reduced to <95th percentile in adolescents aged <18 years or (2) BP was reduced to <140/90 mmHg in adolescents aged 18-19 years.

eTable 1. Unweighted Response Rates for the NHANES In-Home Interviews and Mobile Examinations Among US Adolescents Aged 12 to 19 Years by Age and Sex Groups, 2001 to March 2020

Year	Unweighted response rates for in-home interviews	Unweighted response rates for mobile examinations
2001-2002		
Overall	88.9%	86.4%
12-15 years	89.3%	87.0%
16-19 years	88.4%	85.8%
Female	89.0%	86.3%
Male	88.8%	86.5%
2003-2004		
Overall	86.9%	84.9%
12-15 years	85.7%	83.8%
16-19 years	88.1%	85.9%
Female	88.2%	86.4%
Male	85.8%	83.5%
2005-2006		
Overall	85.1%	82.1%
12-15 years	85.6%	82.7%
16-19 years	84.5%	81.5%
Female	85.5%	82.0%
Male	84.7%	82.1%
2007-2008		
Overall	85.5%	83.6%
12-15 years	84.8%	82.5%
16-19 years	86.2%	84.6%
Female	85.6%	83.4%
Male	85.4%	83.7%
2009-2010		
Overall	87.8%	85.9%
12-15 years	87.0%	84.9%
16-19 years	88.6%	86.9%
Female	89.0%	87.0%
Male	87.0%	85.0%
2011-2012		
Overall	78.7%	76.1%
12-15 years	81.9%	78.5%
16-19 years	75.6%	73.7%
Female	77.3%	74.3%
Male	80.1%	77.8%
2013-2014		
Overall	78.7%	76.5%
12-15 years	78.3%	75.8%
16-19 years	79.2%	77.2%
Female	79.3%	76.6%
Male	78.2%	76.3%

2015-2016		
Overall	66.5%	64.2%
12-15 years	66.3%	63.8%
16-19 years	66.8%	64.7%
Female	66.9%	64.3%
Male	66.2%	64.2%
2017-March 2020		
Overall	54.4%	50.7%
12-15 years	54.5%	50.6%
16-19 years	54.3%	50.8%
Female	53.9%	50.1%
Male	55.0%	51.3%

Abbreviations: NHANES, National Health and Nutrition Examination Survey.

eTable 2. Strengthening the Reporting of Observational Studies in Epidemiology (STROBE) Reporting Guideline for Reporting Cross-sectional Studies Checklist

	Item No.	Recommendation	Page No.
**Title and abstract**	1	(*a*) Indicate the study's design with a commonly used term in the title or the abstract	2
		(*b*) Provide in the abstract an informative and balanced summary of what was done and what was found	3
**Introduction**			
Background/rationale	2	Explain the scientific background and rationale for the investigation being reported	4
Objectives	3	State specific objectives, including any prespecified hypotheses	4
**Methods**			
Study design	4	Present key elements of study design early in the paper	5
Setting	5	Describe the setting, locations, and relevant dates, including periods of recruitment, exposure, follow-up, and data collection	5
Participants	6	(*a*) Give the eligibility criteria, and the sources and methods of selection of participants	5
Variables	7	Clearly define all outcomes, exposures, predictors, potential confounders, and effect modifiers. Give diagnostic criteria, if applicable	7
Data sources/ measurement	8[a]	For each variable of interest, give sources of data and details of methods of assessment (measurement). Describe comparability of assessment methods if there is more than one group	6
Bias	9	Describe any efforts to address potential sources of bias	8
Study size	10	Explain how the study size was arrived at	9
Quantitative variables	11	Explain how quantitative variables were handled in the analyses. If applicable, describe which groupings were chosen and why	8
Statistical methods	12	(*a*) Describe all statistical methods, including those used to control for confounding	8
		(*b*) Describe any methods used to examine subgroups and interactions	8
		(*c*) Explain how missing data were addressed	9
		(*d*) If applicable, describe analytical methods taking account of sampling strategy	5
		(*e*) Describe any sensitivity analyses	8
**Results**			
Participants	13*	(*a*) Report numbers of individuals at each stage of study - e.g., numbers potentially eligible, examined for eligibility, confirmed eligible, included in the study, completing follow-up, and analysed	9
		(*b*) Give reasons for non-participation at each stage	9
		(*c*) Consider use of a flow diagram	9
Descriptive data	14*	(*a*) Give characteristics of study participants (e.g., demographic, clinical, social) and information on exposures and potential confounders	9
		(*b*) Indicate number of participants with missing data for each variable of interest	9
Outcome data	15*	Report numbers of outcome events or summary measures	10
Main results	16	(*a*) Give unadjusted estimates and, if applicable, confounder-adjusted estimates and their precision (e.g., 95% confidence interval). Make clear which confounders were adjusted for and why they were included	10
		(*b*) Report category boundaries when continuous variables were categorized	9

		(*c*) If relevant, consider translating estimates of relative risk into absolute risk for a meaningful time period	10
Other analyses	17	Report other analyses done - e.g., analyses of subgroups and interactions, and sensitivity analyses	13
**Discussion**			
Key results	18	Summarise key results with reference to study objectives	13
Limitations	19	Discuss limitations of the study, taking into account sources of potential bias or imprecision. Discuss both direction and magnitude of any potential bias	17
Interpretation	20	Give a cautious overall interpretation of results considering objectives, limitations, multiplicity of analyses, results from similar studies, and other relevant evidence	14
Generalisability	21	Discuss the generalisability (external validity) of the study results	17
**Other information**			
Funding	22	Give the source of funding and the role of the funders for the present study and, if applicable, for the original study on which the present article is based	21

[a] Give information separately for exposed and unexposed groups.

eTable 3. Classification of BP by the 2003 NIH/NHLBI, 2004 NIH/NHLBI, 2017 AAP, and 2017 ACC/AHA Guidelines[a]

	SBP/DBP Percentile				
**Classification**	**2004 NIH/NHLBI for Children <17 y**	**2017 AAP for Children <13 y**	**2017 AAP for Children 13-17 y**	**2003 NIH/NHLBI for Adults ≥18 y**	**2017 ACC/AHA for Adults ≥18 y**
Normal	<90th	<90th	<120/<80 mmHg	<120/<80 mmHg	<120/<80 mmHg
Elevated BP	≥90th to <95th or 120/80 mmHg to <95th (whichever is lower)	≥90th to <95th or 120/80 mmHg to <95th (whichever is lower)	120/<80 to 129/<80 mmHg	120/80 to 139/89 mmHg	120/<80 to 129/<80 mmHg
Stage 1 hypertension	≥95th to <95th + 5 mmHg	≥95th to <95th + 12 mmHg or 130/80-139/89 mmHg (whichever is lower)	130/80 to 139/89 mmHg	140/90 to 159/99 mmHg	130/80 to 139/89 mmHg
Stage 2 hypertension	>99th + 5 mmHg	≥95th + 12 mmHg or ≥140/90 mmHg (whichever is lower)	≥140/≥90 mmHg	≥160/≥100 mmHg	≥140/≥90 mmHg

Abbreviations: AAP, American Academy of Pediatrics; ACC, American College of Cardiology; AHA, American Heart Association; BP, blood pressure; DBP, diastolic BP; NIH/NHLBI, National Institutes of Health's National Heart, Lung, and Blood Institute; SBP, systolic BP.

[a] Adapted from Ref. 33, 34, 46, and 47.

eTable 4. Trends in Age-Adjusted Means or % (95% CIs) of Cardiovascular Parameters, Cardiovascular Risk Factors, and Hypertension and Diabetes Treatment and Control Among US Adolescents Aged 12 to 19 Years by Age and Racial/Ethnic Groups, 2001 to March 2020[a-c]

Characteristics	2001-2004	2005-2008	2009-2012	2013-2016	2017-March 2020	Relative % (95% CIs) change per 4-year cycle[b]	*P* for trend[b]
**Cardiovascular parameters**							
Systolic BP, mmHg							
Overall	108.8 (108.2 to 109.4)	109.9 (109.0 to 110.8)	108.7 (108.0 to 109.4)	109.0 (108.4 to 109.6)	108.8 (108.1 to 109.6)	0.0 (-0.5 to 0.4)	.81
Female	105.9 (105.1 to 106.7)	107.6 (106.7 to 108.5)	105.6 (104.7 to 106.5)	106.3 (105.6 to 107.0)	105.6 (104.4 to 106.7)	-0.1 (-1.0 to 0.8)	.70
Male	111.5 (110.8 to 112.3)	112.1 (111.2 to 113.1)	111.6 (110.9 to 112.3)	111.6 (110.9 to 112.4)	111.9 (111.0 to 112.8)	0.0 (-0.2 to 0.3)	.69
Non-Hispanic White	108.5 (107.8 to 109.3)	109.7 (108.5 to 110.9)	108.3 (107.4 to 109.2)	108.3 (107.4 to 109.2)	109.4 (108.1 to 110.8)	0.0 (-0.6 to 0.6)	.93
Non-Hispanic Black	111.0 (110.3 to 111.7)	112.2 (111.3 to 113)	111.3 (110.3 to 112.2)	110.9 (109.8 to 112.1)	110.2 (109.0 to 111.3)	-0.2 (-0.8 to 0.5)	.44
Mexican American	108.4 (107.8 to 109)	109.7 (108.7 to 110.6)	108.3 (107.0 to 109.6)	109.8 (108.9 to 110.7)	107.3 (106.1 to 108.6)	0.1 (-0.9 to 1.0)	.85
Other[d]	107.9 (106.3 to 109.5)	108.5 (106.9 to 110.1)	108.0 (106.8 to 109.2)	108.8 (108 to 109.7)	107.2 (106.2 to 108.1)	-0.1 (-1.0 to 0.7)	.61
Diastolic BP, mmHg							
Overall	61.5 (60.8 to 62.2)	60.8 (60.0 to 61.7)	60.1 (58.8 to 61.4)	60.0 (59.2 to 60.8)	64.4 (63.8 to 65.0)	1.1 (-1.5 to 3.8)	.28
Female	62.6 (61.9 to 63.2)	62.3 (61.2 to 63.3)	60.2 (58.9 to 61.6)	61.1 (60.2 to 61.9)	64.8 (63.9 to 65.8)	0.4 (-2.1 to 2.9)	.68
Male	60.4 (59.5 to 61.2)	59.5 (58.6 to 60.4)	59.9 (58.6 to 61.2)	59.0 (58.0 to 60.1)	64.0 (63.2 to 64.8)	1.4 (-1.3 to 4.3)	.19
Non-Hispanic White	61.6 (60.7 to 62.5)	61.3 (60.2 to 62.3)	60.9 (59.3 to 62.5)	60.5 (59.5 to 61.6)	64.6 (63.6 to 65.6)	0.9 (-1.5 to 3.3)	.32
Non-Hispanic Black	61.3 (60.3 to 62.2)	61.4 (60.1 to 62.7)	60.1 (58.2 to 61.9)	58.8 (57.6 to 60.1)	65.1 (64.1 to 66.1)	1.2 (-2.2 to 4.6)	.36
Mexican American	60.3 (59.6 to 61.1)	59.2 (58.2 to 60.2)	57.3 (55.9 to 58.6)	59.9 (58.9 to 60.9)	63.8 (62.4 to 65.2)	0.6 (-2.5 to 3.9)	.56
Other[d]	62.0 (60.2 to 63.7)	59.4 (58.1 to 60.7)	59.7 (57.9 to 61.5)	59.5 (58.3 to 60.7)	63.9 (63.1 to 64.7)	1.5 (-1.8 to 5.0)	.25
Hemoglobin A_{1c}, %							
Overall	5.2 (5.2 to 5.2)	5.1 (5.1 to 5.2)	5.3 (5.2 to 5.3)	5.2 (5.2 to 5.2)	5.2 (5.2 to 5.3)	0.3 (-0.4 to 1.0)	.27

Female	5.2 (5.1 to 5.2)	5.1 (5.1 to 5.1)	5.3 (5.2 to 5.3)	5.2 (5.2 to 5.2)	5.3 (5.2 to 5.3)	0.5 (-0.3 to 1.4)	.14
Male	5.2 (5.2 to 5.2)	5.2 (5.1 to 5.2)	5.3 (5.2 to 5.3)	5.2 (5.2 to 5.3)	5.2 (5.2 to 5.3)	0.2 (-0.5 to 0.8)	.46
Non-Hispanic White	5.1 (5.1 to 5.2)	5.1 (5.0 to 5.1)	5.2 (5.2 to 5.3)	5.2 (5.1 to 5.2)	5.2 (5.1 to 5.2)	0.2 (-0.6 to 0.9)	.49
Non-Hispanic Black	5.3 (5.3 to 5.3)	5.3 (5.2 to 5.3)	5.3 (5.3 to 5.4)	5.3 (5.3 to 5.4)	5.4 (5.4 to 5.5)	0.5 (0.1 to 0.9)	.02
Mexican American	5.2 (5.2 to 5.2)	5.1 (5.1 to 5.2)	5.3 (5.2 to 5.3)	5.2 (5.2 to 5.3)	5.3 (5.2 to 5.3)	0.4 (-0.4 to 1.2)	.21
Other[d]	5.2 (5.2 to 5.3)	5.2 (5.1 to 5.2)	5.3 (5.2 to 5.3)	5.2 (5.2 to 5.3)	5.3 (5.2 to 5.3)	0.2 (-0.3 to 0.8)	.29
FPG, mg/dL							
Overall	92.1 (91.3 to 92.8)	96.0 (94.7 to 97.4)	93.5 (92.8 to 94.3)	95.9 (94.1 to 97.6)	97.5 (96.4 to 98.6)	1.3 (-0.2 to 2.8)	.07
Female	89.5 (88.6 to 90.3)	93.2 (91.8 to 94.7)	91.8 (90.8 to 92.8)	92.9 (91.7 to 94.1)	96.1 (94.9 to 97.3)	1.5 (0.2 to 2.9)	.04
Male	94.5 (93.5 to 95.6)	98.6 (96.2 to 101.1)	95.1 (94.2 to 96.0)	98.6 (95.6 to 101.6)	98.9 (97.6 to 100.2)	1.0 (-0.5 to 2.6)	.13
Non-Hispanic White	92.3 (91.3 to 93.3)	96.2 (93.7 to 98.8)	93.4 (92.5 to 94.2)	96.7 (93.4 to 100.0)	97.4 (95.8 to 99.0)	1.2 (-0.3 to 2.7)	.08
Non-Hispanic Black	90.0 (88.6 to 91.3)	93.6 (91.5 to 95.8)	92.0 (90.8 to 93.2)	93.3 (91.2 to 95.3)	98.0 (95.5 to 100.5)	1.6 (-0.3 to 3.5)	.08
Mexican American	93.4 (92.2 to 94.6)	97.9 (95.8 to 100.0)	95.5 (93.2 to 97.8)	97.4 (95.5 to 99.2)	98.5 (96.7 to 100.3)	1.2 (-0.1 to 2.6)	.07
Other[d]	92.4 (90.5 to 94.3)	96.5 (92.0 to 101.1)	93.5 (92.3 to 94.7)	94.8 (94.0 to 95.7)	96.7 (95.7 to 97.6)	1.2 (0.1 to 2.2)	.04
TC, mg/dL							
Overall	163.8 (162.1 to 165.4)	161.9 (160.4 to 163.4)	158.6 (156.6 to 160.6)	158.0 (156.3 to 159.8)	155.0 (152.5 to 157.5)	-1.3 (-1.8 to -0.9)	.003
Female	166.4 (164.5 to 168.3)	164.7 (162.3 to 167.1)	162.3 (159.8 to 164.8)	161.6 (159.4 to 163.8)	159.9 (156.6 to 163.2)	-1.0 (-1.3 to -0.7)	.001
Male	161.5 (158.9 to 164.0)	159.2 (156.9 to 161.6)	155.0 (152.7 to 157.3)	154.7 (152.8 to 156.6)	150.3 (148.0 to 152.5)	-1.7 (-2.4 to -0.9)	.006
Non-Hispanic White	164.1 (161.7 to 166.5)	162.1 (159.8 to 164.3)	159.8 (156.5 to 163.1)	157.5 (155.0 to 159.9)	155.2 (151.6 to 158.8)	-1.4 (-1.5 to -1.3)	<.001
Non-Hispanic Black	163.5 (161.9 to 165.0)	162.2 (159.9 to 164.6)	154.8 (152.0 to 157.6)	155.9 (152.9 to 158.8)	155.7 (151.6 to 159.8)	-1.6 (-2.9 to -0.2)	.04
Mexican American	162.2 (160.6 to 163.7)	161.1 (159.5 to 162.8)	158.8 (156.1 to 161.4)	157.9 (155.0 to 160.9)	154.4 (151.0 to 157.8)	-1.1 (-1.6 to -0.6)	.005
Other[d]	163.6 (158.8 to 168.5)	161.8 (158.7 to 165.0)	158.0 (155.1 to 161)	161.5 (157.8 to 165.1)	154.2 (150.5 to 158.0)	-1.2 (-2.9 to 0.6)	.13
HDL-C, mg/dL							
Overall	50.5 (50.1 to 50.9)	51.5 (50.9 to 52.2)	51.4 (50.6 to 52.1)	51.7 (50.8 to 52.7)	51.7 (50.6 to 52.8)	0.7 (-0.2 to 1.5)	.08

Female	53.6 (52.9 to 54.2)	54.0 (53.3 to 54.7)	53.4 (52.4 to 54.4)	53.7 (52.6 to 54.9)	54.7 (53.3 to 56.1)	0.3 (-0.5 to 1.1)	.38
Male	47.7 (47.0 to 48.5)	49.3 (48.5 to 50.1)	49.4 (48.5 to 50.4)	49.8 (48.5 to 51.1)	48.9 (48.0 to 49.7)	0.5 (-1.1 to 2.1)	.38
Non-Hispanic White	49.7 (49.0 to 50.4)	50.6 (49.8 to 51.4)	50.6 (49.4 to 51.8)	51.1 (49.5 to 52.7)	51.4 (49.5 to 53.3)	0.9 (0.2 to 1.5)	.02
Non-Hispanic Black	54.2 (53.3 to 55.1)	55.7 (54.7 to 56.7)	53.9 (52.5 to 55.2)	55.6 (54.3 to 56.8)	54.7 (53.0 to 56.3)	0.3 (-1.4 to 2.0)	.64
Mexican American	50.0 (49.2 to 50.8)	50.6 (49.4 to 51.7)	50.9 (49.7 to 52.1)	51.0 (50.0 to 51.9)	50.1 (48.1 to 52.2)	0.5 (-0.3 to 1.2)	.16
Other[d]	50.7 (49.3 to 52.0)	52.3 (50.4 to 54.1)	52.2 (50.6 to 53.9)	51.3 (50.2 to 52.5)	51.8 (50.7 to 52.9)	0.3 (-0.9 to 1.5)	.47
Non-HDL-C, mg/dL							
Overall	113.3 (111.5 to 115.0)	110.4 (108.8 to 111.9)	107.3 (105.2 to 109.3)	106.3 (104.4 to 108.3)	103.3 (100.9 to 105.8)	-2.2 (-2.8 to -1.6)	.001
Female	112.8 (110.8 to 114.9)	110.7 (108.5 to 113.0)	108.8 (106.3 to 111.4)	107.9 (105.6 to 110.2)	105.2 (101.8 to 108.6)	-1.6 (-2.0 to -1.2)	<.001
Male	113.7 (111.3 to 116.2)	109.9 (107.4 to 112.4)	105.6 (103.3 to 108.0)	104.9 (102.7 to 107.1)	101.5 (99.3 to 103.8)	-2.7 (-3.6 to -1.8)	.003
Non-Hispanic White	114.4 (111.8 to 116.9)	111.4 (109.3 to 113.6)	109.2 (105.7 to 112.7)	106.3 (103.3 to 109.4)	103.9 (100.3 to 107.5)	-2.4 (-2.5 to -2.2)	<.001
Non-Hispanic Black	109.3 (107.6 to 110.9)	106.6 (104.2 to 109.0)	101.0 (98.4 to 103.5)	100.3 (97.4 to 103.3)	101.0 (97.2 to 104.9)	-2.6 (-4.3 to -0.8)	.02
Mexican American	112.2 (110.7 to 113.6)	110.6 (108.5 to 112.7)	107.9 (105.3 to 110.4)	107.0 (103.9 to 110.0)	104.3 (100.4 to 108.1)	-1.7 (-2.1 to -1.3)	<.001
Other[d]	113.0 (108.4 to 117.6)	109.6 (105.7 to 113.4)	105.8 (103.0 to 108.5)	110.1 (106.6 to 113.7)	102.5 (99.1 to 105.9)	-1.8 (-4.9 to 1.4)	.17
LDL-C, mg/dL							
Overall	93.2 (91.3 to 95.1)	88.5 (87.1 to 89.9)	89.6 (87.7 to 91.4)	86.7 (84.8 to 88.7)	88.2 (85.1 to 91.2)	-1.5 (-3.9 to 1.0)	.15
Female	93.3 (90.9 to 95.8)	90.0 (87.5 to 92.6)	90.7 (88.9 to 92.6)	88.0 (85.2 to 90.8)	91.5 (87.7 to 95.4)	-0.9 (-3.1 to 1.2)	.25
Male	93.2 (90.2 to 96.2)	86.9 (84.4 to 89.4)	88.4 (85.2 to 91.5)	85.7 (82.7 to 88.7)	85.1 (81.9 to 88.3)	-1.9 (-4.3 to 0.5)	.09
Non-Hispanic White	93.4 (90.9 to 95.9)	89.0 (86.7 to 91.2)	91.2 (87.9 to 94.5)	87.2 (84.0 to 90.3)	87.4 (82.6 to 92.1)	-1.6 (-3.9 to 0.7)	.12
Non-Hispanic Black	93.4 (91.1 to 95.7)	90.4 (87.2 to 93.6)	88.8 (85.7 to 92.0)	85.3 (82.3 to 88.2)	92.2 (86.9 to 97.6)	-1.9 (-4.8 to 1.2)	.15
Mexican American	91.3 (89.4 to 93.2)	88.2 (86.3 to 90.2)	88.4 (84.4 to 92.5)	83.3 (80.3 to 86.3)	88.4 (84.0 to 92.8)	-1.8 (-4.4 to 0.9)	.12
Other[d]	93.6 (88.9 to 98.3)	84.1 (81.0 to 87.2)	86.9 (84.2 to 89.7)	89.8 (84.6 to 94.9)	86.9 (80.8 to 92.9)	-0.7 (-6.1 to 5.1)	.74
Triglycerides, mg/dL							
Overall	94.4 (89.5 to 99.4)	87.6 (83.3 to 91.9)	82.5 (78.5 to 86.6)	72.5 (68.2 to 76.8)	70.5 (66.1 to 74.9)	-7.5 (-9.7 to -5.2)	.002

Female	89.2 (83.2 to 95.2)	88.8 (83.0 to 94.7)	79.2 (73.5 to 84.9)	70.0 (65.2 to 74.8)	70.6 (65.3 to 75.9)	-6.9 (-11.1 to -2.5)	.02
Male	99.3 (90.9 to 107.7)	86.9 (81.3 to 92.5)	84.5 (78.8 to 90.2)	75.9 (68.8 to 83.1)	70.2 (65.6 to 74.9)	-7.7 (-10.2 to -5.2)	.003
Non-Hispanic White	97.0 (91.3 to 102.7)	91.5 (85.6 to 97.3)	83.3 (77.8 to 88.9)	73.7 (67.1 to 80.2)	72.5 (67.6 to 77.4)	-7.5 (-9.6 to -5.3)	.002
Non-Hispanic Black	69.0 (65.7 to 72.4)	65.8 (62.7 to 69.0)	66.4 (61.0 to 71.9)	53.4 (47.8 to 59.0)	52.8 (46.8 to 58.9)	-6.4 (-11.5 to -0.9)	.03
Mexican American	95.4 (89.6 to 101.2)	91.9 (87.7 to 96.1)	88.2 (79.7 to 96.8)	80.4 (74.2 to 86.5)	79.6 (70.8 to 88.4)	-5.0 (-7.0 to -3.1)	.004
Other[d]	108.2 (82.9 to 133.5)	90.5 (73.1 to 107.8)	90.2 (76.9 to 103.6)	78.0 (69.5 to 86.5)	69.3 (61.5 to 77.1)	-10.1 (-13.8 to -6.2)	.004
Body mass index, kg/m^2							
Overall	23.4 (23.1 to 23.7)	23.5 (23.1 to 23.8)	23.9 (23.5 to 24.2)	24.2 (23.8 to 24.6)	24.5 (24.1 to 24.9)	1.2 (0.7 to 1.6)	.003
Female	23.5 (23.1 to 24.0)	23.6 (23.1 to 24.0)	24.0 (23.5 to 24.4)	24.6 (24.0 to 25.1)	24.9 (24.4 to 25.5)	1.5 (0.7 to 2.3)	.009
Male	23.3 (23.0 to 23.7)	23.4 (22.9 to 23.9)	23.8 (23.3 to 24.3)	23.9 (23.4 to 24.4)	24.0 (23.5 to 24.6)	0.8 (0.5 to 1.2)	.004
Non-Hispanic White	23.2 (22.8 to 23.7)	23.0 (22.5 to 23.5)	23.5 (23.0 to 24.0)	23.6 (23.0 to 24.2)	24.1 (23.3 to 24.8)	0.9 (-0.1 to 1.8)	.06
Non-Hispanic Black	24.3 (23.9 to 24.7)	24.9 (24.5 to 25.3)	25.1 (24.5 to 25.8)	25.0 (24.1 to 25.9)	26.0 (25.3 to 26.8)	1.6 (0.6 to 2.5)	.01
Mexican American	23.8 (23.5 to 24.1)	24.6 (24.0 to 25.3)	24.5 (24.0 to 24.9)	25.6 (25.2 to 26.1)	25.9 (25.0 to 26.7)	2.3 (1.0 to 3.5)	.01
Other[d]	23.2 (22.5 to 23.9)	22.9 (22.2 to 23.6)	23.5 (22.8 to 24.2)	24.2 (23.4 to 24.9)	23.4 (22.7 to 24.1)	0.7 (-1.2 to 2.6)	.33
Weekly exercise time, min/wk							
Overall	177.2 (156.4 to 197.9)	830.3 (757.0 to 903.7)	1005.2 (920.4 to 1090.0)	1061.8 (988.9 to 1134.8)	1617.5 (1439.2 to 1795.8)	50.1 (0.0 to 149.3)	.08
Female	162.1 (132.2 to 192.0)	646.2 (587.0 to 705.3)	703.6 (634.3 to 772.8)	801.1 (721.7 to 880.4)	1413.4 (1116.9 to 1710.0)	38.5 (-14.8 to 125.3)	.12
Male	190.1 (170.0 to 210.3)	989.6 (871.1 to 1108.0)	1256.0 (1114.7 to 1397.2)	1289.9 (1184.7 to 1395.1)	1789.8 (1556.9 to 2022.6)	64.9 (-3.4 to 181.3)	.06
Non-Hispanic White	168.6 (144.4 to 192.9)	863.7 (756.3 to 971.2)	1089.8 (949.7 to 1230.0)	1092.3 (985.1 to 1199.5)	1612.7 (1405.6 to 1819.7)	54.7 (-8.7 to 162.1)	.08
Non-Hispanic Black	198.6 (165.4 to 231.9)	766.7 (610.7 to 922.8)	950.4 (854.3 to 1046.6)	1117.6 (984.6 to 1250.6)	1756.2 (1121.1 to 2391.2)	66.2 (6.4 to 159.4)	.04
Mexican American	157.8 (144.0 to 171.5)	764.9 (640.0 to 889.8)	896.6 (785.5 to 1007.8)	889.8 (748.4 to 1031.2)	1945.6 (1386.8 to 2504.5)	91.0 (13.4 to 221.9)	.03
Other[d]	212.6 (162.3 to 262.9)	832.6 (659.5 to 1005.7)	824.7 (721.5 to 927.9)	1081.1 (912.4 to 1249.9)	1270.5 (982.4 to 1558.5)	43.9 (-6.2 to 120.8)	.07
HEI-2015, points							

Overall	43.5 (42.9 to 44.2)	43.8 (43.0 to 44.6)	45.9 (45.0 to 46.8)	46.0 (45.2 to 46.8)	43.7 (42.6 to 44.7)	1.0 (0.0 to 3.9)	.34
Female	44.4 (43.5 to 45.4)	44.8 (43.5 to 46.1)	45.7 (44.9 to 46.6)	46.6 (45.6 to 47.6)	44.6 (43.3 to 46.0)	0.8 (-1.3 to 3.0)	.30
Male	42.8 (42.1 to 43.4)	42.8 (41.8 to 43.9)	46.1 (44.9 to 47.3)	45.3 (44.2 to 46.4)	42.7 (41.4 to 43.9)	0.9 (-2.5 to 4.5)	.45
Non-Hispanic White	43.3 (42.3 to 44.2)	43.5 (42.2 to 44.8)	45.4 (44.3 to 46.6)	45.8 (44.5 to 47.0)	42.4 (40.6 to 44.2)	1.0 (-2.3 to 4.4)	.43
Non-Hispanic Black	42.5 (41.6 to 43.4)	42.8 (42.1 to 43.5)	44.7 (43.3 to 46.0)	44.0 (43.3 to 44.8)	42.0 (39.4 to 44.7)	1.1 (-0.8 to 3.1)	.16
Mexican American	45.5 (44.4 to 46.5)	45.4 (43.7 to 47.1)	47.6 (46.2 to 49.1)	46.8 (45.6 to 48.1)	46.7 (44.9 to 48.5)	1.0 (-0.7 to 2.6)	.16
Other[d]	44.7 (43.0 to 46.3)	45.3 (43.8 to 46.9)	47.5 (46.0 to 49.0)	47.5 (45.9 to 49.1)	44.9 (42.9 to 46.9)	0.9 (-2.5 to 4.5)	.46
**Cardiovascular risk factors**							
Hypertension, %							
Overall	8.1 (6.9 to 9.4)	7.2 (5.4 to 9.0)	5.7 (4.8 to 6.7)	4.2 (3.2 to 5.1)	5.5 (3.7 to 7.3)	-15.3 (-26.8 to -1.9)	.04
Female	5.4 (3.8 to 7.0)	4.8 (3.3 to 6.3)	3.4 (2.3 to 4.5)	2.2 (1.1 to 3.3)	4.2 (1.7 to 6.8)	-16.3 (-36.6 to 10.3)	.13
Male	10.6 (8.4 to 12.7)	9.5 (7.0 to 11.9)	7.8 (6.0 to 9.6)	6.1 (4.6 to 7.5)	6.6 (4.7 to 8.5)	-13.7 (-21.1 to -5.5)	.01
Non-Hispanic White	8.8 (6.9 to 10.6)	6.9 (4.3 to 9.4)	5.2 (3.8 to 6.5)	3.4 (2.1 to 4.7)	6.1 (2.6 to 9.6)	-19.7 (-36.5 to 1.6)	.06
Non-Hispanic Black	9.7 (7.7 to 11.7)	10.2 (7.3 to 13.1)	9.1 (7.2 to 10.9)	6.6 (3.8 to 9.4)	6.3 (3.7 to 8.9)	-9.6 (-20.1 to 2.3)	.08
Mexican American	4.6 (3.3 to 6.0)	5.5 (3.9 to 7.2)	5.5 (3.6 to 7.5)	4.5 (2.7 to 6.4)	4.2 (1.7 to 6.8)	-1.4 (-14.8 to 14.1)	.78
Other[d]	6.3 (2.9 to 9.7)	6.6 (3.4 to 9.9)	4.3 (1.9 to 6.7)	4.4 (2.7 to 6.1)	3.6 (1.7 to 5.4)	-14.3 (-24.2 to -3.0)	.03
Hypertension (sensitivity analysis), %[e]							
Overall	4.8 (3.8 to 5.7)	4.0 (2.8 to 5.2)	3.4 (2.7 to 4.2)	2.2 (1.6 to 2.8)	3.0 (1.8 to 4.1)	-16.7 (-30.0 to -1.0)	.04
Elevated BP, %							
Overall	10.4 (8.9 to 11.8)	11.0 (8.8 to 13.2)	10.7 (8.7 to 12.7)	9.7 (8.5 to 11.0)	11.0 (8.7 to 13.4)	-0.7 (-6.6 to 5.6)	.73
Female	5.0 (4.1 to 5.9)	6.8 (4.9 to 8.7)	5.7 (4.0 to 7.4)	5.9 (4.7 to 7.1)	3.7 (1.4 to 5.9)	1.9 (-14.6 to 21.6)	.76
Male	15.5 (12.9 to 18.0)	15.0 (11.7 to 18.3)	15.5 (12.1 to 18.8)	13.5 (11.3 to 15.7)	18.0 (14.7 to 21.3)	1.3 (-10.0 to 14.1)	.74
Non-Hispanic White	10.0 (8.2 to 11.8)	10.6 (7.6 to 13.5)	9.6 (6.9 to 12.3)	8.9 (7.1 to 10.7)	13.5 (9.5 to 17.6)	1.8 (-12.7 to 18.6)	.74
Non-Hispanic Black	13.3 (11.4 to 15.3)	14.0 (12.0 to 16.1)	13.4 (11.0 to 15.8)	13.6 (10.9 to 16.4)	13.4 (10.2 to 16.6)	-0.1 (-2.9 to 2.8)	.92
Mexican American	10.9 (9.7 to 12.2)	11.1 (7.8 to 14.5)	12.4 (7.7 to 17.2)	10.6 (7.8 to 13.4)	6.5 (2.9 to 10.1)	-3.3 (-15.3 to 10.4)	.48
Other[d]	8.1 (4.7 to 11.6)	9.8 (5.1 to 14.6)	10.9 (7.9 to 13.8)	8.5 (6.6 to 10.4)	6.0 (3.1 to 8.9)	-6.0 (-26.7 to 20.6)	.49
Elevated BP (sensitivity analysis), %[e]							
Overall	14.8 (13.1 to 16.6)	15.9 (13.0 to 18.7)	14.2 (11.8 to 16.6)	12.9 (11.4 to 14.5)	14.6 (11.7 to 17.5)	-3.0 (-9.5 to 4.1)	.27
Diabetes, %							

Overall	0.7 (0.2 to 1.2)	1.1 (0.4 to 1.8)	0.5 (0.1 to 0.8)	1.3 (0.4 to 2.3)	1.2 (0.3 to 2.2)	13.1 (-28.8 to 79.7)	.46
Female	0.5 (0.0 to 1.1)	0.6 (0.1 to 1.0)	0.3 (0.0 to 0.6)	1.1 (0.1 to 2.1)	1.9 (0.4 to 3.4)	42.0 (-9.6 to 122.9)	.09
Male	0.9 (0.1 to 1.7)	1.6 (0.1 to 3.0)	0.7 (0.1 to 1.3)	1.5 (0.0 to 3.0)	0.6 (0.0 to 1.3)	-6.2 (-44.4 to 58.3)	.72
Non-Hispanic White	0.6 (0.0 to 1.4)	1.3 (0.1 to 2.5)	0.1 (0.0 to 0.4)	1.3 (0.0 to 2.8)	0.3 (0.0 to 0.9)	-4.8 (-65.2 to 160.4)	.89
Non-Hispanic Black	0.6 (0.0 to 1.3)	0.6 (0.0 to 1.3)	1.1 (0.0 to 2.4)	2.1 (0.0 to 4.1)	3.6 (0.8 to 6.4)	63.7 (33.4 to 100.9)	.005
Mexican American	1.1 (0.2 to 2.1)	1.1 (0.2 to 2.0)	1.5 (0.0 to 3.3)	1.4 (0.0 to 2.9)	4.0 (0.0 to 8.5)	29.2 (-5.5 to 76.7)	.08
Other[d]	0.7 (0.0 to 2.1)	0.7 (0.0 to 1.5)	0.2 (0.0 to 0.5)	1.0 (0.0 to 2.2)	0.0 (0.0 to 0.0)	-94.7 (-100.0 to 1.2e+04)	.31
Prediabetes, %							
Overall	12.5 (10.2 to 14.9)	25.1 (21.2 to 29.0)	19.4 (16.1 to 22.7)	28.6 (24.3 to 32.9)	37.6 (29.1 to 46.2)	24.0 (-4.9 to 61.7)	.08
Female	7.0 (4.9 to 9.1)	17.1 (13.5 to 20.7)	13.4 (9.9 to 16.9)	17.6 (13.9 to 21.4)	31.9 (22.2 to 41.7)	29.1 (-8.9 to 82.9)	.10
Male	17.8 (14.0 to 21.5)	32.5 (27.4 to 37.6)	25.0 (20.6 to 29.4)	39.6 (33.4 to 45.8)	43.5 (33.8 to 53.2)	20.4 (-6.2 to 54.5)	.10
Non-Hispanic White	13.0 (9.3 to 16.7)	23.7 (18.1 to 29.3)	15.6 (11.3 to 19.9)	26.9 (20.3 to 33.5)	35.8 (20.8 to 50.7)	21.1 (-12.1 to 66.8)	.15
Non-Hispanic Black	10.9 (7.8 to 14.1)	25.3 (20.5 to 30.1)	22.5 (16.9 to 28.0)	31.1 (23.0 to 39.2)	37.4 (26.9 to 47.9)	26.2 (-5.8 to 69.1)	.08
Mexican American	16.7 (12.6 to 20.7)	32.6 (24.9 to 40.4)	25.5 (19.8 to 31.2)	33.5 (25.0 to 42.0)	42.1 (28.0 to 56.1)	20.4 (-6.6 to 55.1)	.10
Other[d]	8.8 (4.9 to 12.8)	24.7 (14.5 to 34.9)	24.3 (16.3 to 32.3)	27.1 (20.7 to 33.6)	40.2 (32.4 to 47.9)	33.8 (5.5 to 69.5)	.03
Hyperlipidemia, %							
Overall	34.2 (30.9 to 37.5)	28.7 (24.4 to 33.1)	25.5 (23.0 to 28.0)	25.6 (22.1 to 29.1)	22.8 (18.7 to 26.8)	-9.8 (-15.2 to -4.0)	.01
Female	27.9 (21.3 to 34.6)	25.1 (19.7 to 30.5)	22.5 (17.8 to 27.2)	23.1 (18.3 to 27.8)	21.4 (16.0 to 26.8)	-5.9 (-10.3 to -1.2)	.03
Male	39.9 (35.4 to 44.5)	31.9 (26.5 to 37.3)	28.0 (23.9 to 32.0)	28.7 (22.8 to 34.6)	23.8 (16.6 to 31.0)	-12.2 (-19.2 to -4.6)	.02
Non-Hispanic White	36.6 (31.9 to 41.4)	31.1 (24.9 to 37.4)	25.7 (21.1 to 30.4)	27.1 (21.7 to 32.4)	21.3 (13.7 to 28.8)	-11.6 (-18.4 to -4.3)	.02
Non-Hispanic Black	23.7 (19.6 to 27.8)	24.0 (19.3 to 28.7)	20.9 (16.0 to 25.9)	16.5 (11.8 to 21.3)	22.8 (14.3 to 31.3)	-6.2 (-17.8 to 7.2)	.23
Mexican American	32.4 (29.1 to 35.6)	29.8 (25.3 to 34.3)	28.0 (20.6 to 35.4)	26.6 (21.3 to 31.9)	25.7 (17.9 to 33.5)	-6.1 (-7.5 to -4.6)	<.001
Other[d]	34.9 (27.7 to 42.1)	19.7 (12.8 to 26.6)	27.6 (21.4 to 33.9)	28.6 (21.3 to 36.0)	23.6 (14.6 to 32.5)	-6.5 (-24.0 to 15.1)	.38
Obesity, %							
Overall	16.0 (14.1 to 17.9)	16.3 (13.8 to 18.8)	17.4 (15.1 to 19.7)	18.9 (16.3 to 21.5)	20.3 (17.9 to 22.7)	6.4 (4.2 to 8.6)	.002
Female	16.0 (13.1 to 18.8)	16.7 (13.7 to 19.7)	17.7 (14.9 to 20.6)	19.9 (16.5 to 23.3)	21.0 (17.4 to 24.7)	7.6 (5.3 to 9.9)	.002
Male	16.0 (14.0 to 18.1)	16.1 (13.0 to 19.2)	17.0 (13.9 to 20.1)	18.0 (15.1 to 20.9)	19.7 (16.7 to 22.6)	5.1 (2.5 to 7.8)	.008
Non-Hispanic White	15.6 (12.8 to 18.3)	13.4 (10.0 to 16.9)	15.9 (12.3 to 19.5)	15.6 (11.7 to 19.5)	17.8 (13.7 to 21.9)	3.4 (-4.5 to 12.0)	.27
Non-Hispanic Black	19.1 (17.1 to 21.2)	23.2 (20.9 to 25.5)	21.8 (18.1 to 25.5)	22.9 (18.0 to 27.8)	27.0 (22.5 to 31.6)	7.4 (-1.3 to 16.8)	.07
Mexican American	16.7 (14.3 to 19.0)	20.6 (17.1 to 24.1)	20.3 (17.5 to 23.0)	25.6 (22.7 to 28.5)	29.4 (22.6 to 36.1)	14.6 (6.8 to 23.0)	.009

Other[d]	13.8 (10.0 to 17.5)	17.9 (14.3 to 21.6)	15.4 (12.0 to 18.7)	19.9 (14.8 to 25.0)	15.5 (11.5 to 19.5)	2.6 (-13.0 to 21.0)	.66
Overweight, %							
Overall	21.1 (19.3 to 22.8)	21.8 (20.3 to 23.4)	22.1 (20.2 to 24.1)	23.6 (21.7 to 25.4)	24.8 (21.4 to 28.2)	3.9 (2.3 to 5.5)	.004
Female	19.4 (17.1 to 21.7)	20.9 (18.3 to 23.6)	20.5 (17.6 to 23.5)	24.0 (21.2 to 26.8)	24.5 (20.4 to 28.7)	6.4 (2.0 to 11.0)	.02
Male	22.6 (20.3 to 24.9)	22.6 (20.1 to 25.0)	23.7 (20.5 to 26.9)	23.1 (20.6 to 25.7)	24.9 (20.5 to 29.3)	1.8 (-0.7 to 4.4)	.11
Non-Hispanic White	20.7 (18.1 to 23.3)	22.3 (20.2 to 24.4)	20.3 (17.1 to 23.6)	22.4 (19.6 to 25.2)	25.4 (19.4 to 31.4)	2.7 (-3.9 to 9.7)	.29
Non-Hispanic Black	20.5 (18.4 to 22.6)	20.7 (17.3 to 24.1)	23.9 (21.2 to 26.6)	23.5 (21.0 to 26.1)	21.6 (17.2 to 26.0)	3.8 (-2.7 to 10.7)	.16
Mexican American	24.0 (21.6 to 26.5)	26.4 (23.5 to 29.3)	27.3 (23.2 to 31.3)	26.8 (24.3 to 29.3)	28.8 (24.5 to 33.2)	3.7 (0.2 to 7.4)	.04
Other[d]	20.9 (14.4 to 27.4)	17.1 (14.1 to 20.1)	22.8 (18.6 to 27.1)	24.5 (21.1 to 28.0)	21.7 (16.4 to 27.0)	8.4 (-8.2 to 27.9)	.22
Cigarette use, %							
Overall	18.0 (15.7 to 20.3)	17.1 (15.2 to 19.1)	13.8 (12.2 to 15.4)	5.9 (4.7 to 7.1)	3.5 (2.0 to 5.0)	-27.3 (-49.3 to 4.1)	.07
Female	17.3 (14.5 to 20.0)	16.0 (13.7 to 18.3)	12.1 (10.7 to 13.5)	4.5 (2.8 to 6.1)	2.2 (1.2 to 3.2)	-29.8 (-54.3 to 8.0)	.08
Male	18.6 (15.6 to 21.5)	18.0 (15.3 to 20.6)	15.1 (12.5 to 17.6)	7.3 (5.3 to 9.3)	4.7 (1.5 to 7.9)	-22.5 (-42.6 to 4.7)	.07
Non-Hispanic White	19.6 (16.2 to 23.0)	19.7 (16.9 to 22.6)	15.9 (13.8 to 18.0)	7.4 (5.2 to 9.6)	4.8 (2.1 to 7.5)	-23.1 (-45.3 to 8.3)	.09
Non-Hispanic Black	11.6 (9.9 to 13.3)	8.0 (6.5 to 9.4)	8.1 (5.6 to 10.6)	4.4 (2.6 to 6.2)	1.1 (0.1 to 2.1)	-28.5 (-47.0 to -3.5)	.04
Mexican American	15.5 (12.7 to 18.2)	15.5 (11.8 to 19.2)	13.2 (10.2 to 16.3)	3.7 (2.4 to 5.1)	3.0 (1.2 to 4.8)	-28.7 (-55.3 to 13.8)	.10
Other[d]	18.8 (12.3 to 25.3)	15.9 (12.3 to 19.6)	11.9 (8.1 to 15.7)	4.4 (2.4 to 6.4)	1.8 (0.4 to 3.2)	-39.5 (-59.7 to -9.2)	.03
Inactive physical activity, %							
Overall	83.0 (80.7 to 85.3)	51.1 (48.6 to 53.7)	37.2 (34.8 to 39.6)	37.0 (33.7 to 40.2)	9.5 (4.2 to 14.8)	-29.0 (-41.0 to -14.7)	.01
Female	85.1 (82.0 to 88.2)	56.2 (51.8 to 60.7)	46.8 (43.0 to 50.5)	44.8 (39.8 to 49.8)	12.7 (5.6 to 19.8)	-23.6 (-34.5 to -10.8)	.01
Male	81.2 (78.5 to 83.9)	46.6 (43.8 to 49.4)	28.8 (25.4 to 32.2)	30.2 (26.2 to 34.1)	6.8 (1.0 to 12.7)	-34.7 (-47.5 to -18.7)	.008
Non-Hispanic White	83.6 (80.7 to 86.4)	50.8 (47.1 to 54.4)	32.3 (29.0 to 35.6)	33.6 (28.5 to 38.7)	8.2 (0.8 to 15.7)	-33.3 (-44.7 to -19.6)	.006
Non-Hispanic Black	82.1 (78.8 to 85.3)	51.3 (43.5 to 59.1)	43.0 (38.3 to 47.8)	41.1 (36.1 to 46.0)	19.8 (9.2 to 30.5)	-23.8 (-32.8 to -13.6)	.006
Mexican American	83.9 (81.4 to 86.4)	55.9 (50.8 to 61.0)	43.9 (40.3 to 47.4)	45.5 (41.0 to 50.1)	8.4 (1.9 to 14.8)	-23.1 (-34.7 to -9.5)	.01
Other[d]	80.4 (74.3 to 86.5)	48.8 (39.8 to 57.8)	45.5 (40.3 to 50.7)	36.9 (32.9 to 41.0)	9.2 (2.5 to 15.8)	-23.9 (-34.3 to -11.7)	.01
Poor diet quality, %							
Overall	76.1 (74.0 to 78.2)	73.5 (70.4 to 76.5)	67.8 (64.1 to 71.5)	67.8 (64.8 to 70.7)	71.7 (68.5 to 74.9)	-2.3 (-5.8 to 1.3)	.13
Female	73.6 (70.2 to 77.1)	70.8 (65.5 to 76.1)	68.8 (65.7 to 71.9)	65.0 (61.3 to 68.7)	69.4 (65.2 to 73.6)	-2.3 (-5.6 to 1.2)	.13
Male	78.3 (75.9 to 80.6)	76.1 (72.4 to 79.7)	66.7 (61.3 to 72.2)	70.5 (66.7 to 74.3)	74.0 (70.4 to 77.7)	-2.1 (-5.9 to 1.9)	.19
Non-Hispanic White	77.2 (74.1 to 80.3)	73.4 (68.3 to 78.4)	70.0 (64.6 to 75.4)	69.5 (64.2 to 74.7)	74.8 (69.1 to 80.4)	-1.8 (-5.6 to 2.1)	.23
Non-Hispanic Black	80.2 (77.2 to 83.2)	79.1 (75.8 to 82.5)	70.6 (64.9 to 76.3)	70.2 (65.9 to 74.5)	76.4 (69.5 to 83.2)	-3.2 (-7.6 to 1.5)	.12

Mexican American	68.0 (63.5 to 72.4)	67.6 (63.3 to 71.9)	62.6 (55.6 to 69.7)	63.9 (59.8 to 67.9)	63.7 (56.7 to 70.8)	-2.0 (-4.0 to 0.0)	.049
Other[d]	72.7 (66.2 to 79.2)	71.6 (64.2 to 79.1)	60.7 (54.0 to 67.4)	63.8 (59.3 to 68.3)	68.4 (61.9 to 74.9)	-2.6 (-9.0 to 4.4)	.32
**Hypertension and diabetes treatment and control**							
Hypertension treatment, %							
Overall	9.6 (3.5 to 15.8)	3.0 (0.8 to 5.3)	8.9 (2.5 to 15.3)	13.9 (5.2 to 22.6)	6.0 (1.4 to 10.6)	6.2 (-44.9 to 100.0)	.79
Hypertension treatment (sensitivity analysis), %[e]							
Overall	19.3 (10.3 to 28.4)	8.0 (3.1 to 12.8)	16.1 (6.9 to 25.4)	21.5 (7.2 to 35.7)	28.3 (21.4 to 35.1)	19.2 (-12.7 to 62.7)	.17
Diabetes treatment, %							
Overall	51.0 (23.3 to 78.7)	61.5 (36.6 to 86.4)	62.7 (62.7 to 62.7)	72.1 (53.5 to 90.6)	26.5 (0.0 to 54.7)	5.6 (-21.3 to 41.8)	.60
BP control, %							
Overall	75.7 (56.8 to 94.7)	85.0 (64.2 to 100.0)	79.9 (57.3 to 100.0)	57.9 (15.2 to 100.0)	73.5 (40.3 to 100.0)	-1.7 (-12.5 to 10.3)	.66
BP control (sensitivity analysis), %[e]							
Overall	90.5 (75.9 to 100.0)	100.0 (100.0 to 100.0)	79.9 (57.3 to 100.0)	95.4 (88.6 to 100.0)	82.8 (57.1 to 100.0)	NA	NA
Glycemic control, %							
Overall	11.8 (0.0 to 31.5)	28.2 (9.1 to 47.4)	59.6 (59.6 to 59.6)	50.2 (29.9 to 70.4)	62.7 (62.7 to 62.7)	NA	NA

Abbreviations: BP, blood pressure; CI, confidence interval; FPG, fasting plasma glucose; HDL-C, high-density lipoprotein cholesterol; HEI-2015, Healthy Eating Index-2015; LDL-C, low-density lipoprotein cholesterol; NA, not applicable; NIH/NHLBI, National Institutes of Health's National Heart, Lung, and Blood Institute; non-HDL-C, non-high-density lipoprotein cholesterol; TC, total cholesterol.

SI conversions: to convert glucose to mmol/L, multiply by 0.0555; TC, HDL-C, non-HDL-C, and LDL-C to mmol/L, multiply by 0.0259; triglycerides to mmol/L, multiply by 0.0113.

[a] Nationally representative estimates of US adolescents aged 12-19 years from the 2001-March 2020 National Health and Nutrition Examination Survey.

[b] All estimates were age-standardized to the 2000 Census population using the age groups of 12 to 14, 15 to 17, and 18 to 19 years.

[c] Relative % change per 4-year cycle and *P* for trend were calculated by the Joinpoint Regression Program. A joinpoint regression model with heteroscedastic and uncorrected errors was fitted. The default maximum number of joinpoints (0 joinpoints, corresponding to a straight line) was allowed to avoid possible overfitting. The optimal fitting model was chosen by performing 4499 permutation tests based on the Monte Carlo method, adjusting for multiple tests. Parameters were estimated using weighted least squares, with weights proportional to the inverse of the variance of ln-transformed age-standardized prevalence rate at each 4-year cycle.

[d] Race/ethnicity was based on self-report. The non-Hispanic Asian category was not available before 2011 due to the survey design, and thus estimates could not be presented separately. All other racial/ethnic groups were grouped as 'Other'.

[e] A sensitivity analysis was performed by defining high BP following the 2003 NIH/NHLBI and 2004 NIH/NHLBI guidelines. The definition of hypertension was identical to that used in the main analysis. Hypertension was considered controlled if (1) BP was reduced to <95th percentile in adolescents aged <18 years or (2) BP was reduced to <140/90 mmHg in adolescents aged 18-19 years.

eTable 5. Age-Adjusted Rates of Hypertension and Diabetes Treatment and Control by Subgroups Among US Adolescents Aged 12 to 19 Years, 2001 to March 2020[a]

	Adolescents receiving treatment, % (95% CIs)[b]			
**Characteristics**	**Hypertension treatment[c]**	**Diabetes treatment[c]**	**BP control[d]**	**Glycemic control[d]**
No.[e]	68	40	45	18
Age group, y				
12-14	4.5 (0.8-8.3)	61.2 (27.5-94.9)	79.1 (47.8-100.0)	7.4 (0.0-19.2)
15-17	13.5 (7.5-19.5)	45.6 (21.3-69.9)	68.6 (50.1-87.2)	82.2 (61.5-100.0)
18-19	5.7 (2.9-8.4)	66.9 (45.2-88.6)	70.1 (46.4-93.9)	37.6 (4.9-70.3)
Sex				
Female	14.6 (8.9-20.3)	51.2 (28.6-73.8)	90.0 (80.4-99.7)	52.2 (35.0-69.4)
Male	6.6 (3.4-9.8)	60.1 (42.8-77.4)	58.3 (36.3-80.3)	38.5 (26.9-50.1)
Race/ethnicity[f]				
Non-Hispanic White	9.4 (4.9-13.9)	63.2 (45.2-81.3)	74.3 (54.3-94.3)	44.3 (32.7-55.8)
Non-Hispanic Black	6.9 (3.7-10.2)	61.2 (44.8-77.7)	56.3 (27.4-85.3)	42.7 (20.6-64.9)
Mexican American	7.4 (2.9-11.9)	44.9 (24.9-64.9)	78.3 (59.1-97.5)	71.9 (45.0-98.7)
Other	5.2 (1.6-8.7)	56.2 (44.7-67.8)	80.0 (57.0-100.0)	78.5 (50.7-100.0)
Birth country				
US born	8.5 (5.6-11.4)	56.4 (39.5-73.2)	72.9 (56.2-89.6)	45.7 (32.7-58.6)
Non-US born	3.2 (0.1-6.2)	62.7 (62.7-62.7)	70.5 (34.7-100.0)	NA
Income to poverty ratio, %				
<130	10.8 (5.3-16.4)	34.9 (17.4-52.4)	75.2 (50.3-100.0)	79.5 (55.2-100.0)
130-349	6.4 (2.8-10.0)	55.7 (31.7-79.7)	71.8 (53.0-90.7)	44.0 (33.7-54.3)
≥350	9.3 (3.3-15.3)	77.6 (54.1-100.0)	68.6 (53.1-84.0)	18.8 (0.0-41.6)
Insurance status				
Uninsured	4.5 (0.0-12.3)	33.7 (23.1-44.3)	NA	53.6 (0.0-100.0)
Insured	8.9 (6.0-11.8)	63.0 (49.0-77.1)	72.1 (55.5-88.7)	42.4 (29.6-55.2)

Abbreviations: BP, blood pressure; CI, confidence interval; NA, not applicable.

[a] Nationally representative estimates of US adolescents aged 12-19 years from the 2001-March 2020 National Health and Nutrition Examination Survey.

[b] All estimates were age-standardized to the 2000 Census population using the age groups of 12 to 14, 15 to 17, and 18 to 19 years.

[c] Hypertension treatment was defined as current use of antihypertensive medications and was evaluated among adolescents with hypertension (n = 901). Diabetes treatment was defined as current use of antidiabetic medications and was evaluated among adolescents with diabetes (n = 84).

[d] Control was evaluated among adolescents receiving treatment (n = 68 for hypertension and n = 40 for diabetes). Hypertension was considered controlled if (1) BP was reduced to <90th percentile in adolescents aged <13 years, (2) BP was reduced to <90th percentile and <130/80 mmHg in adolescents aged 13-17 years, or (3) BP was reduced to <130/80 mmHg in adolescents aged 18-19 years. Diabetes was considered controlled if hemoglobin A_{1c} was reduced to <7%.

[e] Unweighted number of adolescents receiving treatment or risk factor controlled.

[f] Race/ethnicity was based on self-report. The non-Hispanic Asian category was not available before 2011 due to the survey design, and thus estimates could not be presented separately. All other racial/ethnic groups were grouped as ‘Other’.

eTable 6. Adjusted ORs for Hypertension and Diabetes Treatment and Control by Subgroups Among US Adolescents Aged 12 to 19 Years, 2001 to March 2020[a]

Characteristics	Adolescents receiving treatment, adjusted ORs (95% CIs)[b]			
	Hypertension treatment[c]	Diabetes treatment[c]	BP control[d]	Glycemic control[d]
No.[e]	68	40	45	18
Age group, y				
12-14	1 [Reference]	1 [Reference]	1 [Reference]	1 [Reference]
15-17	4.47 (1.40, 14.29)	0.76 (0.11, 5.32)	0.30 (0.01, 6.16)	28.13 (1.14, 692.67)
18-19	1.86 (0.56, 6.22)	1.46 (0.30, 7.19)	0.34 (0.02, 5.66)	6.34 (0.71, 56.60)
Sex				
Female	1 [Reference]	1 [Reference]	1 [Reference]	1 [Reference]
Male	0.30 (0.14, 0.64)	0.92 (0.26, 3.28)	0.23 (0.02, 2.16)	0.21 (0.02, 2.20)
Race/ethnicity[f]				
Non-Hispanic White	1 [Reference]	1 [Reference]	1 [Reference]	1 [Reference]
Non-Hispanic Black	0.80 (0.38, 1.68)	0.23 (0.04, 1.40)	0.57 (0.07, 4.42)	2.06 (0.18, 23.50)
Mexican American	0.98 (0.41, 2.35)	0.26 (0.04, 1.55)	0.80 (0.09, 6.72)	5.77 (0.37, 89.82)
Other	0.58 (0.23, 1.46)	0.64 (0.06, 7.48)	1.45 (0.11, 18.52)	2.46 (0.08, 79.03)
Birth country				
US born	1 [Reference]	1 [Reference]	1 [Reference]	1 [Reference]
Non-US born	0.53 (0.18, 1.58)	3.98 (0.34, 45.91)	0.64 (0.01, 30.33)	NA
Income to poverty ratio, %				
<130	1 [Reference]	1 [Reference]	1 [Reference]	1 [Reference]
130-349	0.57 (0.23, 1.46)	2.06 (0.44, 9.60)	0.15 (0.01, 1.83)	0.35 (0.02, 5.57)
≥350	0.74 (0.24, 2.27)	5.56 (0.55, 55.81)	0.16 (0.01, 2.16)	0.00 (0.00, 0.00)
Insurance status				
Uninsured	1 [Reference]	1 [Reference]	NA	1 [Reference]
Insured	3.34 (0.42, 26.71)	5.23 (0.34, 80.40)	NA	1.36 (0.01, 195.61)

Abbreviations: BP, blood pressure; CI, confidence interval; NA, not applicable; OR, odds ratio.

[a] Nationally representative estimates of US adolescents aged 12-19 years from the 2001-March 2020 National Health and Nutrition Examination Survey.

[b] Adjusted ORs with 95% CIs were adjusted for age, sex, and race/ethnicity groups.

[c] Hypertension treatment was defined as current use of antihypertensive medications and was evaluated among adolescents with hypertension (n = 901). Diabetes treatment was defined as current use of antidiabetic medications and was evaluated among adolescents with diabetes (n = 84).

[d] Control was evaluated among adolescents receiving treatment (n = 68 for hypertension and n = 40 for diabetes). Hypertension was considered controlled if (1) BP was reduced to <90th percentile in adolescents aged <13 years, (2) BP was reduced to <90th percentile and <130/80 mmHg in adolescents aged 13-17 years, or (3) BP was reduced to <130/80 mmHg in adolescents aged 18-19 years. Diabetes was considered controlled if hemoglobin A_{1c} was reduced to <7%.

[e] Unweighted number of adolescents receiving treatment or risk factor controlled.

[f] Race/ethnicity was based on self-report. The non-Hispanic Asian category was not available before 2011 due to the survey design, and thus estimates could not be presented separately. All other racial/ethnic groups were grouped as ‘Other’.

eTable 7. Comparison of Baseline Characteristics Between the Included and Excluded Study Population[a]

Characteristics	Included participants (n = 15155)[b]	Excluded participants (n = 368)[b]	*P* value[c]
Age, mean, y	15.4 (15.4-15.5)	14.1 (13.9-14.3)	<.001
Age group, y			<.001
12-14	38.7 (37.5-39.8)	58.5 (51.9-65.2)	
15-17	38.4 (37.3-39.4)	38.1 (31.4-44.9)	
18-19	23.0 (21.9-24.0)	3.3 (1.4-5.3)	
Sex			.08
Female	48.7 (47.6-49.8)	54.0 (48.3-59.6)	
Male	51.3 (50.2-52.4)	46.0 (40.4-51.7)	
Race/ethnicity[d]			.16
Non-Hispanic White	57.4 (54.7-60.1)	58.5 (51.6-65.5)	
Non-Hispanic Black	14.4 (12.8-16.0)	12.2 (8.9-15.5)	
Mexican American	13.3 (11.7-14.9)	10.3 (6.7-14.0)	
Other	14.9 (13.6-16.2)	18.9 (13.3-24.6)	
Birth country	(n = 15150)	(n = 368)	.11
US born	91.5 (90.6-92.3)	94.3 (91.4-97.1)	
Non-US born	8.5 (7.7-9.4)	5.7 (2.9-8.6)	
Income to poverty ratio, %	(n = 13950)	(n = 303)	.02
<130	30.5 (28.6-32.3)	21.8 (15.2-28.3)	
130-349	37.0 (35.4-38.6)	33.6 (25.3-41.8)	
≥350	32.5 (30.5-34.6)	44.7 (34.1-55.3)	
Insurance status	(n = 15016)	(n = 360)	.20
Uninsured	12.1 (11.1-13.1)	9.0 (4.9-13.1)	
Insured	87.9 (86.9-88.9)	91.0 (86.9-95.1)	

Abbreviations: CI, confidence interval; NHANES, National Health and Nutrition Examination Survey.

[a] Nationally representative estimates of US adolescents aged 12-19 years from the 2001-March 2020 NHANES. The sample size for each 4-year interval was unweighted, whereas all other numbers were weighted means or percentages with 95% CIs.

[b] Unweighted sample size.

[c] Baseline characteristics of the included and excluded participants were compared by one-way analysis of variance for continuous variables and chi-square test for categorical variables.

[d] Race/ethnicity was based on self-report. The non-Hispanic Asian category was not available before 2011 due to the survey design, and thus estimates could not be presented separately. All other racial/ethnic groups were grouped as ‘Other’.

eTable 8. Baseline Characteristics of the Excluded Study Population, 2001 to March 2020[a]

Characteristics	2001-2004 (n = 91)[b]	2005-2008 (n = 76)[b]	2009-2012 (n = 49)[b]	2013-2016 (n = 55)[b]	2017-March 2020 (n = 97)[b]	*P* for trend[c]
Age, mean, y	14.8 (14.3-15.2)	14.2 (13.7-14.7)	14.0 (13.5-14.6)	13.5 (13.2-13.8)	14.1 (13.7-14.5)	.049
Age group, y						
12-14	40.7 (27.2-54.3)	57.9 (42.8-72.9)	69.1 (54.9-83.3)	71.3 (60.9-81.7)	53.8 (39.2-68.4)	.34
15-17	49.4 (33.7-65.2)	37.0 (22.3-51.7)	24.4 (11.8-37.1)	28.7 (18.3-39.1)	46.2 (31.6-60.8)	>.99
18-19	9.8 (1.7-17.9)	5.2 (0.5-9.8)	6.5 (0.0-14.5)	0.0 (0.0-0.0)	0.0 (0.0-0.0)	<.001
Sex						
Female	50.9 (39.3-62.5)	60.5 (48.4-72.7)	70.5 (56.4-84.6)	54.2 (39.4-68.9)	43.8 (33.8-53.8)	.10
Male	49.1 (37.5-60.7)	39.5 (27.3-51.6)	29.5 (15.4-43.6)	45.8 (31.1-60.6)	56.2 (46.2-66.2)	.10
Race/ethnicity[d]						
Non-Hispanic White	57.8 (42.6-73.1)	48.3 (34.4-62.1)	60.4 (43.2-77.7)	66.5 (50.0-82.9)	58.0 (45.3-70.7)	.52
Non-Hispanic Black	14.6 (8.4-20.8)	15.4 (7.1-23.6)	14.2 (6.1-22.4)	7.8 (2.1-13.5)	11.3 (4.3-18.3)	.28
Mexican American	10.0 (5.8-14.2)	11.8 (5.5-18.1)	6.1 (0.2-12.0)	10.0 (4.4-15.7)	12.1 (2.4-21.8)	.73
Other	17.6 (5.6-29.6)	24.6 (9.2-39.9)	19.2 (3.7-34.8)	15.7 (3.3-28.1)	18.6 (9.2-27.9)	.73
Birth country						
US born	86.5 (75.2-97.7)	93.6 (84.8-100.0)	93.4 (87.6-99.2)	99.0 (97.1-100.0)	95.7 (91.7-99.6)	.07
Non-US born	13.5 (2.3-24.8)	6.4 (0.0-15.2)	6.6 (0.8-12.4)	1.0 (0.0-2.9)	4.3 (0.4-8.3)	.07
Income to poverty ratio, %	(n = 76)	(n = 63)	(n = 41)	(n = 46)	(n = 77)	
<130	16.7 (6.5-27.0)	25.3 (10.5-40.1)	35.3 (17.7-52.9)	21.9 (5.4-38.4)	15.9 (5.1-26.6)	.50
130-349	43.4 (25.5-61.3)	42.2 (22.4-61.9)	49.7 (24.1-75.4)	19.2 (5.7-32.7)	26.3 (13.0-39.6)	.03
≥350	39.9 (21.6-58.2)	32.5 (14.7-50.4)	15.0 (0.0-33.7)	58.9 (38.9-78.9)	57.8 (36.9-78.7)	.048
Insurance status	(n = 83)	(n = 76)	(n = 49)	(n = 55)	(n = 97)	
Uninsured	13.4 (4.2-22.6)	11.4 (1.4-21.4)	11.2 (0.0-22.5)	12.4 (0.0-25.3)	2.7 (0.0-6.3)	.047
Insured	86.6 (77.4-95.8)	88.6 (78.6-98.6)	88.8 (77.5-100.0)	87.6 (74.7-100.0)	97.3 (93.7-100.0)	.047

Abbreviations: CI, confidence interval; NHANES, National Health and Nutrition Examination Survey.

[a] Nationally representative estimates of US adolescents aged 12-19 years from the 2001-March 2020 NHANES. The sample size for each 4-year interval was unweighted, whereas all other numbers were weighted means or percentages with 95% CIs.

[b] Unweighted sample size.

[c] *F* test based on weighted linear regression or Wald test based on logistic regression.

[d] Race/ethnicity was based on self-report. The non-Hispanic Asian category was not available before 2011 due to the survey design, and thus estimates could not be presented

separately. All other racial/ethnic groups were grouped as ‘Other’.
